# Immunomodulators and advanced therapies for maintenance of remission in Crohn’s disease: systematic review and network meta-analysis

**DOI:** 10.1177/17562848261470683

**Published:** 2026-07-27

**Authors:** Vassiliki Sinopoulou, Morris Gordon, Shiyao Liu, Daniel Arruda Navarro Albuquerque, Aderonke Ajiboye, Sudheer Kumar Vuyyuru, Shellie Jane Radford, Gordon William Moran

**Affiliations:** School of Medicine, University of Lancashire, Preston, UK; School of Medicine, University of Lancashire, Harrington Building HA340, Preston PR1 2HE, UK; School of Medicine, University of Lancashire, Preston, UK; School of Medicine, University of Lancashire, Preston, UK; School of Medicine, University of Lancashire, Preston, UK; Faculty of Medicine & Health Sciences, University of Nottingham, Nottingham, UK; Faculty of Medicine & Health Sciences, University of Nottingham, Nottingham, UK; Faculty of Medicine & Health Sciences, University of Nottingham, Nottingham, UK

**Keywords:** advanced treatments, biologics, Crohn’s disease, immunomodulators, inflammatory bowel disease, network meta-analysis, small molecules, systematic review

## Abstract

**Background:**

With the range of options available for the treatment of Crohn’s disease, therapy selection can be challenging in clinical practice. Comparative efficacy data are needed to clarify the relative position of the increasingly complex portfolio of advanced therapies and guide decision-making.

**Objectives:**

Our aim was to compare all advanced and immunomodulator treatments for efficacy and safety in maintenance of remission.

**Design:**

Systematic review and network meta-analysis.

**Data sources and methods:**

We searched databases up to June 2025. Our outcomes were clinical relapse, loss of response, endoscopic relapse, and safety outcomes. We estimated risk ratio (RR) and 95% confidence interval (CI). We used GRADE to assess certainty of results, and surface under the cumulative ranking curve for ranking treatments.

**Results:**

A total of 37 randomised controlled trials with 7415 participants were included. Interventions ranged between 22 weeks and 2 years. Adalimumab probably prevents clinical relapse over placebo (RR 0.68, 95% CI 0.54–0.84, Number Needed to Treat (NNT) = 2, moderate effect magnitude). CT-P13 (RR 0.52, 95% CI 0.38–0.71) and infliximab (RR 0.61, 95% CI 0.49–0.76) may prevent clinical relapse. Upadacitinib is more effective than placebo at preventing loss of clinical response (RR 0.64, 95% CI 0.57–0.72, NNT = 2, small magnitude), while CT-P13 (RR 0.46, 95% CI 0.35–0.53, NNT = 1, large magnitude), natalizumab (RR 0.54, 95% CI 0.43–0.68, NNT = 2, moderate magnitude), certolizumab (RR 0.57, 95% CI 0.47–0.7), NNT = 2, moderate magnitude), adalimumab (RR 0.68, 95% CI 0.61–0.75, NNT = 2, small effect magnitude) and ustekinumab (RR 0.73, 95% CI 0.61–0.86, NNT = 2, small magnitude) probably prevent loss of clinical response. Infliximab (RR 0.7, 95% CI 0.59–0.82) and vedolizumab (RR 0.82, 95% CI 0.68–0.99) may prevent loss of clinical response. Endoscopic relapse could not be analysed. Safety evidence is uncertain, but treatments appear generally safe in the short term.

**Conclusion:**

Adalimumab has moderate certainty for maintaining clinical remission with a moderate effect size. Novel therapies seem to have similar effect sizes, though imprecision due to limited evidence precludes further conclusions.

**Trial registration:**

https://knowledge.lancashire.ac.uk/id/eprint/53237/

## Plain Language Summary

Comparing treatments for maintaining Crohn’s remission

Keeping Crohn’s disease under control over time can be challenging in clinical practice. Data comparing the different treatments is lacking. Our aim was to compare the different treatments and find out how reliable and safe they are. We found all available randomised clinical trials (RCTs) up to June 2025. We looked for data on disease relapse, loss of response to previous treatment, and safety of the treatments. We performed a technique called network meta-analysis. We assessed the quality of the included studies and the certainty of our results. We also ranked the treatments from best to worst. Our review found 37 RCTs with 7415 participants. The therapies were given for periods between 22 weeks and 2 years. Adalimumab is fairly reliable at keeping symptoms in remission, with a moderate benefit. Upadacitinib is very reliable at keeping patients responding to treatment, but its benefit is smaller. Other medicines may help, but we’re less certain about them. In the short term, most treatments seem safe, though the evidence is limited. These findings give the clearest picture so far to help patients and clinicians choose medicines to maintain remission or response. Better studies comparing treatments head-to-head are still needed.

## Introduction

Crohn’s disease (CD) is a chronic, relapsing and remitting inflammatory bowel disease characterised by transmural inflammation that can affect any site along the gastrointestinal tract.^1^ Its pathophysiology is thought to involve a complex interaction of genetic, immunological and environmental factors. The incidence and prevalence of CD varies across geographic regions, with the highest epidemiological burden in Europe, Oceania and North America.^2^ In the United Kingdom, a recent study estimated the overall incidence of CD to be 10.21 per 100,000 person-years, with the regions of Northern Ireland, Scotland and Northwest having the highest rates.^3^

In the past two decades, an increased understanding of the immunological mechanisms involved in the pathogenesis of CD has led to the development of several advanced therapies that selectively block key mediators of inflammation.^4^ The tumour necrosis factor-alpha (TNF-α) antagonists (infliximab, adalimumab and certolizumab pegol) were the first group of biologics approved for the treatment of CD. Since the approval of TNF-α antagonists, other advanced therapies with different mechanisms of action have been developed. These include anti-integrins (natalizumab and vedolizumab), IL-12/IL-23 p40 antagonists (ustekinumab), IL-23 p19 antagonists (risankizumab, guselkumab and mirikizumab) and Janus kinase (JAK) 1 inhibitors (upadacitinib) which can be administered orally.

With the range of options available for the treatment of CD, therapy selection can be challenging in clinical practice. Comparative efficacy data are needed to clarify the relative position of the increasingly complex portfolio of advanced therapies and guide decision-making regarding CD therapeutic algorithms. The appropriate method for comparing the efficacy of different drugs is head-to-head blinded controlled trials. However, practical limitations make it difficult to conduct multiple trials that will include all available treatment options. So far, only three head-to-head trials have been completed in CD, SEAVUE, SEQUENCE and VIVID-1.^5–7^ Network meta-analysis (NMA), in which multiple treatments are compared via direct comparisons of interventions within randomised controlled trials (RCTs) and indirect comparisons across trials, using a common index treatment, is a statistical analysis approach that can be used to address this problem.^8^ Although several NMAs have been published, the evolving data landscape necessitates updated analyses. Also, previous NMAs did not include immunomodulators or a combination of immunomodulators with advanced therapies.^9,10^

Our aim was to perform an NMA and compare clinical, endoscopic and safety outcomes of all available advanced therapies and immunomodulators, including combination therapies of advanced therapies and immunomodulators, for maintaining remission, with the purpose of improving therapeutic decision-making.

## Materials and methods

A protocol for this review was made publicly available prospectively through the University of Lancashire’s online repository.^11^ We followed the Preferred Reporting Items for Systematic Reviews and Meta-analyses (PRISMA) reporting guidelines.^12^ The present work was exempt from ethics approval.

### Literature search

Our literature search methods were outlined in our protocol and our methods were similar to our NMA for induction of clinical remission.^11,13^ We searched MEDLINE, EMBASE, Cochrane Library and Web of Science from inception to June 2025 (eAppendix 1).

### Study selection

We included adult participants diagnosed with CD in remission or response to previous treatment, as defined by the included studies. Phase III and IIb RCTs comparing advanced therapies with any other active comparator, placebo or no treatment for maintenance of remission or response in CD, cluster RCTs and the pre-crossover phases of crossover RCTs were eligible.^11,13^ RCT data published in scientific abstracts, press releases, or on trial registration websites were also eligible. Non-randomised or quasi-randomised trials, such as non-randomised maintenance phase (treat-through design), non-randomised long-term follow-ups or non-randomised control groups were excluded. Trials comparing different dosages of the same treatment, treatment strategies, dose escalation or trough levels were excluded.^11,13^

Any advanced therapy and biosimilars were eligible, including TNF-α inhibitors, anti-integrins, IL-12/23p40 antagonists, IL-23p19 antagonists, JAK inhibitors and others. All types of administration routes and dose regimens were considered for inclusion.^11,13^

Title/abstract and full-text screening were performed in duplicate by two experienced reviewers (M.G., V.S.) and consensus was reached by discussion in cases of disagreement.^11,13^

### Outcomes

Our primary outcome was clinical relapse, defined as the number of patients who experienced relapse based on the included studies’ definitions. Secondary outcomes were loss of clinical response, endoscopic relapse, withdrawals due to adverse events (WAEs), serious adverse events (SAEs) and total adverse events (TAEs).^11,13^ When studies reported the rates of remission or response as outcomes, we used the inverted rates to infer relapses or loss of response.

### Outcome thresholds

Our Inflammatory Bowel Disease (IBD) outcome thresholds for the assessment of imprecision of magnitude effects have been published and described in other publications (eTable 5).^13–16^ Prospectively setting magnitude of effect thresholds is the recommended approach for assessing imprecision for NMA results and guideline recommendations. ^17^

### Data extraction and risk of bias assessment

Data extraction included demographic and baseline characteristics, intervention details and outcome data. Risk of bias assessment was assessed using the Cochrane risk of bias 1 tool.^18^ They were performed in duplicate by two experienced reviewers (M.G., V.S.) and disagreements were resolved by discussion and consensus with a senior author.

### Statistical analysis

Our statistical analysis plan was outlined in our protocol and our methods were similar to our NMA for induction of clinical remission.^11,13^

Outcome results were expressed in risk ratios (RRs) with corresponding 95% confidence intervals (CIs), using a random effects model modified intention-to-treat analysis for all randomised participants whether they received their assigned interventions or not. We counted participants with missing study data as treatment failures, and if they withdrew from the study, as having potentially experienced adverse effects.^11^ Our unit of analysis was the participant. NMA was not attempted if the number of studies that could be connected was not sufficient (less than 10 studies to form a network).^11^ We combined intervention data from different dosages of the same medication. We used the primary endpoint data of the included studies, or if the primary endpoint was undefined, the last data point of the randomisation period for our analyses.

We employed a frequentist NMA model.^19^ Transitivity was assessed by comparison of the distribution of potential effect modifiers.^11^ Statistical heterogeneity was assessed with the *I*^2^ statistic and with the loop-specific approach.^11^ Ranking of treatments was based on surface under the cumulative ranking curve (SUCRA). The index intervention for comparison of treatment effects was the pooled placebo effect from included studies. Publication bias was assessed via funnel plots when data from at least 10 studies were available. The presence of small-study effects was assessed via Comparison-adjusted funnel plots were used to assess small-study effects. The R statistical software and netmeta package were used.^11,13,20,21^

### Subgroup and sensitivity analyses

Per our protocol, the following were planned for the outcome of clinical relapse.^11,13^

*Subgroup analyses*:

- Patients naïve to advanced treatments (>50% of all participants being naïve) versus patients that have failed advanced treatments previously (>50% of all participants being not naïve).
- Separating the dosages for each treatment that were combined in the main analysis.
- Per identical timepoints of measurement of the outcome.

*Sensitivity analyses*:

- Removal of studies where the population is mixed regarding the use of purine analogues (if >20% of all participants on concomitant purine analogues, studies were removed).
- Removal of studies with mixed populations of patients in a state of both clinical remission and clinical response at baseline randomisation, resulting in an analysis of all patients classified as being in remission at baseline.

We also conducted three additional sensitivity analyses which were not planned in our protocol. One was for the advanced treatments only, removing any studies including patients exposed solely to purine analogues, a second was that studies published in 2003 were removed (we chose the year when infliximab was approved for use as the beginning of the ‘biologic’ era), and a third was removing small studies with a total of 50 participants or less.

### GRADE assessment for the certainty of evidence

We assessed the certainty of our outcome results with GRADE.^22^ The GRADE methodology we used was the same as the one described in our systematic review and NMA for induction of remission.^13^ Two senior review authors (M.G., V.S.) assessed the evidence certainty, and consensus was reached by discussion in case of disagreements. We used ‘**G**RADEing **O**f **R**elative effect **D**iagram **O**f **N**MA’ (GORDON) Plots to present outcome results, ranking and GRADE certainty at once in single figures for each outcome.^8^

## Results

**Figure 1.**
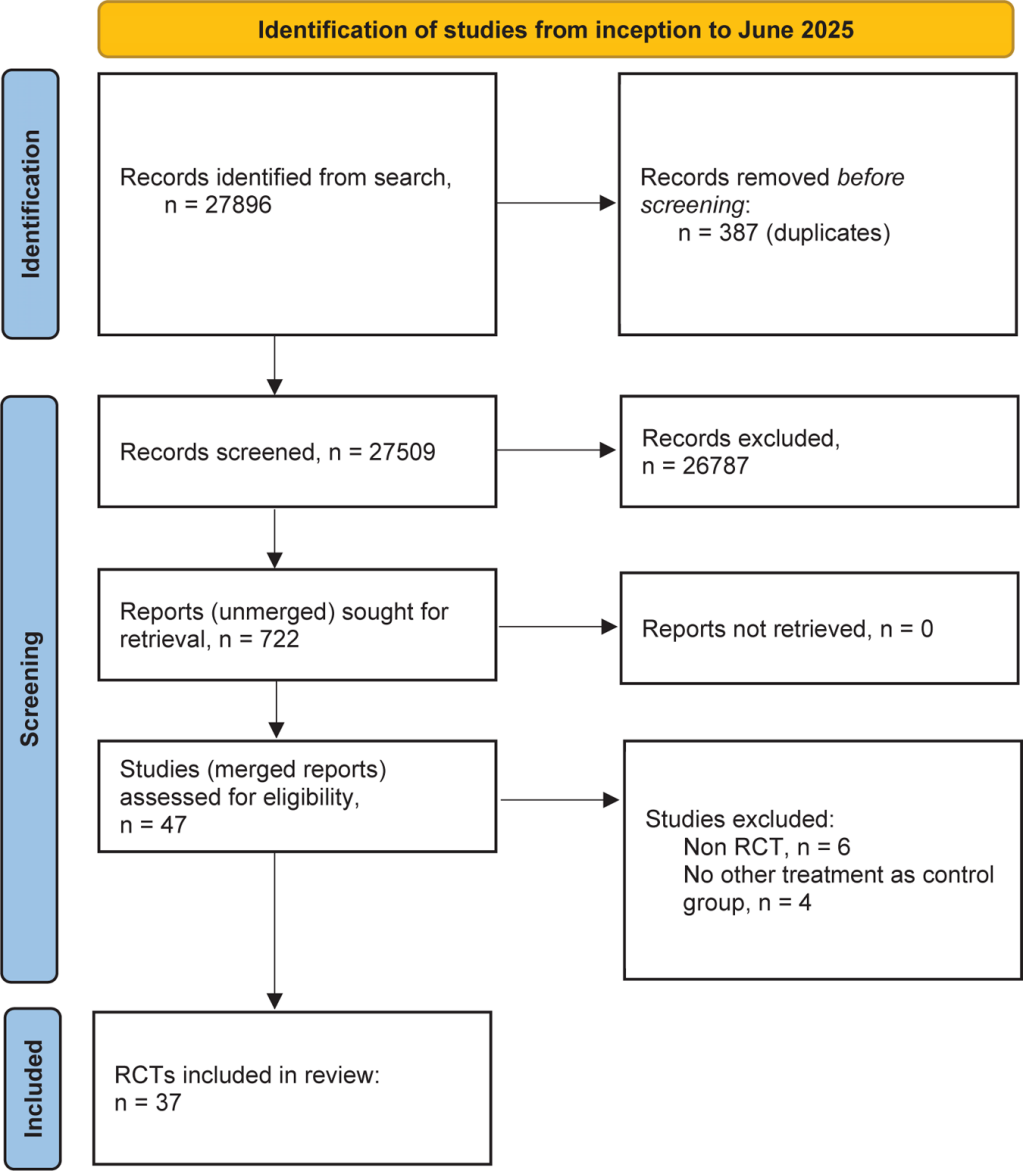
PRISMA flow diagram. PRISMA, Preferred Reporting Items for Systematic Reviews and Meta-analyses.

The systematic search retrieved 27,896 records. We screened 47 studies at full-text screening (merged records), of which 10 were excluded. We included 37 RCTs (*n* = 7415) (Figure 1, eAppendix 2, eTable 4). Fourteen studies with non-randomised treat-through designs for maintenance were excluded [ClinicalTrials.gov identifier: NCT03234907].^5,6,23–33^ We did not identify any cluster trials or pre-crossover data from crossover studies.

The range of the follow-ups in the included studies was 22–108 weeks (eTable 1). In the advanced therapies RCTs, remission/response was induced using the same advanced therapies that the studies examined for maintenance of remission/response. No studies published before 1999 were on patients naïve to advanced treatments. In most studies, the Crohn’s Disease Activity Index (CDAI) scale was used to measure clinical disease activity. Clinical remission definitions were based on CDAI scores <150. Clinical response was based on CDAI scale reductions of >70 and/or >100 points (eTable 2). Supplemental Material 1 details the data on included studies’ participant and disease characteristics, outcome definitions and included studies’ methods (eTables 1 and 2). The assumption of transitivity was supported by an even distribution of potential effect modifiers in participant, disease and intervention characteristics and outcome definitions (eTables 1–3).

Supplemental Material 1 provides summary of findings tables with GRADE judgements for all review outcomes and additional plots and analysis results. Supplemental Material 2 provides our risk of bias assessment judgements.

Of the 37 RCTs, 25 (67.5%) were funded fully or partially by pharmaceutical companies, 7 (18.9%) were funded by public or charity funds and 5 (13.5%) did not state funding sources. Of the 25 RCTs involving advanced therapies, 2 RCTs on infliximab withdrawal and 1 on an infliximab biosimilar were funded by public funds,^34–36^ while 1 RCT did not state a funding source.^37^ All other advanced therapy RCTs were fully or partially funded by pharmaceutical companies.

### Clinical relapse

**Figure 2.**
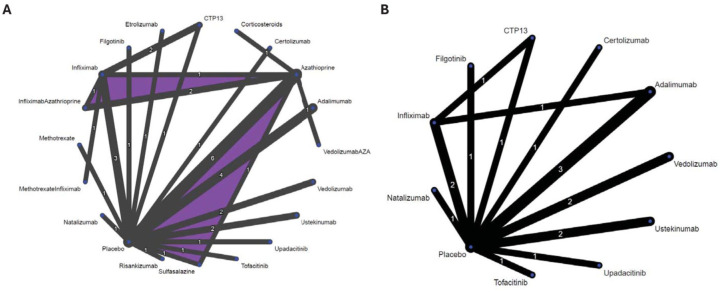
Network plots for clinical relapse (a) and loss of clinical response (b).

Thirty-three studies (*n* = 7354) assessing 20 interventions were included in the clinical relapse NMA (Figure 2(a)). The cumulative placebo relapse rate was 68.1% (range 23.2%–92%). Network heterogeneity was 55.7% (*I*^2^).

**Figure 3.**
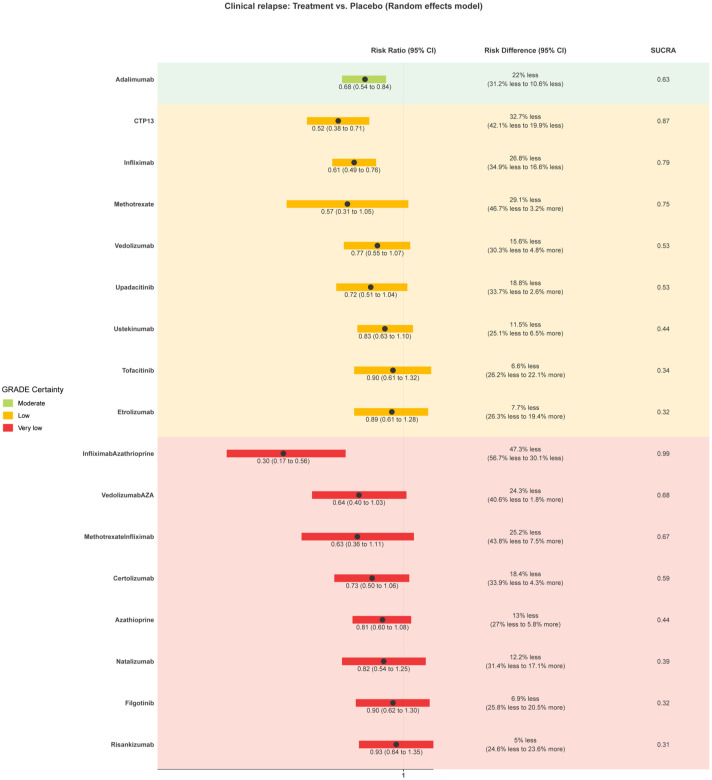
GORDON plot of clinical relapse network results compared with placebo. GORDON, Grading of relative effect diagram of NMA.

None of the interventions were rated high for GRADE certainty. One treatment, adalimumab, was rated at moderate GRADE certainty and is probably more effective in preventing clinical relapse compared to placebo (RR 0.68, 95% CI 0.54–0.84, NNT = 2, moderate magnitude of effect). Two treatments were rated at low GRADE certainty as maybe being more effective at preventing clinical relapse compared to placebo. In order of SUCRA ranking, these are CT-P13 (RR 0.52, 95% CI 0.38–0.71, large magnitude of effect), and infliximab (RR 0.61, 95% CI 0.49–0.76, moderate magnitude of effect). Six treatments were rated at low GRADE certainty as maybe being similar to placebo at preventing clinical relapse: methotrexate (RR 0.57, 95% CI 0.31–1.05), vedolizumab (RR 0.77, 95% CI 0.55–1.07) upadacitinib (RR 0.72, 95% CI 0.51–1.04), ustekinumab (RR 0.83, 95% CI 0.63–1.1), tofacitinib (RR 0.9, 95% CI 0.61–1.32) and etrolizumab (RR 0.89, 95% CI 0.61–1.28). The other treatments had very low GRADE certainty of evidence, and no conclusions can be drawn about them (Table 1, Figure 3, eFigure 3).

**Table 1.** Summary of findings table for clinical relapse.

Clinical relapse							
Patient or population: people with Crohn’s disease in remission or response to treatment prior to randomisation, not biologically naïve							
Settings: hospital setting							
Intervention: advanced therapies/purine analogues/methotrexate							
Comparison: placebo							
Treatment	Network evidence		Anticipated absolute effects for network estimate			NNT (95% CI)	Notes
	RR (95% CI)	Certainty	Risk with placebo^a^	Risk with agent^b^ (95% CI)	% Risk difference with agent^c^ (95% CI)		
Infliximab with purine analogues	0.30 (0.17–0.56)	Very low	681 per 1000	209 per 1000 (115–378)	47.3% less (56.7% less to 30.1% less)	NA	The data are very uncertain.
		⊕⊖⊖⊖					
CT-P13 (ifx biosimilar)	0.52 (0.38–0.71)	Low	681 per 1000	365 per 1000 (270–493)	32.7% less (42.1% less to 19.9% less)	NA	Maybe large effect better than placebo (moderate to large).
		⊕⊕⊖⊖					
Infliximab	0.61 (0.49–0.76)	Low	681 per 1000	419 per 1000 (338–513)	26.8% less (34.9% less to 16.6% less)	NA	Maybe moderate effect better than placebo (small to large).
		⊕⊕⊖⊖					
Methotrexate	0.57 (0.31–1.05)	Low	681 per 1000	385 per 1000 (216–695)	29.1% less (46.7% less to 3.2% more)	NA	Maybe the same as placebo. Effect ranging from largely less than placebo to trivial effect more than placebo.
		⊕⊕⊖⊖					
Methotrexate with infliximab	0.63 (0.36–1.11)	Very low	681 per 1000	432 per 1000 (250–756)	24.3% less (43.8% less to 7.5% more)	NA	The data are very uncertain.
		⊕⊖⊖⊖					
Vedolizumab azathioprine	0.64 (0.40–1.03)	Very low	681 per 1000	439 per 1000 (284–695)	24.3% less (40.6% less to 1.8% more)	NA	The data are very uncertain.
		⊕⊖⊖⊖					
Adalimumab	0.68 (0.54–0.84)	Moderate	681 per 1000	459 per 1000 (371–567)	22% less (31.2% less to 10.6% less)	2 (2–3)	Probably moderate effect better than placebo (small to large).
		⊕⊕⊕⊖					
Upadacitinib	0.72 (0.51–1.04)	Low	681 per 1000	486 per 1000 (351–689)	18.8% less (33.7% less to 2.6% more)	NA	Maybe the same as placebo with an effect ranging from trivially more to largely less.
		⊕⊕⊖⊖					
Certolizumab	0.73 (0.5–1.06)	Low	681 per 1000	493 per 1000 (344–702)	18.8% less (33.9% less to 4.3% more)	NA	The data is very uncertain.
		⊕⊖⊖⊖					
Treatment	Network evidence		Anticipated absolute effects for network estimate			NNT (95% CI)	Notes
	RR (95% CI)	Certainty	Risk with placebo^a^	Risk with agent^b^ (95% CI)	% Risk difference with agent^c^ (95% CI)		
Vedolizumab	0.77 (0.55–1.07)	Low	681 per 1000	520 per 1000 (378–716)	15.6% less (30.3% less to 4.8% more)	NA	Maybe the same as placebo. Effect ranging from moderately less to trivially more.
		⊕⊕⊖⊖					
Ustekinumab	0.83 (0.63–1.1)	Low	681 per 1000	560 per 1000 (432–729)	11.5% less (25.1% less to 6.5% more)	NA	Maybe the same as placebo. Effect ranging from moderately less to trivially more.
		⊕⊕⊖⊖					
Purine analogues	0.81 (0.6–1.08)	Very low	681 per 1000	554 per 1000 (419–743)	13% less (27% less to 5.8% more)	NA	The data is very uncertain.
		⊕⊖⊖⊖					
Natalizumab	0.82 (0.54–1.25)	Very low	681 per 1000	554 per 1000 (371–830)	12.2% less (31.4% less to 17.1% more)	NA	The data is very uncertain.
		⊕⊖⊖⊖					
Etrolizumab	0.89 (0.61–1.28)	Low	681 per 1000	601 per 1000 (425–851)	7.7% less (26.3% less to 19.4% more)	NA	Maybe the same as placebo. Effect ranging from moderately less to moderately more.
		⊕⊕⊖⊖					
Filgotinib	0.90 (0.62–1.30)	Very low	681 per 1000	613 per 1000 (415–885)	6.9% less (25.8% less to 20.5% more)	NA	The data are very uncertain.
		⊕⊖⊖⊖					
Tofacitinib	0.9 (0.61–1.32)	Low	681 per 1000	608 per 1000 (425–878)	6.6% less (26.2% less to 22.1% more)	NA	Maybe the same as placebo. Effect ranging from moderately less to small effect more.
		⊕⊕⊖⊖					
Risankizumab	0.93 (0.64–1.35)	Very low	681 per 1000	628 per 1000 (439–891)	5% less (24.6% less to 23.6% more)	NA	The data are very uncertain.
		⊕⊖⊖⊖					

GRADE Working Group grades of evidence – High certainty: we are very confident that the true effect lies close to that of the estimate of the effect. Moderate certainty: we are moderately confident in the effect estimate; the true effect is likely to be close to the estimate of the effect, but there is a possibility that it is substantially different. Low certainty: our confidence in the effect estimate is limited; the true effect may be substantially different from the estimate of the effect. Very low certainty: we have very little confidence in the effect estimate; the true effect is likely to be substantially different from the estimate of the effect.

a The risk with placebo has been calculated based on the cumulative placebo rates of all studies with a placebo arm.

b The risk with treatment has been calculated by multiplying the risk with control by the RR (95% CI). If the calculation results in more than 1000 per 1000 people, the number has been capped to 1000. Numbers have been rounded up to the closest whole number.

c The % risk difference has been calculated by subtracting the risk with control from the risk with treatment (95% CI) and dividing by 10. If the calculation results in more than 100%, the number has been capped to 100%. Numbers have been rounded up to the closest whole number.

CI, confidence interval; NA, Not Applicable; RR, risk ratio.

The pre-planned subgroup analysis for patients naïve to advanced treatments was not possible as none of the advanced therapies RCTs were on participants naïve to these treatments. Subgroup analyses per dosages and time-point of measurement were not possible due to lack of data. The pre-planned sensitivity analyses were not possible either, as there were not sufficient numbers of studies with patients receiving less than 20% concomitant purine analogues (azathioprine/6-MP), or being solely in remission at baseline, for networks to be established. Visual inspection of the two additional sensitivity analyses – removing patients who were not receiving any advanced therapies and removing studies taking place before 2003 – did not reveal major differences from the main analysis (eFigure 4). The comparison-adjusted funnel plot for the assessment of small-study size was not significantly asymmetrical (eFigure 5).

### Loss of clinical response

Seventeen studies (*n* = 4292) assessing 11 interventions were included in the loss of clinical response NMA (Figure 2(b)). The cumulative placebo loss of response rate was 69% (range 38.8%–92%). Network heterogeneity was 0% (*I*^2^).

**Figure 4.**
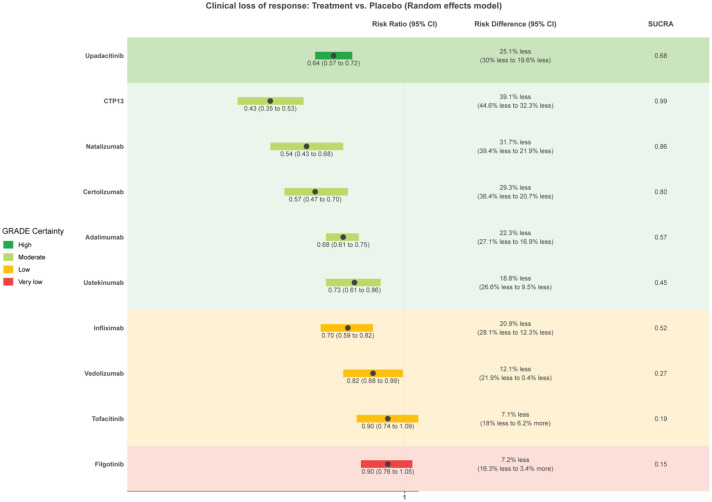
GORDON plot of clinical loss of response network results compared with placebo. GORDON, Grading of relative effect diagram of NMA.

One of the interventions, upadacitinib, was rated high GRADE certainty as being more effective than placebo at preventing loss of clinical response (RR 0.64, 95% CI 0.57–0.72, NNT = 2, small magnitude of effect). Five treatments were rated at moderate GRADE certainty and are probably more effective at preventing loss of clinical response compared to placebo. In order of SUCRA ranking these are CT-P13 (RR 0.43, 95% CI 0.35–0.53, NNT = 1, large effect magnitude), natalizumab (RR 0.54, 95% CI 0.43–0.68, NNT = 2, moderate effect magnitude), certolizumab (RR 0.57, 95% CI 0.47–0.7, NNT = 2, moderate effect magnitude), adalimumab (RR 0.68, 95% CI 0.61–0.75, NNT = 2, small effect magnitude) and ustekinumab (RR 0.73, 95% CI 0.61–0.86, NNT = 2, small effect magnitude). Two treatments were rated at low GRADE certainty and are maybe more effective at preventing loss of clinical response compared to placebo. In order of SUCRA ranking these are infliximab (RR 0.7, 95% CI 0.59–0.82, small effect magnitude), and vedolizumab (RR 0.82, 95% CI 0.68–0.99, trivial effect magnitude). One treatment, tofacitinib, was rated at low GRADE certainty as maybe being similar to placebo at preventing loss of clinical response (RR 0.9, 95% CI 0.74–1.09). The evidence for filgotinib was of very low certainty and no conclusions can be drawn (eTable 6, Figure 4, eFigure 3). The comparison-adjusted funnel plot for the assessment of small-study size was not significantly asymmetrical (eFigure 5).

### Endoscopic relapse

NMA could not be performed because this outcome was only reported in six studies and a network could not be established.^34,38–42^

### WAEs, SAEs and TAEs

Twenty-eight studies (*n* = 6155) assessing 17 interventions were included in the WAEs NMA, 23 studies (*n* = 6038) assessing 18 interventions for SAEs and 23 studies (*n* = 5157) assessing 14 interventions for TAEs (eFigure 2). Network heterogeneity was 51.2%, 52% and 60% respectively (*I*^2^).

One treatment, vedolizumab, had moderate GRADE certainty for probably no difference from placebo for WAEs (RR 0.82, 95% CI 0.52–1.29). Four treatments had low GRADE certainty for maybe no difference from placebo. In order of GRADE certainty these were: ustekinumab (RR 0.72, 95% CI 0.32–1.53), tofacitinib (RR 0.78, 95% CI 0.36–1.69), etrolizumab (RR 1.42, 95% CI 0.59–3.42) and upadacitinib (RR 1.51, 95% CI 0.6–3.8). The other treatments were rated at very low GRADE certainty of evidence, and no conclusions can be drawn about them (eTable 6, eFigures 1 and 3).

Six treatments had low GRADE certainty for maybe no difference from placebo for serious events. In order of GRADE certainty these were: adalimumab (RR 0.53, 95% CI 0.2–1.43), vedolizumab (RR 0.7, 95% CI 0.25–1.92), ustekinumab (RR 0.73, 95% CI 0.23–2.37), upadacitinib (RR 0.8, 95% CI 0.26–2.47), tofacitinib (RR 0.94, 95% CI 0.28–3.13) and etrolizumab (RR 1.00, 95% CI 0.25–3.99). The other treatments were rated at very low GRADE certainty of evidence, and no conclusions can be drawn about them (eTable 6, eFigures 1 and 3).

Five treatments had low GRADE certainty for maybe no difference from placebo in leading to TAEs. In order of GRADE certainty these were: vedolizumab (RR 0.95, 95% CI 0.72–1.26), upadacitinib (RR 1.00, 95% CI 0.73–1.38), ustekinumab (RR 0.97, 95% CI 0.7–1.33), etrolizumab (RR 0.99, 95% CI 0.72–1.36) and tofacitinib (RR 1.09, 95% CI 0.76–1.55). The other treatments were rated at very low GRADE certainty of evidence, and no conclusions can be drawn about them (eTable 6, eFigures 1 and 3).

## Discussion

In the last two decades, several advanced therapies have been approved by licensing bodies for the treatment of CD. Most phase III trials seeking regulatory approvals used to have a placebo comparator arm only. With only three head-to-head trials to date in SEAVUE, SEQUENCE and VIVID-1, it is very difficult for the practicing clinician to position these new therapies in an effective sequence backed by strong clinical evidence.^5–7^ In the absence of head-to-head RCTs, a well-executed NMA can partially address this problem, while providing sound evidence to plan future comparative studies.

We have undertaken the most extensive NMA to date on the efficacy and safety of maintenance immunomodulator and advanced therapy in CD, with 37 RCTs and 7415 participants, and a transparent GRADE assessment of the certainty of the evidence that assesses the conclusions we can make on these therapies’ efficacy and safety based on all the available RCT evidence to date. This NMA follows on from our recent NMA on induction therapies in CD.^13^ We have observed that adalimumab was the most effective treatment at maintaining clinical remission by reducing clinical relapse with moderate-certainty evidence and with a moderate magnitude of effect. We had previously defined efficacy thresholds for important and critical IBD outcomes in a multinational survey.^14^ This finding is in support of previous observations suggesting that a combination strategy of adalimumab and purine analogues had the largest effect size at the induction of clinical remission. Similarly, this supports prior observations made through the PANTS cohort of the relatively low incidence of immunogenicity observed in adalimumab-exposed patients by week 54.^43^ While no treatments had any high-certainty evidence, infliximab and CTP-13 had low-certainty evidence of a moderate and large magnitude of efficacy respectively, at reducing clinical relapse. Other therapies including methotrexate, vedolizumab, ustekinumab and upadacitinib had low certainty evidence of maybe being similar to placebo at reducing clinical relapse. The efficacy of methotrexate at maintenance of clinical remission is unclear. A previous Cochrane meta-analysis had shown that intramuscular methotrexate was superior to placebo for maintenance of remission with a 40-week follow-up, though no signal of efficacy could be drawn for 12.5–15 mg oral weekly dosing.^44^

Vedolizumab is an approved treatment for the induction and maintenance of remission in moderate to severe CD.^29,45^ In our induction NMA, we showed that vedolizumab had a trivial effect better than placebo at the induction of remission with low-certainty evidence, which would explain why its efficacy of maintenance of remission is similar to placebo, with efficacy ranging from moderately less to trivially more when compared to placebo.

Similarly, ustekinumab and upadacitinib are licensed medical therapies for the induction and maintenance of remission in moderate to severe CD.^40,46^ In this NMA, we have shown that ustekinumab may be the same as placebo at reducing clinical relapse, with low-quality evidence showing an indirect RR of 0.83 (0.64–1.08). Similar data for upadacitinib show this therapy to have low-quality evidence at reducing clinical relapse of 0.72 (0.52–1.02). Both therapies had a small effect at inducing clinical remission with moderate and low certainty respectively, in our induction NMA. In addition, the efficacies for both agents we show in this NMA are similar to those recently published for both upadacitinib 30 mg once daily (RR 0.61; 0.52–0.72) and ustekinumab 90 mg 8 weekly (RR 0.77; 0.66–0.91) or 90 mg 12 weekly (RR 0.82; 0.67–1.00).^9^

We were unable to undertake our pre-planned subgroup analysis comparing advanced therapy naïve with non-naïve advanced therapy patients. No included studies on advanced therapies were on naïve patients, as their participants were induced with or had responded to an advanced treatment. Thus, we are not able to make any conclusions on the comparison between biologic-naïve and biologic-exposed populations. Our unplanned sensitivity analysis on the effects of studies not involving advanced treatments, studies taking place in 2003 or prior, and studies of 50 participants or less did not reveal major differences from the main analysis.

In this NMA, we had made an a priori decision of excluding trials with a run-through design and focus only on those with a re-randomisation strategy at the start of maintenance therapy to ensure the cohorts we were analysing were actually in remission or had experienced a clinical response. This is a novel methodology that has never been undertaken as previous NMAs did not distinguish these two diverse trial methodologies.^47^

Maintenance of clinical response is an important clinical outcome as recently illustrated by STRIDE.^48^ Only upadacitinib was rated as high GRADE certainty as being more effective than placebo, though with a small magnitude of effect. All other therapies were of moderate certainty (in order of SUCRA ranking, CTP-13, natalizumab, certolizumab, adalimumab and ustekinumab) or low certainty (in order of SUCRA ranking, infliximab and vedolizumab). Such data is once again novel, as most other NMAs only present data pertaining to loss of clinical remission rather than clinical response, with this being an important endpoint in clinical trials with our induction NMA showing RR ranging from 2.78 to 1.86 for therapies with moderate-certainty evidence and 2.85 to 1.35 for therapies with low-certainty evidence.

Endoscopy relapse was only reported in a minority of the included studies so these data could not be synthesised in this NMA.

Medication safety was assessed through WAE, SAE and TAE. The vast majority of therapies had low-certainty or very low-certainty evidence, with all therapies showing no difference to placebo when it comes to safety-related events. Notably, the only therapy to show moderate-certainty evidence is vedolizumab, showing no difference to placebo. These findings echo the safety profile of these therapies at least in the short term. It is important to emphasise that any conclusions from our safety results need to be approached with high caution. RCT is not the ideal study design for gathering safety data, even though it is ideal for comparisons between treatments, because the data gathered is usually not enough to lead to precise results, and/or is not gathered in a universally accepted standardised manner across studies, leading to potential problems with inconsistency and bias. Large safety cohort studies are needed to combat these RCT limitations.

It is important to note that the cumulative placebo relapse rate and the cumulative placebo loss of response rate are 68% and 69%, respectively. These findings are significant and echo recent evidence synthesis investigating harm with placebo in maintenance trials of advanced therapies in inflammatory bowel disease. A previous study showed that the risks of treatment-emergent adverse events, serious infection or venous thromboembolism were not different between any active drug and placebo, the risk of infections and any-drug-related adverse events was lower in placebo, but the risks of worsening disease activity, withdrawal due to adverse events, SAEs and serious worsening of IBD activity were significantly higher with placebo.^49^ These data indicate the potential harm that patients might experience if recruited within a trial and are randomised to placebo and emphasise the point that novel trial designs randomising to best standard practice should be employed going forward.

Our analyses have several advantages over other NMAs. This study included all up-to-date studies. We gave greater importance to certainty of evidence by GRADE analysis contrary to SUCRA ranking, as was the case with previous NMAs.^47,50^ The SUCRA approach only focuses on point estimates of effect and relying on only the SUCRA approach without certainty of evidence can result in misinterpretation of the results of an NMA. Moreover, we only included studies with a re-randomisation design to ensure that all patients studied had achieved response or remission before they entered the maintenance studies and were randomly distributed in their interventions. It would not be possible to properly do a maintenance NMA by combining trials with a run-through design.

We acknowledge some limitations within this NMA. Firstly, all intervention doses were combined, hence precluding further analyses on dosing regimens. Moreover, disease activity at the beginning of the maintenance trials and length of follow-up varied in the different clinical trials, which spanned over two decades. The majority of trials had very loose inclusion criteria around eligible disease activity levels, and arbitrary follow-up duration. This precluded further analyses based on baseline disease activity and outcome measurement timepoints. Because of our focus on excluding trials with a run-through design, we excluded earlier trials that would have had a higher prevalence of bio-naïve patients. Even though we believe subgroup analyses around sex, age, concomitant steroid use and many other characteristics would also be very useful, studies offer outcome results for none of them, meaning such analyses cannot be performed. We have taken the best methodological decisions we could in a relatively flawed field and produced the most accurate results possible at this point in time with the data that is available.

## Conclusion

This maintenance NMA complements our recent induction NMA in CD, providing high-quality evidence with a particular focus on the GRADE certainty of evidence for the practicing clinician. We have shown that adalimumab has moderate certainty of evidence of moderate effect of maintaining clinical remission by reducing clinical relapse. The best therapy for the loss of clinical response was upadacitinib with high certainty of evidence of a small magnitude effect. We have observed no increased risk of SAE, TAE or WAE with any of the therapies when compared to placebo. Novel therapies seem to have similar effect sizes of efficacy while maintaining good safety records, though imprecision because of limited evidence precludes further conclusions.

## Supplemental Material

sj-docx-1-tag-10.1177_17562848261470683 – Supplemental material for Immunomodulators and advanced therapies for maintenance of remission in Crohn’s disease: systematic review and network meta-analysis Supplemental material, sj-docx-1-tag-10.1177_17562848261470683 for Immunomodulators and advanced therapies for maintenance of remission in Crohn’s disease: systematic review and network meta-analysis by Vassiliki Sinopoulou, Morris Gordon, Shiyao Liu, Daniel Arruda Navarro Albuquerque, Aderonke Ajiboye, Sudheer Kumar Vuyyuru, Shellie Jane Radford and Gordon William Moran in Therapeutic Advances in Gastroenterology

sj-docx-2-tag-10.1177_17562848261470683 – Supplemental material for Immunomodulators and advanced therapies for maintenance of remission in Crohn’s disease: systematic review and network meta-analysis Supplemental material, sj-docx-2-tag-10.1177_17562848261470683 for Immunomodulators and advanced therapies for maintenance of remission in Crohn’s disease: systematic review and network meta-analysis by Vassiliki Sinopoulou, Morris Gordon, Shiyao Liu, Daniel Arruda Navarro Albuquerque, Aderonke Ajiboye, Sudheer Kumar Vuyyuru, Shellie Jane Radford and Gordon William Moran in Therapeutic Advances in Gastroenterology

sj-pdf-1-tag-10.1177_17562848261470683 – Supplemental material for Immunomodulators and advanced therapies for maintenance of remission in Crohn’s disease: systematic review and network meta-analysis Supplemental material, sj-pdf-1-tag-10.1177_17562848261470683 for Immunomodulators and advanced therapies for maintenance of remission in Crohn’s disease: systematic review and network meta-analysis by Vassiliki Sinopoulou, Morris Gordon, Shiyao Liu, Daniel Arruda Navarro Albuquerque, Aderonke Ajiboye, Sudheer Kumar Vuyyuru, Shellie Jane Radford and Gordon William Moran in Therapeutic Advances in Gastroenterology

## Declarations

**Ethics approval and consent to participate:** This original research systematic review is based on publicly available data and there were no participants, therefore it is exempt from ethics approval.

**Consent for publication:** Not applicable.

**Availability of data and materials:** Most data are shared in the Supplemental Material. However, further data are available upon reasonable request.
