# Supplementary material for: Immunomodulators and advanced therapies for maintenance of remission in Crohn’s disease: systematic review and network meta-analysis
Source: Ther Adv Gastroenterol. 2026 Jul 27;19:17562848261470683. doi: 10.1177/17562848261470683 (PMC13408072; doi:10.1177/17562848261470683)
Supplement: sj-docx-2-tag-10.1177_17562848261470683 – Supplemental material for Immunomodulators and advanced therapies for maintenance of remission in Crohn’s disease: systematic review and network meta-analysis [file sj-docx-2-tag-10.1177_17562848261470683.docx]

**Supplementary Content**

**eTable 1**. Characteristics of included studies (page 2)

**eTable 2.** Included studies’ efficacy outcome definitions, baseline disease activity, and time of primary outcome measurement (page 28)

**eTable 3**. Outcomes reported in the included studies (page 46) **eTable 4.** Excluded studies and reasons for exclusion (page 50) **eTable 5.** Predefined Magnitude Effect Thresholds (page 51) **eTable 6.** Summary of Findings Tables and GRADE decisions (page 52)

**eFigures 1.** GORDON plots (page 76)

**eFigures 2**. Network plots (page 81)

**eFigures 3.** Network forest plots, SUCRA probabilities, and direct/indirect/network estimates forest plots (page 86)

**eFigures 4.** Subgroup and sensitivity analyses (page 101)

**eFigures 5.** Comparison adjusted funnel plots for the assessment of small study effects (page 104)

**eAppendix 1**. Search strategy (page 110)

**eAppendix 2.** References of included studies (page 113)

**eTable1. Characteristics of included studies (n=37)**

| **Study ID (Author, Year)** | **Intervention (dosage)**  **(numbers randomised)** | **Comparator (numbers randomised)** | **Sex per group (M/F), n** | **Age per group at baseline, mean (year, SD)** | **Age at diagnosis, mean (SD)** | **Disease Duration per group, mean (SD)** | **Disease location per group, n** | **Disease Phenotype** | **Previous experience with biologic therapy** | **Biologic naive or not** | **Concurrent immunosuppressants (>20% or ≤20%)** | **Concomitant steroid use, n** | **Mixed disease state or remission only** |
| --- | --- | --- | --- | --- | --- | --- | --- | --- | --- | --- | --- | --- | --- |
| **Buhl 2022 (STOP-IT)** | Azathioprine  (Unclear)  (n=56) | Infliximab  (5mg/kg intravenous every 8 weeks)  +  Azathrioprine  (Unclear) (n=59) | IG: 28/28 CG: 33/26 | IG: 32 (25–50) CG: 36 (26–50)  Median (IQR) | NR | IG: 5 (3–15) CG: 6 (3–12) years  Median (IQR) | IG: Ileal 4, Colonic 24, Ileocolonic 28 CG: Ileal 8, Colonic 17, Ileocolonic 34 | NR | Yes. All patients had been treated with standard IFX maintenance therapy for at least 1 year | Not | >20% | Concomitant systemic steroid treatment was not allowed | Remission |
| **Colombel 2007 (CHARM)- Maintenance (Responders)** | Adalimumab  (40 mg subcutaneous every other week or  40 mg subcutaneous weekly)  (n=329) | Placebo  (n=170) | All patients  188/311 | All patients  36.7 (11.6) | NR | at least 4 months’ duration | All patients  Colonic 375, Ileal 357, Gastroduodenal 30, Other 64 | NR | Previous TNF-antagonist exposure: n (%) 238 (47.7) out of 499 participants | Not | >20% | All patients  Prednisone 130, Budesonide 48 | Mixed responders and remission |
| **Feagan 2000** | Methotrexate  (15 mg intramuscularly once  weekly)  (n=40) | Placebo  (n=36) | IG: 16/40 CG:22/36 | IG: 32 (2) CG: 34 (2) | NR | IG: 88 (10) months CG: 84 (10) months | IG: Colon 11, Small bowel 18, Both 11 CG: Colon 9, Small bowel 11, Both 16 | NR | NR | Not | Part of intervention | Concomitant systemic steroid treatment was not allowed | Remission |
| **Feagan 2014** | Infliximab  (5 mg/kg intravenously at week 14, 22, 30, 38, 46)  (n=63) | Methotrexate  (25 mg subcutaneous weekly)  (n=63) | Baseline of induction  IG: 37/26 CG: 34/29 | Baseline of induction  IG: 38.5 (12.9) CG: 40.4 (13.3) | NR | Baseline of induction  IG: 115.4 (103.2) months CG: 130.9 (119.7) months | Baseline of induction  IG: Small bowel 13, Ileocolitis 35, Colitis 14, Unknown 1 CG: Small bowel 11, Ileocolitis 38, Colitis 14, Unknown 0 | NR | Patients who had been treated previously with infliximab were not eligible | Not | NR | All patients were required to discontinue prednisone. | NR |
| **Feagan 2015c (IM-UNITI)** | Ustekinumab  (90 mg subcutaneous ever 12 weeks or every 8 weeks)  (n=264) | Placebo  (n=133) | IG1: 56/76  IG2: 58/74 CG: 59/133 | IG1: 37.9 (13.2)  IG2: 28.6 (13.7) CG: 39.5 (12.7) | NR | IG1: 10.3 (8.7) years  IG2: 9.5 (8.7) years CG: 10.6 (9.5) years | IG1: Ileum 26, Colon 23, Ileum and colon 83, Proximal GI 18, Perianal GI 39  IG2: Ileum 19, Colon 29, Ileum and colon84, Proximal GI 19, Perianal GI 46 CG: Ileum 19, Colon 28, Ileum and colon 86, Proximal GI 28, Perianal GI 43 | NR | No history of TNF antagonist treatment — no. (%) CG = 52 (39.1) IG1= 53 (40.2) IG2=52 (39.4) | Not | >20% | Glucocorticoid use:  IG1: 58  IG2: 64  CG: 59 | Mixed responders and remission |
| **Ferrante 2022-FORTIFY** | Risankizumab  (180 mg or 360 mg  subcutaneous every 8 weeks)  (n=358) | Placebo  (subcutaneous every 8 weeks)  (n=184) | Baseline of induction  IG1: 68/89  IG2: 81/60 CG: 89/75 | Baseline of induction  IG1: 39.1 (14.8)  IG2: 37.0 (12.8) CG:38.0 (13.0) | NR | Baseline of induction  IG1: 10.8 (10.2) years  IG2: 9.3 (8.1) years CG: 9.6 (8.8) years | Baseline of induction  IG1: Ileal 15, Colonic 70, Ileal-colonic 72  IG2: Ileal 15, Colonic 59, Ileal-colonic 67 CG: Ileal 23, Colonic 62, Ileal-colonic 79 | NR | ADVANCE enrolled patients with demonstrated intolerance or disease with inadequate response to conventional (i.e., without previous bio-failure) or biological (i.e., with previous bio-failure) therapies, whereas MOTIVATE exclusively enrolled patients with previous bio-failure | Not | >20% | Baseline of induction  Corticosteroid use  IG1: 51  IG2:43  CG: 51 | Mixed responders and remission |
| **Hanauer 2002 (ACCENT I) - responders (maint.)** | Infliximab  (5 mg/kg or 5 mg/kg infliximab at weeks 2 and 6 followed  by 10 mg/kg thereafter intravenous every 8 weeks)  (n=225) | Placebo  (n=110) | All patients  130/205 | All patients  35 (27-46)  Median (IQR) | NR | All patients  7.5 (3.7-14.2) years  Median (range) | All patients  Ileum 74/331, Colon 74/331, Ileum and colon 183/331, Gastroduodenum 24/335 | NR | Patients were excluded from the study if they had received Infliximab in the past or any other agent targeting TNF | Not | >20% | All patients  Any concomitant corticosteroids: 172  >20 mg per day 61 | Mixed responders and remission |
| **Hanauer 2024-LIBERTY CD** | CTP13  (120 mg intravenous every 2 weeks; from week 22 lost response patients receive 240mg CTP13)  (n=231) | Placebo  (every 2 weeks; from week 22 lost response patients receive 240mg CTP13)  (n=112) | Baseline of induction  IG:134/97 CG: 69/43 | Baseline of induction  IG: 36 (25–43) CG: 29 (23–39)  Median (IQR) | NR | Baseline of induction  IG: 4.34 (5.18) years CG: 4.45 (5.78) years | NR | NR | NR | Not | NR | corticosteroids at baseline of induction  IG: 99  CG: 44 | Mixed responders and remission |
| **Jorgensen 2017 (NORSWITCH)** | Infliximab  (Unchanged from those  before randomisation)  (n=78) | CTP13  (Unchanged from those  before randomisation)  (n=77) | IG: 45/33 CG: 47/30 | IG: 38.0 (13.4) CG: 39.5 (14.2) | NR | IG: 12.8 (9.0) CG: 14.3 (8.5) | IG: Ileum 13, Colonic 25, Ileocolonic 39, Upper GI tract 7 CG: Ileum 13, Colonic 23, Ileocolonic 38, Upper GI tract 6 | IG: Non-stricturing, non-penetrating 43, Stricturing 17, Penetrating 18 CG: Non-stricturing, non-penetrating 43, Stricturing 16, Penetrating 18 | IG: 60/77 and CG: 61/78 naïve | Mix 60/77 and 61/78 naive | >20% | Prednisolone use:  IG: 2  CG: 0 | Mixed responders and remission |
| **Lemann 2005** | Azathioprine  (25 mg orally)  (n= 40) | Placebo  (25 mg orally)  (n=43) | IG: 19/21 CG: 18/22 | IG: 40 (14) CG: 36 (11) | NR | IG: 11 (6) years CG: 11 (5) years | IG: Small bower 5, Colon 19, Both 16 CG: Small bower 4, Colon 17, Both 22 | NR | Patients who had been treated previously with biological agents were not eligible | Not | Part of intervention | Steroids 10 mg/day  IG: 3  CG: 2 | Remission |
| **Loftus 2023 (U-ENDURE)** | Upadacitinib  (15 mg or 30 mg orally once daily)  (n= 337) | Placebo  (n=165) | IG1: 102/67  IG2: 93/75 CG: 88/77 | IG1: 38.1 (13.5)  IG2: 37.0 (13.3) CG: 38.1 (13.0) | NR | IG1: 7.9 (0.3-40.1) years  IG2: 7.2 (0.3-44.9) years CG: 7.6 (0.3-48.7) years  Median (range) | IG1: Ileal 22, Colinic 62, Ileal-colonic 85  IG2: Ileal 20, Colinic 70, Ileal-colonic 78 CG: Ileal 24, Colinic 67, Ileal-colonic 74 | NR | Patient who demonstrated an inadequate response or intolerance to biologic agents were included in the study | Not | <20% | Glucocorticoids use:  IG1: 63  IG2: 63  CG: 61 | Remission |
| **Louis 2023 (SPARE)** | Infliximab  (same dose as at inclusion)  +  Azathrioprine  (same dose as at inclusion)  (n=71) | Azathioprine  (same dose as at inclusion)  (n=71)  Infliximab  (same dose as at inclusion)  (n=69) | IG: 37/30 CG1: 43/28  CG2: 38/31 | IG: 63 (27-45.5) CG1: 32 (25-42.5)  CG2: 31 (26-44)  Median (IQR) | NR | IG: 6.4 (3.2-12.7) years CG1: 6.7 (3.3-10.7) years  CG2: 6.8 (2.9-12.6) years | IG: ileal 14, colonic 23, ileocolonic 30, upper gastrointestinal tract 9 CG1: ileal 10, colonic 20, ileocolonic 41, upper gastrointestinal tract 8  CG2: ileal 13, colonic 21, ileocolonic 35, upper gastrointestinal tract 5 | IG: non-stricturing, non-penetrating 42, stricturing (without fistula) 10, Penetrating (with or without stricture) 15 CG1: non-stricturing, non-penetrating 45, stricturing (without fistula) 9, Penetrating (with or without stricture) 17  CG2: non-stricturing, non-penetrating 40/68, stricturing (without fistula) 11/68, Penetrating (with or without stricture) 17/68 | Participants were patients who had achieved steroid-free clinical remission on combination therapy with infliximab and immunosuppressant therapy | Not | Part of Intervention | All participants were steroid-free | Remission |
| **Mantzaris 2009** | Budesonide 6 –9 mg a day controlled-release (n=39) | Azathioprine 2-2.5mg/kg a day (n=38) | IG: 17/22  CG: 17/21 | IG: 34.5 (19–62)  CG: 34.3 (19–59) | IG: 2.1 (0.5)  CG: 2.1 (0.6) | IG: 1.8 (0.6)  CG: 2.1 (0.5) | IG  Ileocolitis (L3B1): 26  Proximal colitis: 13  CG  Ileocolitis (L3B1): 24  Proximal colitis: 14 | NR | Patients who had received infliximab, were excluded | Not | Part of intervention | Mean (SD) time on steroids (wk.)  IG: 9.0 (1.4)  CG: 9.0 (1.2) | Remission |
| **O’ Donoghue 1978** | Azathioprine  2 mg/kg body weight/day, for at least six months  (n=24) | Placebo (n=27) | IG: 11/13 CG:11/16 | Range (Years)  IG 40 (21-78)  CG 40.5 (22-65) | NR | IG:8.25 (1.5-20.0)  CG: 7.05 (1.5-15.0) | IG:  Colon: 11  Ileo-colic: 10  Small bowel: 3  CG:  Colon:17  Ileo-colic: 3  Small bowel:7 | NR | No previous experience | Naive | Part of intervention | Other drugs  IG: 8  CG: 7 | Remission |
| **Panes 2013** | Azathioprine 2.5 mg/kg/day (adjusted to the upper multiple of 25 mg and administered in a single oral daily dose) (n=68) | Placebo (n=63) | IG: 29/39 CG:30/33 | Median (year)  IG: 35.0 CG: 36.0 | NR | NR | IG  Ileal: 31  Colonic:16  Ileocolonic: 21  CG  Ileal: 28  Colonic: 10  Ileocolonic: 45 | NR | Patients who had previously been treated with, infliximab or adalimumab were ineligible | Naive | Part of intervention | IG: 47  CG:45 | Mixed responders and remission |
| **Panes 2017 Maintenance** | IG1  Tofacitinib  5mg twice daily for 26 weeks.  (n=60)  IG 2  Tofacitinib  10mg daily for 26 weeks. (n=61) | Placebo (n=59) | IG1:30/30  IG2: 24/37 CG:26/32 | IG1: 38.1 (11.9)  IG2: 39.0 (13.1) CG: 41.5 (12.8) | NR | IG1: 11.2 (8.3)  IG2: 12.6 (10.0) CG: 12.5 (9.3) | IG1  L1 (I/TI): 6  L14 (I/TI+UGI): 3  L2 (C): 3  L24 (C+UGI):11  L3 (IC):13  L34 (IC+UGI):24  L4 (UGI):0  IG2  L1 (I/TI): 5  L14 (I/TI+UGI): 1  L2 (C): 3  L24 (C+UGI):10  L3 (IC):13  L34 (IC+UGI): 28  L4 (UGI):0  CG  L1 (I/TI): 6  L14 (I/TI+UGI): 2  L2 (C): 4  L24 (C+UGI): 9  L3 (IC): 15  L34 (IC+UGI):23  L4 (UGI): 0 | NR | Previous experience of biologic therapy as a whole (which includes JAK inhibitors) is NOT REPORTED  Previous experience of TNFi – CG 39/59 (66.1%). IG-T5 48/60 (80.0%). IG-T10 48/61 (78.7%) | Not | <20% | IG1: 48  IG2: 48  CG: 39 | Mixed responders and remission |
| **Roder 2021** | Infliximab infusion in a dose of 5mg/kg  (n=89)  Crohn’s disease: 52  Ulcerative colitis: 37 | CTP13 (n=69)  Crohn’s disease: 69  Ulcerative colitis: 42 | IG:47/42 CG:59/52 | IG: 39.9 (14.0)  CG: 37.3 (12.8) | NR | NR | NR | NR | Previous use of infliximab  7.2% (Biosimilar group)  14.6% (Originator) | Not | <20% | IG: 32 CG: 25 | Mixed responders and remission |
| **Rosenberg 1975** | Azathioprine f 4 mg. per kg. body weight for the first 10 days and 2 mg. per kg. body weight thereafter (n=10) | Placebo (n=10) | Reported for the whole group only for those who completed the study  12/6 | NR | NR | NR | Not reported per group  Ileum; anal canal: 1  Ileum: 5  Ileum; descending colon: 1  Rectum; anus: 2  Ileum; colon: 1  Jejunum: 1  Duodenum: Jejunum; ileum: 2  Ileum; total colon:1  Ileum; ascending and transverse colon: 1  Ileum; colon  and rectum:1 | IG: CG: | No previous experience | Naive | Part of intervention | Reported for both groups (3) | Unclear |
| **Rutgeerts 1999** | Infliximab  10 mg/kg (n=37)  All patients, regardless of treatment group, were to receive 4 infusions at weeks 12, 20, 28, and 36. | Placebo (n=36) | IG: 15/22 CG: 23/13 | Median (Range)  IG 34(20-64)  CG 39(20-65) |  | Median (Range)  IG: 9.4(1.1-30.8)  CG: 12.1(0.3-32.8 | IG:  Ileum and colon: 23  Colon:9  Ileum:5  CG:  Ileum and colon: 17  Colon:14  Ileum:5 | NR | No (patients were induced with infliximab in a previous trial, one of the first on infliximab) | Not | Unclear | NR | Mixed responders and remission |
| **Rutgeerts 2012 (EXTEND)** | Adalimumab subcutaneous 40mg every other week  (n=64) | Placebo (n=65) | Baseline of induction  IG:24/40 CG:24/41 | IG: 37.1 (11.1)  CG: 37.2 (12.6) | IG: CG: | IG: 10.4 (8.0) CG: 9.8 (8.4) | IG  Colon 51  Ileum 48  Rectum 19  Anal/Perianal 15  Gastrodudodeum 3  Other 3  Jejunum 0  CG  Colon 57  Ileum 41  Rectum 25  Anal/Perianal 16  Gastrodudodeum 6  Other 2  Jejunum 1 | NR | Patients undergoing previous treatment with infliximab or other anti-TNF agents (except adalimumab) were eligible unless they were a primary nonresponder | Not | >20% | IG: 9  CG: 25 | Mixed responders and remission |
| **Sandborn 2005a (ENACT 2)** | Natalizumab infusion 300 mg every four weeks  (n=168) | Placebo infusion every four weeks (n=171) | IG:77/89 CG:59/112 | IG: 37±13 CG: 37±12 | IG: CG: | Months  IG:119±95 CG:116±88 | IG:  Ileum 41  Ileum and colon 85  Colon 42  CG:  Ileum 40  Ileum and colon 85  Colon 46 | IG: CG: | IG - Y 55 (33%), Refractory to such therapy 32 (58%)  n  CG - Y 68 (40%), Refractory to such therapy 40 (23%) | Not | >20% | IG: 64  CG: 76 | Mixed responders and remission |
| **Sandborn 2007b (CLASSIC II)** | IG 1  Adalimumab subcutaneous 40mg every other week  IG 2  Adalimumab subcutaneous 40mg weekly | Placebo (n=18) | IG 1: 7/12  IG 2: 9/9 CG: 6/12 | IG1: 34 (12)  IG2: 38 (10)  CG: 36 (13) | NR | Years  IG1: 7.73 (6.5)  IG2: 9.13 (9.8)  CG: 8.24 (8.3) | NR | IG: CG: | all patients had received biologics in Classic I | Not | >20% | IG1: 8  IG2:9  CG3: 10 | Remission |
| **Sandborn 2012 (CERTIFI) - Maintenance** | Ustekinumab (n=72) | Placebo (n=73) | IG: 24/48  CG: 32/41 | IG: 39.9 ± 13.5  CG: 38.2 ± 12.4 | NR | IG: 9,41 CG:10.66 | IG  Ileum 16  Ileum and colon 30  Colon 26  Proximal gastrointestinal tract 5  CG  Ileum 22  Ileum and colon 35  Colon 15  Proximal gastrointestinal tract 11 | IG: CG: | Previous experience of biologics* – CG – 110/110 (100%). IG – 109/109 (100%)  *Although not stated explicitly the proportion of patients with previous experience, it can be safely assumed to be 100% for all groups as it is a study looking at efficacy of UST in CD refractory to TNF treatment. It is stated in the main paper ‘In this 36- week, randomized, double-blind, placebo-controlled phase 2b trial of ustekinumab (comprising 8-week induction and 28- week maintenance phases), we evaluated patients with moderate-to severe Crohn’s disease that was resistant to TNF antagonists.’ | Not | >20% | NR | Mixed responders and remission |
| **Sandborn 2013 (GEMINI II) - Maintenance** | Azathioprine  (n=153) | CG1: Vedolizumab 150mg, intravenously every 8 weeks + azathioprine  (n=154)  CG2: Vedolizumab 150mg, intravenously every 4 weeks + azathioprine  (n=154) | IG: 72/81  CG1: 68/86  CG2: 82/72 | IG: 37.3 (12)  CG1: 35.1 (12.2)  CG2: 34.9 (12.2) | NR | IG: 9.6 (8.9), years  CG1: 8.4 (7.3), years  CG2: 7.7 (6.8), years | IG: ileal 19, colonic 43, ileocolonic 91  CG1: ileal 29, colonic 27, ileocolonic 98  CG2: ileal 34, colonic 47, ileocolonic 73 | NR | Prior Crohn’s disease medications, n (%)  Prior anti-TNF use VCG 82 (54) VIG1 88 (57) VIG2 83 (54) CG 72 (49) IG3 364 (72)  Prior anti-TNF failure ≥1 failure VCG 78 (51) VIG1 82 (55) VIG2 77 (50) CG 70 (48) IG3 338 (67)  Inadequate VCG 35 (45) VIG1 37 (45) VIG2 31 (40) CG 41 (59) IG3 176 (52)  Loss of response VCG 29 (37) VIG1 35 (43) VIG2 33 (43) CG 22 (31) IG3 132 (39) Intolerance VCG 14 (18) VIG1 10 (12) VIG2 13 (17) CG 7 (10) IG3 30 (9) ≥2 failures VCG 53 (35) VIG1 46 (30) VIG2 49 (32) CG 42 (28) IG3 208 (41 | Not | >20% | Corticosteroids only:  IG: 56  CG1: 59  CG2: 58  Corticosteroids + immunosuppressives:  IG: 26  CG1: 23  CG2: 22 | Mixed responders and remission |
| **Sanborn 2023 (BERGAMOT - Maintenance)** | Etrolizumab 105mg, subcutaneous, every 4 weeks  (n=217) | Placebo (n=217) | IG: 119/98 CG: 99/118 | IG: 38.8 (12.9) CG: 37.9 (12.6) | NR | IG: 6.6 (2.9-12.1)  CG: 7.8 (2.7-13.5)  (years, median, IQR) | IG: ileum 35, colon 41, ileum and colon 141  CG: ileum 43, colon 48, ileum and colon 126 | NR | All patients went through an induction phase with etrolizumab Experience with biologics at induction baseline is mentioned in the induction tables for this study | Not | >20% | IG: 91  CG: 92 | Mixed responders and remission |
| **Sands 2004 (ACCENT II) Maintenance** | Infliximab 5mg/kg, intravenously (n=96) | Placebo, intravenously (n=99) | IG: 53/43 CG: 48/51 | IG: 37 (28-47)  CG: 36 (29-46)  (years, median, IQR) | NR | IG: 10.5 (0.2-32.2)  CG: 12.3 (0.5-31.6)  (years, median, range) | IG: ileum 18, colon 34, ileum and colon 44  CG: ileum 16, colon 30, ileum and colon 53 | NR | NR. However, exclusion criteria state that if patients had received infliximab previously, they were excluded from the study. No mention of other biologics | Not | >20% | Any:  IG: 25  CG: 30  >20mg/day:  IG: 8  CG: 8 | Mixed responders and remission |
| **Schreiber 2007 PRECISE 2)** | Certolizumab pegol 400mg, subcutaneously (n=216) | Placebo (n=212) | IG: 92/123  CG: 109/101 | IG: 38 (11)  CG: 38 (12) | NR | IG: 9 (<1-33)  CG: 7 (<1-43)  (years, mean, range) | IG: terminal ileum 48, colon 57, ileocolon 110  CG: terminal ileum 53, colon 61, ileocolon 96 | NR | Patients who had received any certolizumab pegol, who had received an anti-TNF agent or other biologic therapy within 3 months before enrolment, or who had a severe hypersensitivity reaction or no clinical response after initial dosing with an anti-TNF were excluded | Not | >20% | Glucocorticoids only:  IG: 47  CG: 44  Glucocorticoids + immunosuppressives:  IG: 28  CG: 34 | Mixed responders and remission |
| **Summers 1979 (part 2)** | Azathioprine 1mg/kg (max dose = 75mg/day) (n=54) | CG1: Placebo (n= 101)  CG2: Sulfasalazine 1/2g/15kg (max dose = 2.5g/day) (n=58)  CG3: Prednisone 1/4mg/kg (max dose = 20mg/day) (n=61) | IG: 31/23 CG1: 54/47  CG2: 31/27  CG3: 31/30 | IG: 31 (9.5) CG1: 31.6 (11.7)  CG2: 32.2 (11.1)  CG3: 32.5 (13.3) | NR | IG: 68.3 (60.9) CG1: 74.8 (69.5)  CG2: 63.8 (51.8)  CG3: 80.7 (84.8)  (months, mean, SD) | IG: colon only 7%, small bowel only 37%, small bowel and colon 56%  CG1: colon only 9%, small bowel 43%, small bowel and colon 48%  CG2: colon only 10%, small bowel 50%, small bowel and colon 40%  CG3: colon only 11%, small bowel 46%, small bowel and colon 43% | NR | No previous experience | Naive | Part of intervention | Treated with steroids within 2 weeks of randomisation:  IG: 17  CG1: 32  CG2: 19  CG3: 21 | Remission |
| **Van Assche 2012 (SWITCH)** | Infliximab 5mg/kg, intravenously, every 6-8 weeks for 56 weeks (n=37) | Adalimumab 80mg, subcutaneously at inclusion and 50mg, subcutaneously every other week for 54 weeks (n=36) | IG: 20/17 CG: 18/18 | IG: 37 (29-42) CG: 38 (27-47)  (years, median, IQR) | NR | IG: 146 (116-218) CG: 167 (76-213)  (months, unclear measurement of spread) | IG: ileal 7, colonic 11, ileocolonic 19  CG: ileal 3, colonic 7, ileocolonic 26 | NR | Patient with previous exposure to ADA, and receiving IFX doses>5 mg/kg intravenously were excluded | Not | <20% | NR | Mixed responders and remission |
| **Vermeire 2017 FITZROY - maintenance** | IG1: Filgotinib 100mg, orally, once a day for 10 weeks(n=30)  IG2: Filgotinib 200mg, orally, once a day for 10 weeks  (n=30) | CG1: placebo on induction + placebo on maintenance  (n= 15)  CG2: Filgotinib on induction + placebo on maintenance  (n=14) | From induction period:  IG: 59/71  CG: 18/26 | From induction period:  IG: 37.4 (11.6)  CG: 35.1 (11.8) | NR | From induction period:  IG: 8.8 (8.5)  CG: 6.8 (5.7) | From induction period:  IG: ileal 24, colonic 29, ileocolonic 77  CG: ileal 7, colonic 6, ileocolonic 31 | NR | NOT REPORTED  However, one of the exclusion criterion states that previous JAK inhibitor therapy was not allowed. Therefore, no patient had previous JAK inhibitors (CG – 0/15, IG1 – 0/30, IG2 – 0/31) but unable to comment on TNF inhibitors and hence on biologics as a whole | Not | >20% | From induction period:  IG: 20.7 (8.6)  CG: 21.3 (8.6) (mean daily dose in mg/day, SD) | Mixed responders and remission |
| **Vermeire 2021 (VISIBLE 2)** | Vedolizumab 108mg, subcutaneously, every 2 weeks  (n= 275) | Placebo, subcutaneously, every 2 weeks  (n= 135) | IG: 157/118 CG: 66/68 | IG: 38.2 (13.9) CG: 36.1 (12.9) | NR | IG: 9.5 (8.3)  CG: 8.2 (8.4)  (years, mean, SD) | IG: ileum 66, colon 55, ileocolonic 122, other 31  CG: ileum 21, colon 26, ileocolonic 74, other 13 | NR | Patients with previous exposure to approved or investigational anti-integrin antibodies (e.g., natalizumab, efalizumab, etrolizumab, abrilumab [AMG 181]), anti-mucosal addressin cell adhesion molecule-1 antibodies, or rituximab and vedolizumab were excluded.  Anti-TNF naïve CG: 64 [47.8%] IG: 110 [40.0%]  Prior anti-TNF use: CG: 71 [53.0%]  IG: 168 [61.1%] | Not | >20% | Corticosteroids only:  IG: 67  CG: 23  Corticosteroids + immunomodulator:  IG: 189  CG: 103 | Mixed responders and remission |
| **Vermeire 2025 – DIVERSITY maintenance** | IG1 Filgotinib 100mg (n=105)  IG 2 Filgotinib 200mg (n=118) | CG1 Placebo (filgotinib 100 mg at induction) (n=56)  CG1 Placebo (filgotinib 100 mg at induction) (n=56) | IG1: 58/46  IG2: 50/68  CG1: 31/24  CG2: 29/27 | IG1: 41 (14.8)  IG2:  39 (13.7)  CG1: 39 (13.5)  CG2: 38 (14.4) | NR | IG1: 10.3 (10.7)  IG2: 9.5 (8.8)  CG1: 10.2 (9.2)  CG2: 7.8 (5.7) | IG1: Ileal 8, Colonic 41, Ileal and colonic 55 out of 104 participants; IG2: Ileal 10, Colonic 46, Ileal and colonic 62 out of 118 participants; CG1: Ileal 10, Colonic 19, Ileal and colonic 26 out of 55 participants; CG2: Ileal 9, Colonic 21, Ileal and colonic 26 out of 56 participants | NR | All patients induced with the filgotinib at induction phase | Not | IG1/2 more than 20%  CG1/2 less than 20% | IG1 33.7%  IG2 35.6%  CG1 32.7%  CG2 33.9% | Mixed clinical remission or endoscopic response at at end of induction |
| **Volkers 2017 (SIMILAR)** | Infliximab-biosimilar 5-10mg/kg in 4 to 6 doses  (n=15) | Infliximab-biological 5-10mg/kg in 4 to 6 doses  (n=6) | All patients: 20/27 (also included patients with ulcerative colitis) | All patients: mean age of 42 years, measurement of spread not reported (also included patients with ulcerative colitis) | NR | NR | NR | NR | NR | Not | NR | NR | Remission |
| **Watanabe 2012 - Maintenance** | Adalimumab 40mg, every other week  (n= 25) | Placebo  (n= 25) | IG: 16/9  CG: 15/10 | IG: 31.6 (7.2) CG: 30.8 (10.9) | NR | IG: 9.9 (5.3)  CG: 8.2 (7.4)  (years, mean, SD) | NR | NR | Patients who used infliximab or any biological agent within 8 weeks of baseline were excluded. | Not | >20% | IG: 3  CG: 5 | Mixed responders and remission |
| **Watanabe 2020 - Maintenance** | Vedolizumab 300mg, intravenously, every 8 weeks  (n= 12) | Placebo  (n= 12) | IG: 6/6 CG: 9/3 | IG: 36.7 (16.8) CG: 35.2 (13) | NR | IG: 9 (4.9) CG: 7.5 (6.6)  (years, mean, SD) | IG: ileal 2, colonic 5, ileocolonic 5  CG: ileal 2, colonic 1, ileocolonic 9 | NR | Reported as ‘Prior anti-TNF (alpha) treatment’ [numbers (percentage)]: (i) No – CG 5/12 (41.7%). IG 4/12 (33.3%). (ii) Yes – CG 7/12 (58.3%). IG 8/12 (66.7%)  Prior anti-TNF (alpha) failure [numbers (percentage)]: (i) Overall – CG 7/12 (58.3%). IG 8/12 (66.7%) (ii) Inadequate response – CG 1/12 (8.3%). IG 3/12 (25.0%). (iii) Loss of response – CG 6/12 (50.0%). IG 5/12 (41.7%). (iv) Intolerance – CG 0/12 (0.0%). IG 0/12 (0.0%) Number of drugs of anti-TNF (alpha) failure [numbers (percentage)]: (i) None – CG 5/12 (41.7%). IG 4/12 (33.3%). (ii) One – CG 5/12 (41.7%). IG 2/12 (16.7%). (iii) Two – CG 2/12 (1 6.7 %). IG 6/12 (50.0 %)  Worst    prior treatment failure [numbers (percentage)]: (i) Prior anti-TNF (alpha) failure– CG 7/12 (58.3 %). IG 8/12 (66.7%). (ii) Prior immunomodulators failure but not anti-TNF (alpha) failure – CG 2/12 (16.7%). IG 3/12 (25.0%) | Not | >20% | Oral corticosteroids and no immunomodulators:  IG: 2  CG: 3  Oral corticosteroids + immunomodulators:  IG: 3  CG: 0 | Mixed responders and remission |
| **Willoughby 1971** | Azathioprine 2mg/kg/day + their current dose of prednisolone for at least 4 weeks after prednisolone was reduce as for the induction phase  (n= 5) | Placebo  (n= 5) | IG: 1/4 CG: 3/2 | IG: 32.8 CG: 33.2  (measurement of spread not reported) | NR | IG: 10.4 CG: 9.6  (measurement of spread not reported) | IG: small bowel 3, large bowel 0, small and large bowel 2  CG: small bowel 2, large bowel 0, small and large bowel 3 | NR | No previous experience | Naive | Part of intervention | All patients received the intervention together with their current dose of prednisolone for at least 4 weeks after prednisolone was reduce as for the induction phase | Remission |
| **Young 2024 - iBaSS** | IG: reference adalimumab (crossover to SB5)  (n=57) | CG: SB5 adalimumab biosimilar (crossover to reference adalimumab)  (n=55) | IG: 24/21  CG: 22/21 | median (IQR) years  IG: 39 (30–51)  CG: 42 (30–55) | NR | median (IQR) years  IG: 9 (4–16)  CG: 10 (4–18) | IG: Ileal 11, Colonic 9, Ileal and colonic 25, + upper GI tract 7 out of 45 participants; CG: Ileal 14, Colonic 6, Ileal and colonic 23, + upper GI tract 5 out of 43 participants | B1 (inflammatory) IG 27 (60.0), CG 16 (37.2)  B2 (stricturing) IG 11 (24.4), CG 21 (48.8)  B3 (penetrating) IG 7 (15.6), CG 6 (14.0) | IG n=14 (31.1%),  CG n=11 (25.6%) | Not | IG n=24 (53.3%)  CG n= 20 (46.5%) | NR | CD in remission or with mild disease activity (modified Harvey-Bradshaw Index (mHBI) score < 8) |

**eTable2. Included studies’ efficacy outcome definitions, baseline disease activity, and time of primary outcome measurement (n=37)**

| **Study ID (Author, Year)** | **Intervention (numbers randomised)** | **Comparator (numbers randomised)** | **Length of the intervention** | **Duration of the randomize trial** | **Follow up after the randomize trial has ended**  **(intervention)** | **Definition of clinical remission** | **Definition of clinical response** | **Definition of**  **endoscopic**  **remission** | **Baseline clinical disease activity (CDAI, HBI or IBDQ)**  **mean (SD)** | **Baseline endoscopic disease activity (Rutgeerts score, CDEIS, or SES-CD)**  **mean (SD)** | **Week when primary outcomes were measured.** |
| --- | --- | --- | --- | --- | --- | --- | --- | --- | --- | --- | --- |
| **Buhl 2022 (STOP-IT)** | Azathioprine  (Unclear)  (n=56) | Infliximab  (5mg/kg intravenous every 8 weeks)  +  Azathrioprine  (Unclear) (n=59) | 48 weeks | 48 weeks | None | CDAI < 150 | NA | SES-CD score ≤2 | CDAI  IG: 35 (14–65) CG: 46 (16–71)  Median (IQR) | SES-CD  IG: 0 (0–0) CG: 0 (0–0)  Median (IQR)  (n=99) | Week 48 |
| **Colombel 2007 (CHARM)- Maintenance (Responders)** | Adalimumab  (40 mg subcutaneous every other week or  40 mg subcutaneous weekly)  (n=329) | Placebo  (n=170) | Week 4-56 | Week 4-56  (week 0-4: induction period where patients received open-label adalimumab) | 4 weeks  (no intervention) | CDAI<150 | Decrease  in CDAI from baseline by ≥70 points | NA | All patients  CDAI 316.6 (62.5) | NA | Week 56 |
| **Feagan 2000** | Methotrexate  (15 mg intramuscularly once  weekly)  (n=40) | Placebo  (n=36) | 40 weeks | 40 weeks | None | Remission was defined as both the absence of the need for prednisone therapy and CDAI <150. | NA | NA | CDAI  IG: 94 (7) CG: 84 (7) | NA | Week 40 |
| **Feagan 2014** | Infliximab  (5 mg/kg intravenously at week 14, 22, 30, 38, 46)  +  placebo  (n=63) | Methotrexate  (25 mg subcutaneous weekly)  +  Infliximab  (5 mg/kg intravenously at week 14, 22, 30, 38, 46)  (n=63) | Week 14-50 | Week 14-50  (week 0-14: induction period where patients received infliximab or infliximab+ methotrexate) | Week 66  (Intervention unclear) | CDAI<150 | NA | NA | NR | NA | Week 50 |
| **Feagan 2015c (IM-UNITI)** | Ustekinumab  (90 mg subcutaneous ever 12 weeks or every 8 weeks)  (n=264) | Placebo  (n=133) | Week 8-44 | Week 8-44  (week 0-8: induction period where patients received ustekinumab or placebo) | None | CDAI <150 | CDAI score of ≥100 points or CDAI score <150 | NA | IG1: 320.4 (66.7)  IG2: 320.4 (66.7) CG: 313.1 (58.0) | NA | Week 44 |
| **Ferrante 2022-FORTIFY** | Risankizumab  (180 mg or 360 mg  subcutaneous every 8 weeks)  (n=358) | Placebo  (subcutaneous every 8 weeks)  (n=184) | 48 weeks | 52 weeks | None | Mean daily stool frequency ≤2·8 and not worse than baseline and mean daily abdominal pain score ≤1 and not worse than baseline status | ≥30% decrease in mean stool frequency of daily values reported for 7 days before the scheduled assessment visit or ≥30% decrease in mean daily abdominal pain score, both not worse than baseline of the induction study | NA | CDAI  IG1: 132.8 (75.8)  IG2: 137.2 (67.7) CG: 133.6 (80.6) | SES-CD  IG1: 7.9 (6.4)  IG2: 8.5 (7.3) CG: 7.6 (6.6) | Week 52 |
| **Hanauer 2002 (ACCENT I) - responders (maint.)** | Infliximab  (5 mg/kg or 5 mg/kg infliximab at weeks 2 and 6 followed  by 10 mg/kg thereafter intravenous every 8 weeks)  (n=225) | Placebo  (n=110) | Week 2-54 | Week 2-54  (Week 0-2: Patients received a  5 mg/kg intravenous infusion of infliximab at week 0 and assessed at week 2.) | None | CDAI<150 | Decrease in CDAI by 70 or more points from baseline and at least 25% reduction in the total score | NA | All participants  CDAI: 299 (264-342)  IBDQ: 129 (114-147)  Median (IQR) | NA | Week 54 |
| **Hanauer 2024-LIBERTY CD** | CTP13  (120 mg intravenous every 2 weeks; from week 22 lost response patients receive 240mg CTP13)  (n=231) | Placebo  (every 2 weeks; from week 22 lost response patients receive 240mg CTP13)  (n=112) | Week 10-54 | Week 10-54  (Week 0-10: induction period where patients received infusions of CT-P13 5 mg/kg at weeks 0, 2, and 6.) | Week 56-102  (patients who might have benefited from continued treatment received CT-P13 SC via prefilled syringe) | CDAI<150 | NA | absolute SES-CD of ≤4 with a ≥2-point reduction from baseline, with no subscore >1 | CDAI  IG: 174 (75.3)  CG: 91 (81.3) | NA | Week 54 |
| **Jorgensen 2017 (NORSWITCH)** | Infliximab  (Unchanged from those before randomisation)  (n=78) | CTP13  (Unchanged from those  before randomisation)  (n=77) | 52 weeks | 52 weeks | Week 52-78  (patients were all given open-label CT-P13 treatment with an unchanged regimen in terms of dose and infusion intervals) | HBI≤4 points, PMS≤2 points | NR | NR | IG: CG: | IG: CG: | Week 52 |
| **Lemann 2005** | Azathioprine  (25 mg orally)  (n= 40) | Placebo  (25 mg orally)  (n=43) | 18 months | 18 months | None | CDAI <150 | NA | NA | CDAI  IG: 41 (43) CG: 39 (47) | CDEIS  IG: 2.5 (2.8) n=24 CG: 2.4 (3.5) n=21 | 18 months |
| **Loftus 2023 (U-ENDURE)** | Upadacitinib  (15 mg or 30 mg orally once daily)  (n= 337) | Placebo  (n=165) | 52 weeks | 52 weeks | None | CDAI<150 | Decrease of CDAI ≥100 points from baseline. | an SES-CD of ≤4, a decrease of ≥2 points from baseline, and no sub-score >1 in any individual variable | CDAI  IG1: 300.8 (90.8), n=168  IG2: 312.1 (75.4), n=168 CG: 308.4 (82.3), n=164 | SES-CD  IG1: 15.8 (7.6)  IG2: 15.5 (8.1) CG: 14.8 (7.7) | Week 52 |
| **Louis 2023 (SPARE)** | Infliximab  (same dose as at inclusion)  +  Azathrioprine  (same dose as at inclusion)  (n=71) | Azathioprine  (same dose as at inclusion)  (n=71)  Infliximab  (same dose as at inclusion)  (n=69) | 104 weeks | 104 weeks | None | CDAI <150 | NR | NR | IG: CG: | IG: CG: | Week 104 |
| **Mantzaris 2009** | Budesonide 6 –9 mg a day controlled-release  (n=39) | Azathioprine 2–2.5 mg/kg per day (n=38) | 12 months | 12 months | 6 months Patients who were still in remission at the end of the study entered a 6-month study extension to assess solely maintenance of remission on AZA or BUD. | CDAI <150 | NR | NR | IG: CG: | IG: 7.10 +3.50  CG:7.20 + 3.10 | 12 months |
| **O’ Donoghue 1978** | Azathioprine  2 mg/kg body weight/day  (n=24) | Placebo (n=27) | 6months | 12 months | None | Relapse was defined as "significant deterioration  in clinical state requiring a change in treatment as judged by  two doctors unaware of the patient's treatment" | NR | NR | Mean pre-trial disease-activity  IG: 2.33(0-9) CG: 2.0(0-7) | NR | 12 months |
| **Panes 2013** | Azathioprine 2.5 mg/kg/day (adjusted to the upper multiple of 25 mg and administered in a single oral daily dose)  (n=68) | Placebo (n=63) | 18months | 18months | None | CDAI score < 150 | NR | NR | IG: 91.3 (74.6)  CG: 114.5 (73.6) | NR | 18 months |
| **Panes 2017 Maintenance** | IG1  Tofacitinib 5 mg twice daily (n= 60)  IG2  Tofacitinib 10 mg twice daily  (n=61) | Placebo (n=59) | 26 weeks | 26 weeks | None | CDAI < 150 points | CDAI decrease from baseline ≥100 points | NR | IG 1: 131 (60.51)  IG 2: 129 (61.34)  CG: 140 (74.16 | NR | Week 26 |
| **Roder 2021**  **(Reported for Crohn’s Disease and Ulcerative Colitis)** | Infliximab infusion in a dose of 5mg/kg  (n=89)  Crohn’s disease: 52  Ulcerative colitis: 37 | CTP13 (n=111)  Crohn’s disease: 69  Ulcerative colitis: 42 | 52weeks | 52weeks | None | CDAI<150, CAI<4 | NR | NR | IG: 61.5 (79.2) CG: 68.6 (69.3) | NR | Week 52 |
| **Rosenberg 1975** | Azathioprine (n=10) | Placebo (n=10) | 2 months | 2 months | None | 3 or fewer bowel movements a day,  absent to mild pain and malaise, weight loss less than 2 kg, and  fever (> 37.5ºC) less than one fourth of the time | NR | NR | NR | NR | Week 26 |
| **Rutgeerts 1999** | Infliximab 10 mg/kg every 8 weeks  (n=37)  All patients, regardless of treatment group, were to receive 4 infusions at weeks 12, 20, 28, and 36. | Placebo (n=36) | 36 weeks | 48 weeks | None | CDAI score< 150 | > 70-point decrease in the CDAI | NR | Median  IG: 160.4  CG:179.3 | NR | Week 44 |
| **Rutgeerts 2012 (EXTEND)** | Adalimumab  subcutaneous 40mg every other week  (n=64) | Placebo (n=65) | 52 weeks | 52 weeks | None | CDAI <150 | Decrease in CDAI of at least 70 points from baseline. | CDEIS score of ≤4 | IG: 318.7 (68.6)  CG: 321.1 (72.1) | IG: 9.8 (7.4)  CG:10.9 (7.2) | Week 52 |
| **Sandborn 2005a (ENACT 2)** | Natalizumab infusion 300mg every 4 weeks (n=214) | Placebo infusion every four weeks (n=214) | 56 weeks | 60weeks | None | CDAI < 150 | Decrease in CDAI score of at least 70 points | NR | IG: 118±57  CG: 105±54 | NR | Week 36 |
| **Sandborn 2007b (CLASSIC II)** | IG1  Adalimumab 40mg subcutaneously  every other week (n=19)  IG 2  Adalimumab 40 mg weekly(n=18) | Placebo (n=18) | 56weeks | 56weeks | None | CDAI<150 | Reduction of >70 points or >100 points in the CDAI score | NR | IG: CG: | NR | Week 56 |
| **Sandborn 2012 (CERTIFI) - Maintenance** | Ustekinumab 90mg subcutaneously at weeks 8 and 16 (n=72) | Subcutaneous Placebo at weeks 8 and 16 (n=73) | Given at weeks 8 and 16 | 36weeks | None | CDAI < 150 | Decrease in CDAI by ≥100 points. | NR | IG: 332.3 ± 69.6  CG: 327.8 ± 63.0 | NR | Week 22 |
| **Sandborn 2013 (GEMINI II) - Maintenance** | Azathioprine  (n=153) | CG1: Vedolizumab 150mg, intravenously every 8 weeks + azathioprine  (n=154)  CG2: Vedolizumab 150mg, intravenously every 4 weeks + azathioprine  (n=154) | 52 weeks | 52 weeks | None | CDAI < 150 | Decrease in CDAI by ≥100 points. | NR | CDAI score:  IG: 325 (66) CG1: 326 (69)  CG2: 317 (66) | NR | Week 52 |
| **Sanborn 2023 (BERGAMOT - Maintenance)** | Etrolizumab 105mg, subcutaneous, every 4 weeks  (n=217) | Placebo (n=217) | 52 weeks | 66 weeks | None | CDAI < 150 | Decrease of at least 70 points on the CDAI | SES-CD ≤4 [≤2 for patients with ileal Crohn’s disease only] with no segment having a subcategory score of >1 | CDAI score:  IG: 322.3 (58.4) CG: 327.3 (65.2) | SES-CD:  IG: 13.63 (6.87) CG: 13.02 (7.03) | Week 66 |
| **Sands 2004 (ACCENT II) Maintenance** | Infliximab 5mg/kg, intravenously (n=96) | Placebo, intravenously (n=99) | One infusion at weeks 14, 22, 30 and 38 | 54 weeks | None | NR | A reduction of at least 50 percent from base line in the number of draining fistulas at consecutive visits four or more weeks apart | NR | CDAI score:  ≥ 150:  IG: 57 CG: 57  ≥ 220:  IG: 33  CG: 31  IBDQ score (median, IQR):  IG: 155 (135-187)  CG: 168 (145-193) | NR | Week 54 |
| **Schreiber 2007 PRECISE 2)** | Certolizumab pegol 400mg, subcutaneously (n=216) | Placebo (n=212) | One injection at weeks 8, 12, 16, 20, and 24 | 26 weeks | None | CDAI ≤150 | A reduction of at least 100 from the baseline score on the CDAI | NR | CDAI score:  IG: 306 (61) CG: 301 (62) | NR | Week 26 |
| **Summers 1979 (part 2)** | Azathioprine 1mg/kg (max dose = 75mg/day) (n=54) | CG1: Placebo (n= 101)  CG2: Sulfasalazine 1/2g/15kg (max dose = 2.5g/day) (n=58)  CG3: Prednisone 1/4mg/kg (max dose = 20mg/day) (n=61) | Unclear | 2 years | None | CDAI < 150 | NR | NR | IG 85.9 (42.6): CG1: 83.5 (42.6)  CG2: 89.4 (38.9)  CG3: 94.8 (43.9) | NR | 24 months |
| **Van Assche 2012 (SWITCH)** | Infliximab 5mg/kg, intravenously, every 6-8 weeks for 56 weeks (n=37) | Adalimumab 80mg, subcutaneously at inclusion and 50mg, subcutaneously every other week for 54 weeks (n=36) | IG: 56 weeks  CG: 54 weeks | 56 weeks | None | Complete response was defined by physician global assessment of signs and symptoms, but the (CDAI) at baseline had to be <200. | NR | NR | CDAI score:  IG: 58 (34-122) CG: 48 (24-110)  (median, IQR) | NR | Week 54 |
| **Vermeire 2017 FITZROY - maintenance** | IG1: Filgotinib 100mg, orally, once a day for 10 weeks(n=30)  IG2: Filgotinib 200mg, orally, once a day for 10 weeks  (n=30) | CG1: placebo on induction + placebo on maintenance  (n= 15)  CG2: Filgotinib on induction + placebo on maintenance  (n=14) | 10 weeks | 20 weeks | None | CDAI < 150 | Decrease in CDAI by ≥100 points. | SES-CD ≤4 and ulcerated surface sub score ≤ 1in all five segments | NR | NR | Week 10 |
| **Vermeire 2021 (VISIBLE 2)** | Vedolizumab 108mg, subcutaneously, every 2 weeks  (n= 275) | Placebo, subcutaneously, every 2 weeks  (n= 135) | 44 weeks | 68 weeks | Patients who discontinued the study due to lack  of efficacy and showed disease worsening on or after week 6, or  those who received rescue medication beyond week 14, were eligible  to enter an open-label extension [OLE; NCT02620046] study  to receive vedolizumab subcutaneously after completion of the week 52/early  termination trial assessments. These patients were also eligible for  dose escalation in the OLE study from every 2 weeks to weekly dosing of  vedolizumab subcutaneous. Patients who withdrew from the study and did not  participate in OLE were managed outside the study. | CDAI ≤150 | ≥100-point decrease in CDAI from baseline] | NR | CDAI score:  IG: 150.5 (-8 to 362) CG: 147.5 (-3 to 326)  (median, range) | NR | Week 52 |
| **Vermeire 2025 – DIVERSITY maintenance** | IG1 Filgotinib 100mg (n=105)  IG 2 Filgotinib 200mg (n=118) | CG1 Placebo (filgotinib 100 mg at induction) (n=56)  CG1 Placebo (filgotinib 100 mg at induction) (n=56) | 47 weeks (week 11-58) | 47 weeks (week 11-58) | None | Clinical remission defined as a CDAI score of <150 points  and  PRO2 clinical remission was defined as having abdominal pain subscore of not more than 1 (on a scale of 0–3) and liquid or very soft stool (Bristol stool scale type 6 or 7) frequency subscore of not more than 3 (each PRO2 subscore was calculated as the mean of the corresponding eDiary data for 7 days) | A reduction in the CDAI score from induction baseline of ≥100 points or a CDAI score of <150 | NR | NR | NR | Week 58 |
| **Volkers 2017 (SIMILAR)** | Infliximab-biosimilar 5-10mg/kg in 4 to 6 doses  (n=15) | Infliximab-biological 5-10mg/kg in 4 to 6 doses  (n=6) | 30 weeks | NR | None | HBI<5 and MAYO<5 | NR | NR | NR | NR | NR |
| **Watanabe 2012 - Maintenance** | Adalimumab 40mg, every other week  (n= 25) | Placebo  (n= 25) | 52 weeks | 52 weeks | None | CDAI<150 | CDAI decrease of ≥100 or ≥70 from baseline. | NR | CDAI score:  IG: 325.5 (62.3) CG: 296.7 (65.3)  IBDQ score:  IG: 3.1 (1.2)  CG: 3.2 (1.8) | NR | Week 52 |
| **Watanabe 2020 - Maintenance** | Vedolizumab 300mg, intravenously, every 8 weeks  (n= 12) | Placebo  (n= 12) | 46 weeks | 54 weeks | Patients could enter an open-label cohort and receive  vedolizumab 300 mg re-induction (at weeks 0, 2, and 6 from the initiation of the cohort), then every 8 weeks up to 94 weeks, if they had no CDAI-70 response at week 10,  had disease worsening (a ≥ 100-point increase in CDAI  score from week 10 value and CDAI score of ≥ 220 on  two consecutive visits during the maintenance phase),  received rescue treatment during the maintenance phase, or  completed 60 weeks of the maintenance phase. | CDAI<150 | Decrease in CDAI by ≥100 points. | NR | CDAI score:  IG: 147.9 (89.2) CG: 149.7 (59.9) | NR | Week 60 |
| **Willoughby 1971** | Azathioprine 2mg/kg/day + their current dose of prednisolone for at least 4 weeks after prednisolone was reduce as for the induction phase  (n= 5) | Placebo  (n= 5) | At least 4 weeks | 24 weeks | None | Clinical assessment proposed and by disease activity index (by modification of that proposed by Dyer et al,) | NR | NR | Disease activity score (modified from the score proposed by Dyer):  IG: 3.2 CG: 1.8  (mean, measurement of spread not reported) | NR | Week 24 |
| **Young 2024 - iBaSS** | IG: reference adalimumab (crossover to SB5)  (n=57) | CG: SB5 adalimumab biosimilar (crossover to reference adalimumab)  (n=55) | 48 weeks | 24 weeks (pre-crossover) | Crossover between weeks 24-48 | Treatment failure was defined as an increase in mHBI score of ≥ 3 (corresponding to a 100-point change in Crohn’s Disease Activity Index score) and/ or a decline in IBD-Control score of ≥ 4 points compared to baseline. | NR | NR | mHBI score, median (IQR) IG: 1 (1–4)  CG: 2 (0–4) | NR | Week 24 |

**eTable 3**. **Outcome data reported in the included studies (n=37)**

| **Study ID (Author, Year)** | **Clinical relapse** | **Loss of clinical response** | **Endoscopic relapse** | **Withdrawals due to adverse events** | **Serious adverse events** | **Total adverse events** |
| --- | --- | --- | --- | --- | --- | --- |
| **Buhl 2022 (STOP-IT)** | Aza: 31/56  InfAza: 5/59 | NR | Aza: 42/56  InfAza: 17/59 | Aza: 9/56  InfAza: 5/59 | NR | NR |
| **Colombel 2007 (CHARM)- Maintenance (Responders)** | Pl: 150/170  Ada: 202/329 | Pl:  142/170 (CDAI70)  140/170 (CDAI100)  Ada:  183/329 (CDAI70)  178/329 (CDAI100) | NR | NR | NR | NR |
| **Feagan 2000** | Pl: 22/36  Mtx: 14/40 | NR | NR | NR | NR | NR |
| **Feagan 2014** | Inf: 27/63  Mtxinf: 28/63 | NR | NR | NR | NR | NR |
| **Feagan 2015c (IM-UNITI)** | Pl: 86/133  Ust: 133/264 | Pl: 75/133  Ust: 113/264 | NR | Pl:13/133  Ust: 23/264 | Pl: 20/133  Ust: 29/264 | NR |
| **Ferrante 2022-FORTIFY** | Pl: 112/184  Ris: 202/358 | NR | Pl: 163/184  Ris: 256/358 | Pl: 10/184  Ris: 17/358 | Pl: 23/184  Ris: 46/358 | Pl: 135/184  Ris: 257/358 |
| **Hanauer 2002 (ACCENT I) - responders (maint.)** | Pl: 87/110  Inf: 131/225 | NR | NR | NR | NR | NR |
| **Hanauer 2024-LIBERTY CD** | Pl: 87/112  Ctp13: 76/213 | Pl: 79/112  CTP13: 69/213 | Pl: 100/112  Ctp13: 151/213 | NR | Pl: 8/105  CTP13: 16/238 | Pl: 8/105  CTP13: 16/238 |
| **Jorgensen 2017 (NORSWITCH)** | Inf: 37/78  ctp13: 31/77 | Inf: 38/78  CTP13: 25/77 | NR | Inf: 2/78  CTP13: 1/77 | Inf: 8/78  CTP13: 8/77 | Inf: 57/78  CTP13: 49/77 |
| **Lemann 2005** | Pl: 10/43  Aza: 3/40 | NR | NR | Pl: 1/43  Aza: 1/40 | Pl: 43/43  Aza: 39/40 | Pl: 1/43  Aza: 2/40 |
| **Loftus 2023 (U-ENDURE)** | Pl: 140/165  Upa: 207/337 | Pl: 140/165  Upa: 182/337 | Pl: 157/165  Upa: 256/337 | Pl:12/165  Upa:37/337 | Pl: 31/223  Upa: 50/450 | Pl: 169/223  Upa: 341/450 |
| **Louis 2023 (SPARE)** | Infaza: 12/71  Aza: 25/71  Inf: 6/69 | NR | NR | InfAza: 14/71  Aza: 5/71  Inf: 8/69 | InfAza: 10/71  Aza: 8/71  Inf: 13/69 | NR |
| **Mantzaris 2009** | Bud: 21/39  Aza: 9/38 | NR | NR | Bud: 0/39  Aza: 2/38 | Bud: 0/39  Aza: 2/38 | NR |
| **O’ Donoghue 1978** | Pl: 19/27  Aza: 11/24 | NR | NR | Pl: 1/27  Aza: 1/24 | Pl: 0/27  Aza: 1/24 | Pl: 0/27  Aza: 1/24 |
| **Panes 2013** | Pl: 27/63  Aza:22/68 | NR | NR | NR | Pl: 7/63  Aza:14/68 | Pl: 40/53  Aza: 58/68 |
| **Panes 2017 Maintenance** | Pl: 47/59  Tof:87/121 | Pl: 44/59  Tof: 81/121 | NR | Pl: 28/59  Tof: 45/121 | Pl: 13/59  Tof: 25/121 | Pl: 44/59  Tof: 98/121 |
| **Roder 2021** | Inf: 9/52  Ctp13: 22/69 | NR | NR | Inf: 4/52  Ctp13: 9/69 | NR | Inf: 24/52  Ctp13: 19/69 |
| **Rosenberg 1975** | Pl: 6/10  Aza: 3/10 | NR | NR | Pl: 0/10  Aza: 1/10 | NR | Pl: 0/10  Aza: 1/10 |
| **Rutgeerts 1999** | Pl: 23/36  Inf:15/37 | Pl: 23/36  Inf: 14/37 | NR | Pl: 12/36  Inf: 10/37 | NR | Pl: 35/36  Inf: 35/37 |
| **Rutgeerts 2012 (EXTEND)** | Pl: 59/65  Ada:43/64 | NR | Pl: 63/65  Ada:46/64 | Pl: 4/65  Ada: 8/64 | Pl: 5/65  Ada: 4/64 | Pl: 55/65  Ada: 61/64 |
| **Sandborn 2005a (ENACT 2)** | Pl: 89/214  Nat: 73/214 | Pl:122/214  Nat: 66/214 | NR | Pl: 61/214  Nat: 29/214 | Pl: 21/214  Nat: 18/214 | Pl: 207/214  Nat: 194/214 |
| **Sandborn 2007b (CLASSIC II)** | Pl: 10/18  Ada: 7/37 | Pl:  7/18 (CDAI70)  5/18 (CDAI100)  Ada:  12/37 CDAI70)  6/37 (CDAI100) | NR | Pl: 2/18  Ada: 3/37 | Pl: 2/18  Ada: 3/37 | Pl: 18/18  Ada: 29/37 |
| **Sandborn 2012 (CERTIFI) - Maintenance** | Pl: 57/73  Ust: 50/72 | Pl: 49/73  Ust: 32/72 | NR | Pl: 10/73  Ust: 5/72 | NR | NR |
| **Sandborn 2013 (GEMINI II) - Maintenance** | Aza: 120/153  VedAza: 192/308 | Aza: 46/153  VedAza: 137/308 | NR | Aza: 81/153  VedAza: 133/308 | Aza: 23/153  VedAza: 53/308 | NR |
| **Sanborn 2023 (BERGAMOT - Maintenance)** | Pl: 151/217  Etro: 134/217 | NR | Pl: 204/217  Etro: 191/217 | Pl: 19/217  Etro: 27/217 | Pl: 9/217  Etro: 9/217 | Pl: 190/217  Etro: 189/217 |
| **Sands 2004 (ACCENT II) Maintenance** | Pl: 79/99  Inf: 57/96 | Pl: 79/99  Inf: 57/96 | NR | Pl: 12/99  Inf: 5/96 | NR | NR |
| **Schreiber 2007 PRECISE 2)** | Pl: 152/212  Czm: 113/216 | Pl: 140/212  Czm: 82/216 | NR | Pl: 111/212  Czm: 66/216 | Pl: 14/212  Czm: 12/216 | Pl: 143/212  Czm: 140/216 |
| **Summers 1979-Part 2** | Pl: 34/101  Aza: 17/54  5-ASA: 22/58  Corticosteroids: NR | NR | NR | Pl: 1/178  Aza: 14/113  5-ASA: 10/132  Corticosteroids: NR | Pl: 2/178  Aza: 12/95  5-ASA: 0/95  Corticosteroids: NR | NR |
| **Van Assche 2012 (SWITCH)** | NR | Inf: 7/37  Ada:10/36 | NR | Inf: 1/37  Ada:10/36 | Inf: 5/37  Ada:0/36 | Inf: 30/37  Ada: 27/36 |
| **Vermeire 2017 FITZROY - maintenance** | NR | NR | NR | NR | NR | NR |
| **Vermeire 2021 (VISIBLE 2)** | Pl: 88/135  Ved: 143/275 | Pl: 75/135  Ved: 132/275 | NR | Pl: 56/135  Ved: 106/275 | Pl: 14/135  Ved: 23/275 | Pl: 102/135  Ved: 202/275 |
| **Vermeire 2025 – DIVERSITY maintenance** | FIL100 82/105  FIL200 70/118  Placebo 85/112 | FIL100 72/105  FIL200 67/118  Placebo 78/112 | NR | FIL100 17/105  FIL200 18/118  Placebo 7/112 | FIL100 14/104  FIL200 13/118  Placebo 8/111 | FIL100 75/104  FIL200 80/118  Placebo 71/111 |
| **Volkers 2017 (SIMILAR)** | NR | NR | NR | NR | NR | NR |
| **Watanabe 2012 - Maintenance** | Pl: 23/25  Ada:16/25 | Pl:  23/25 (CDAI70)  23/25(CDAI100)  Ada:  16/25 (CDAI70)  14/25 (CDAI100) | NR | Pl: 23/25  Ada:15/25 | Pl: 6/25  Ada:2/25 | Pl: 23/25  Ada:25/25 |
| **Watanabe 2020 - Maintenance** | Pl: 10/12  Ved: 7/12 | Pl: 11/12  Ved: 5/12 | NR | Pl: 8/12  Ved: 5/12 | Pl: 4/12  Ved: 2/12 | Pl: 10/12  Ved: 9/12 |
| **Willoughby 1971** | Pl: 3/5  Aza: 1/5 | NR | NR | Pl: 0/11  Aza: 0/11 | NR | Pl: 0/11  Aza: 3/11 |
| **Young 2024 - iBaSS** | NR (crossover study without differentiation between phase 1 and 2 results) | NR (crossover study without differentiation between phase 1 and 2 results) | NR (crossover study without differentiation between phase 1 and 2 results) | NR (crossover study without differentiation between phase 1 and 2 results) | NR (crossover study without differentiation between phase 1 and 2 results) | NR (crossover study without differentiation between phase 1 and 2 results) |

**eTable 4.** Excluded studies and reasons for exclusion (n=10).

| **Candy 1995** | No re-randomised maintenance phase |
| --- | --- |
| **Cosnes 2013** | No re-randomised maintenance phase |
| **D'Haens 2022 -SERENE** | No control group (compared two dosages of adalimumab) |
| **Mantzaris 2004** | No re-randomised maintenance phase |
| **Mate-Jimenez 2000** | No re-randomised maintenance phase |
| **Oren 1997** | No re-randomised maintenance phase |
| **Sandborn 2010 (WELCOME)** | No control group (compared two dosages of certolizumab) |
| **Sandborn 2020 – (CELEST)** | No control group (compared three dosages of upadacitinib) |
| **Schreiber 2021** | No control group (compared two administration routes of CT-P13) |
| **Schroder 2006** | No re-randomised maintenance phase |

**eTable 5.** Predefined Magnitude Effect Thresholds

|  | **Trivial to Small** | **Small to Moderate** | **Moderate to Large** |
| --- | --- | --- | --- |
| **Clinical Remission** | 10% | 20% | 31% |
|  |  |  |  |
| **Clinical Response** | 13% | 23% | 35% |
|  |  |  |  |
| **Endoscopic relapse** | 9% | 17% | 28% |
|  |  |  |  |
| **Withdrawals due to adverse events** | 7% | 14% | 23% |
|  |  |  |  |
| **Serious Adverse Events** | 6% | 11% | 17% |
|  |  |  |  |
| **Total Adverse Events** | 9% | 16% | 24% |
|  |  |  |  |

**eTable 6. SUMMARY OF FINDINGS TABLES AND GRADE DECISIONS**

**Summary of Findings Tables and GRADE decisions (Clinical Relapse)**

| **Clinical relapse** | | | | | | | |
| --- | --- | --- | --- | --- | --- | --- | --- |
| Patient or population: people with Crohn's disease in remission or response to treatment prior to randomisation, not biologically naïve | | | | | | | |
| Settings: hospital setting | | | | | | | |
| Intervention: advanced therapies/purine analogues/methotrexate | | | | | | | |
| Comparison: placebo | | | | | | | |
| **Treatment** | **Network evidence** | | **Anticipated absolute effects for network estimate** | | | **NNT (95% CI)** | **Notes** |
|  | **RR** | **Certainty** | **Risk with Placebo^a^** | **Risk with Agent^b^ (95% CI)** | **% Risk Difference with Agent^c^  (95% CI)** |  |  |
|  | **(95% CI)** |  |  |  |  |  |  |
| Inflximab with purine analogues | 0.30 (0.17 to 0.56) | Very low | 681 per 1,000 | 209 per,1000 (115 to 378) | 47.3% less (56.7% less to 30.1% less) | NA | The data is very uncertain |
|  |  | ⊕⊖⊖⊖ |  |  |  |  |  |
|  |  |  |  |  |  |  |  |
| CT-P13 (ifx biosimilar) | 0.52 (0.38 to 0.71) | Low | 681 per 1,000 | 365 per 1,000 (270 to 493) | 32.7% less (42.1% less to 19.9% less) | NA | Maybe large effect better than placebo (moderate to large) |
|  |  | ⊕⊕⊖⊖ |  |  |  |  |  |
|  |  |  |  |  |  |  |  |
| Infliximab | 0.61 (0.49 to 0.76) | Low | 681 per 1,000 | 419 per 1,000 (338 to 513) | 26.8% less ( 34.9% less to 16.6% less) | NA | Maybe moderate effect better than placebo (small to large) |
|  |  | ⊕⊕⊖⊖ |  |  |  |  |  |
|  |  |  |  |  |  |  |  |
| Methotrexate | 0.57 (0.31 to 1.05) | Low | 681 per 1,000 | 385 per,1000 (216 to 695) | 29.1% less (46.7% less to 3.2% more) | NA | Maybe the same as placebo. Effect ranging from largely less than placebo to trivial effect more than placebo. |
|  |  |  |  |  |  |  |  |
|  |  | ⊕⊕⊖⊖ |  |  |  |  |  |
| Methotrexate with infliximab | 0.63 (0.36 to 1.11) | Very Low | 681 per 1,000 | 432 per,1000 (250 to 756) | 24.3% less (43.8% less to 7.5% more) | NA | The data is very uncertain |
|  |  | ⊕⊖⊖⊖ |  |  |  |  |  |
|  |  |  |  |  |  |  |  |
| VedolizumabAzathioprine | 0.64 (0.40 to 1.03) | Very Low | 681 per 1,000 | 439 per,1000 (284 to 695) | 24.3% less (40.6% less to 1.8% more) | NA | The data is very uncertain |
|  |  | ⊕⊖⊖⊖ |  |  |  |  |  |
|  |  |  |  |  |  |  |  |
| Adalimumab | 0.68 (0.54 to 0.84) | Moderate | 681 per 1,000 | 459 per,1000 (371 to 567) | 22% less (31.2% less to 10.6% less) | 2 (2 to 3) | Probably moderate effect better than placebo (small to large) |
|  |  |  |  |  |  |  |  |
|  |  | ⊕⊕⊕⊖ |  |  |  |  |  |
| Upadacitinib | 0.72 (0.51 to 1.04) | Low | 681 per 1,000 | 486 per,1000 (351 to 689) | 18.8% less (33.7% less to 2.6% more) | NA | Maybe the same as placebo with an effect ranging from trivially more to largely less |
|  |  |  |  |  |  |  |  |
|  |  | ⊕⊕⊖⊖ |  |  |  |  |  |
| Certolizumab | 0.73 (0.5 to 1.06) | Very low | 681 per 1,000 | 493 per,1000 (344 to 702) | 18.4% less (33.9% less to 4.3% more) | NA | The data is very uncertain |
|  |  | ⊕⊖⊖⊖ |  |  |  |  |  |
|  |  |  |  |  |  |  |  |
| Vedolizumab | 0.77 (0.55 to 1.07) | Low | 681 per 1,000 | 520 per,1000 (378 to 716) | 15.6% less (30.3% less to 4.8% more) | NA | Maybe the same as placebo. Effect ranging from moderately less to trivially more. |
|  |  |  |  |  |  |  |  |
|  |  | ⊕⊕⊖⊖ |  |  |  |  |  |
| Ustekinumab | 0.83 (0.63 to 1.1) | Low | 681 per 1,000 | 560 per,1000 (432 to 729) | 11.5% less (25.1% less to 6.5% more) | NA | Maybe the same as placebo. Effect ranging from moderately less to trivially more. |
|  |  |  |  |  |  |  |  |
|  |  | ⊕⊕⊖⊖ |  |  |  |  |  |
| Purine analogues | 0.81 (0.6 to 1.08) | Very low | 681 per 1,000 | 554 per,1000 (419 to 743) | 13% less (27% less to 5.8% more) | NA | The data is very uncertain |
|  |  | ⊕⊖⊖⊖ |  |  |  |  |  |
|  |  |  |  |  |  |  |  |
| Natalizumab | 0.82 (0.54 to 1.25) | Very low | 681 per 1,000 | 554 per,1000 (371 to 830) | 12.2% less (31.4% less to 17.1% more) | NA | The data is very uncertain |
|  |  | ⊕⊖⊖⊖ |  |  |  |  |  |
|  |  |  |  |  |  |  |  |
| Etrolizumab | 0.89 (0.61 to 1.28) | Low | 681 per 1,000 | 601 per 1,000 (425 to 851) | 7.7% less (26.3% less to 19.4% more) | NA | Maybe the same as placebo. Effect ranging from moderately less to moderately more |
|  |  | ⊕⊕⊖⊖ |  |  |  |  |  |
|  |  |  |  |  |  |  |  |
| Filgotinib | 0.90 (0.62 to 1.30 | Very low | 681 per 1,000 | 613 per 1,000 (415 to 885) | 6.9% less  (25.8% less to 20.5% more) | NA | The data is very uncertain |
|  |  | ⊕⊖⊖⊖ |  |  |  |  |  |
| Tofacitinib | 0.9 (0.61 to 1.32) | Low | 681 per 1,000 | 608 per,1000 (425 to 878) | 6.6% less (26.2% less to 22.1% more) | NA | Maybe the same as placebo. Effect ranging from moderately less to small effect more. |
|  |  | ⊕⊕⊖⊖ |  |  |  |  |  |
|  |  |  |  |  |  |  |  |
|  |  |  |  |  |  |  |  |
|  |  |  |  |  |  |  |  |
| Risankizumab | 0.93 (0.64 to 1.35) | Very low | 681 per 1,000 | 628 per,1000 (439 to 891) | 5% less (24.6% less to 23.6% more) | NA | The data is very uncertain |
|  |  | ⊕⊖⊖⊖ |  |  |  |  |  |
|  |  |  |  |  |  |  |  |
| **GRADE Working Group grades of evidence**    **High certainty**: we are very confident that the true effect lies close to that of the estimate of the effect.    **Moderate certainty**: we are moderately confident in the effect estimate; the true effect is likely to be close to the estimate of the effect, but there is a possibility that it is substantially different.    **Low certainty**: our confidence in the effect estimate is limited; the true effect may be substantially different from the estimate of the effect.    **Very low certainty**: we have very little confidence in the effect estimate; the true effect is likely to be substantially different from the estimate of effect.    CI: confidence interval; RR: risk ratio    ^a^ The risk with placebo has been calculated based on the cumulative placebo rates of all studies with a placebo arm.  ^b^The risk with treatment has been calculated by multiplying the risk with control with the RR(95% CI). If the calculation results in more than 1000 per 1000 people the number has been capped to 1000. Numbers have been rounded up to the closest whole number.  ^c^The % risk difference has been calculated by subtracting the risk with control from the risk with treatment(95% CI) and dividing by 10. If the calculation results in more than 100% the number has been capped to 100%. Numbers have been rounded up to the closest whole number.  *red colouring indicates the treatment crosses the line of no effect | | | | | | | |
|  |  |  |  |  |  |  |  |
|  |  |  |  |  |  |  |  |
|  |  |  |  |  |  |  |  |
|  |  |  |  |  |  |  |  |
|  |  |  |  |  |  |  |  |
|  |  |  |  |  |  |  |  |
|  |  |  |  |  |  |  |  |
|  |  |  |  |  |  |  |  |
|  |  |  |  |  |  |  |  |

| **Sucra** | **Intervention (n=19)** | **network estimate RR** | **lower 95%CI** | **higher 95% CI** | **Number of direct studies** | **Direct GRADE** | **Reasons for direct downgrade** | **Indirect GRADE** | **Reasons for indirect downgrade** | **Network GRADE** | **Reasons for network downgrade** |
| --- | --- | --- | --- | --- | --- | --- | --- | --- | --- | --- | --- |
| **1** | Inflximab with purine analogues | 0.31 | 0.17 | 0.56 | 0 | x | x | very low | twice due to inconsistency, once due to rob | very low | none |
| **2** | CT-P13 (ifx biosimilar) | 0.54 | 0.4 | 0.73 | 1 | moderate | once rob | moderate | once due to rob | low | once incoherence |
| **3** | Infliximab | 0.62 | 0.5 | 0.76 | 3 | moderate | once due to rob | moderate | once rob | low | once incoherence |
| **4** | Methotrexate | 0.57 | 0.32 | 1.03 | 1 | high | none | x | x | low | twice imprecision |
| **5** | Methotrexate with infliximab | 0.64 | 0.37 | 1.12 | 0 | x | x | moderate | once rob | very low | twice imprecision |
| **6** | Vedolizumab with purine analogues | 0.65 | 0.42 | 1.03 | 0 | x | x | low | twice due to rob | very low | twice imprecision |
| **7** | Adalimumab | 0.68 | 0.55 | 0.84 | 4 | moderate | one due to rob | x | x | moderate | none |
| **8** | Upadacitinib | 0.72 | 0.52 | 1.02 | 1 | high | none | x | x | low | twice imprecision |
| **9** | Certolizumab | 0.73 | 0.51 | 1.04 | 1 | moderate | once due to rob | x | x | very low | twice imprecision |
| **10** | Vedolizumab | 0.77 | 0.56 | 1.06 | 2 | high | none | x | x | low | twice imprecision |
| **11** | Ustekinumab | 0.83 | 0.64 | 1.08 | 2 | high | none | x | x | low | twice imprecision |
| **12** | Azathioprine | 0.82 | 0.62 | 1.1 | 0 | moderate | once rob | low | twice due to rob | very low | twice imprecision |
| **13** | Natalizumab | 0.82 | 0.55 | 1.23 | 1 | moderate | once due to rob | x | x | very low | twice imprecision |
| **14** | Etrolizumab | 0.89 | 0.63 | 1.26 | 1 | high | none | x | x | low | twice imprecision |
| **15** | Filgotinib | 0.9 | 0.62 | 1.3 | 1 | moderate | once due to rob | x | x | Very low | Twice imprecision |
| **16** | Tofacitinib | 0.9 | 0.63 | 1.3 | 1 | high | none | x | x | low | twice imprecision |
| **17** | Risankizumab | 0.93 | 0.65 | 1.32 | 1 | moderate | once due to rob | x | x | very low | twice imprecision |
| **18** | 5-ASA | n/a | n/a | n/a | n/a | n/a | n/a | n/a | n/a | n/a | n/a |
| **19** | Placebo | 1 | n/a | n/a | n/a | n/a | n/a | n/a | n/a | n/a | n/a |
| **20** | Corticosteroids (budesonide) | n/a | n/a | n/a | n/a | n/a | n/a | n/a | n/a | n/a | n/a |

**Summary of Findings Tables and GRADE decisions (Clinical loss of response)**

| **Clinical loss of response** | | | | | | | |
| --- | --- | --- | --- | --- | --- | --- | --- |
| Patient or population: people with Crohn's disease in remission or response to treatment | | | | | | | |
| Settings: hospital setting | | | | | | | |
| Intervention: advanced therapies/purine analogues/methotrexate | | | | | | | |
| Comparison: placebo | | | | | | | |
| **Treatment** | **Network evidence** | | **Anticipated absolute effects for network estimate** | | | **NNT (95% CI)** | **Notes** |
|  | **RR** | **Certainty** | **Risk with Placebo^a^** | **Risk with Agent^b^ (95% CI)** | **% Risk Difference with Agent^c^  (95% CI)** |  |  |
|  | **(95% CI)** |  |  |  |  |  |  |
| CTP13 | 0.43 (0.35 to 0.53) | Moderate | 690 per 1,000 | 317 per 1,000 (262 to 393) | 39.1% less (44.6% less to 32.3% less) | 1 (1 to 2) | Probably large effect better than placebo (moderate to large) |
|  |  |  |  |  |  |  |  |
|  |  | ⊕⊕⊕⊖ |  |  |  |  |  |
| Natalizumab | 0.54 (0.43 to 0.68) | Moderate | 690 per 1,000 | 373 per 1,000 (297 to 469) | 31.7% less (39.4% less to 21.9% less) | 2 (1 to 2) | Probably moderate effect better than placebo (small to large) |
|  |  |  |  |  |  |  |  |
|  |  | ⊕⊕⊕⊖ |  |  |  |  |  |
| Certolizumab | 0.57 (0.47 to 0.7) | Moderate | 690 per 1,000 | 393 per 1,000 (324 to 483) | 29.3% (36.4% less to 20.7% less) | 2 (2 to 2) | Probably moderate effect better than placebo (small to large) |
|  |  |  |  |  |  |  |  |
|  |  | ⊕⊕⊕⊖ |  |  |  |  |  |
| Upadacitinib | 0.64 (0.57 to 0.72) | high | 690 per 1,000 | 442 per 1,000 (393 to 497) | 25.1% less (30% less to 19.6% less) | 2 (2 to 2) | Moderate effect better than placebo (small to moderate) |
|  |  | ⊕⊕⊕⊕ |  |  |  |  |  |
|  |  |  |  |  |  |  |  |
| Adalimumab | 0.68 (0.61 to 0.75) | Moderate | 690 per 1,000 | 469 per 1,000 (421 to 518) | 22.3% less (27.1% less to 16.9% less) | 2 (2 to 2) | Probably small effect better than placebo (small to moderate) |
|  |  |  |  |  |  |  |  |
|  |  | ⊕⊕⊕⊖ |  |  |  |  |  |
| Infliximab | 0.7 (0.59 to 0.82) | Low | 690 per 1,000 | 490 per 1,000 (407 to 580) | 20.9% less (28.1% less to 12.3% less) | NA | Maybe small effect better than placebo (trivial to moderate) |
|  |  |  |  |  |  |  |  |
|  |  | ⊕⊕⊖⊖ |  |  |  |  |  |
| Ustekinumab | 0.73 (0.61 to 0.86) | Moderate | 690 per 1,000 | 504 per 1,000 (421 to 593) | 18.8% less (26.6% less to 9.5% less) | 2 (2 to 3) | Probably small effect better than placebo (trivial to moderate) |
|  |  |  |  |  |  |  |  |
|  |  | ⊕⊕⊕⊖ |  |  |  |  |  |
| Vedolizumab | 0.82 (0.68 to 0.99) | Low | 690 per 1,000 | 566 per 1,000 (469 to 683) | 12.1% less (21.9% less to 0.4% less) | NA | Maybe trivial effect better than placebo (trivial to small) |
|  |  |  |  |  |  |  |  |
|  |  | ⊕⊕⊖⊖ |  |  |  |  |  |
| Filgotinib | 0.90 (0.76 to 1.05) | Very low | 690 per 1,000 | 621 per 1,000 | 7.2% less  (16.3% less to 3.4% more) | NA | The data is very uncertain |
|  |  | ⊕⊖⊖⊖ |  |  |  |  |  |
| Tofacitinib | 0.9 (0.74 to 1.09) | Low | 688 per 1,000 | 621 per 1,000 (511 to 752) | 6.9% less (17.9% less to 6.2% more) | NA | Maybe the same as placebo. Effect ranging from small effect less than placebo to trivially more than placebo. |
|  |  | ⊕⊕⊖⊖ |  |  |  |  |  |
|  |  |  |  |  |  |  |  |
|  |  |  |  |  |  |  |  |
|  |  |  |  |  |  |  |  |
| **GRADE Working Group grades of evidence**    **High certainty**: we are very confident that the true effect lies close to that of the estimate of the effect.    **Moderate certainty**: we are moderately confident in the effect estimate; the true effect is likely to be close to the estimate of the effect, but there is a possibility that it is substantially different.  **Low certainty**: our confidence in the effect estimate is limited; the true effect may be substantially different from the estimate of the effect.    **Very low certainty**: we have very little confidence in the effect estimate; the true effect is likely to be substantially different from the estimate of effect.        CI: confidence interval; RR: risk ratio    ^a^ The risk with placebo has been calculated based on the cumulative placebo rates of all studies with a placebo arm.  ^b^The risk with treatment has been calculated by multiplying the risk with control with the RR(95% CI). If the calculation results in more than 1000 per 1000 people the number has been capped to 1000. Numbers have been rounded up to the closest whole number.  ^c^The % risk difference has been calculated by subtracting the risk with control from the risk with treatment(95% CI) and dividing by 10. If the calculation results in more than 100% the number has been capped to 100%. Numbers have been rounded up to the closest whole number.  *red colouring indicates the treatment crosses the line of no effect | | | | | | | |
|  |  |  |  |  |  |  |  |
|  |  |  |  |  |  |  |  |
|  |  |  |  |  |  |  |  |
|  |  |  |  |  |  |  |  |
|  |  |  |  |  |  |  |  |
|  |  |  |  |  |  |  |  |
|  |  |  |  |  |  |  |  |
|  |  |  |  |  |  |  |  |

| **Sucra** | **Intervention (n=14)** | **network estimate RR** | **lower 95%CI** | **higher 95% CI** | **Number of direct studies** | **Direct GRADE** | **Reasons for direct downgrade** | **Indirect GRADE** | **Reasons for indirect downgrade** | **Network GRADE** | **Reasons for network downgrade** |
| --- | --- | --- | --- | --- | --- | --- | --- | --- | --- | --- | --- |
| 1 | ctp13 | 0.46 | 0.38 | 0.57 | 1 | moderate | once rob | moderate | once due to rob | moderate | none |
| 2 | natalizumab | 0.54 | 0.43 | 0.68 | 1 | moderate | once due to rob | x | x | moderate | none |
| 3 | certolizumab | 0.57 | 0.47 | 0.7 | 1 | moderate | once due to rob | x | x | moderate | none |
| 4 | upadacitinib | 0.64 | 0.57 | 0.72 | 1 | high | none | x | x | high | none |
| 5 | adalimumab | 0.68 | 0.61 | 0.75 | 3 | moderate | once due to rob | x | x | moderate | none |
| 6 | Infliximab | 0.71 | 0.59 | 0.84 | 2 | moderate | once due to rob | low | twice due to rob | low | once imprecision |
| 7 | ustekinumab | 0.73 | 0.61 | 0.86 | 2 | high | None | x | x | moderate | once imprecision |
| 8 | vedolizumab | 0.82 | 0.68 | 0.99 | 2 | moderate | once inconsistency | x | x | low | once imprecsion |
| 9 | filgotinib | 0.9 | 0.76 | 1.05 | 1 | moderate | Once due to rob | x | x | Very low | Twice imprecision |
| 10 | tofacitinib | 0.9 | 0.74 | 1.09 | 1 | high | none | x | x | low | Twice imprecision |
| 11 | placebo | n/a | n/a | n/a | n/a | n/a | n/a | n/a | n/a | n/a | n/a |

**Summary of Findings Tables and GRADE decisions (Withdrawals due to adverse events)**

| **Withdrawals due to adverse events** | | | | | | | |
| --- | --- | --- | --- | --- | --- | --- | --- |
| Patient or population: people with Crohn's disease in remission or response to treatment prior to randomisation, not biologically naïve | | | | | | | |
| Settings: hospital setting | | | | | | | |
| Intervention: advanced treatments/purine analogues/methotrexate | | | | | | | |
| Comparison: placebo | | | | | | | |
| **Treatment** | **Network evidence** | | **Anticipated absolute effects for network estimate** | | | **NNT (95% CI)** | **Notes** |
|  | **RR** | **Certainty** | **Risk with Placebo^a^** | **Risk with Agent^b^ (95% CI)** | **% Risk Difference with Agent^c^  (95% CI)** |  |  |
|  | **(95% CI)** |  |  |  |  |  |  |
| Natalizumab | 0.48 (0.22 to 1.05) | Very low | 189 per 1,000 | 94 per 1,000 (43 to 206) | 11.1% less (16.6% less to 1% more) | NA | The evidence is very uncertain |
|  |  | ⊕⊖⊖⊖ |  |  |  |  |  |
|  |  |  |  |  |  |  |  |
| Certolizumab | 0.58 (0.28 to 1.2) | Very low | 189 per 1,000 | 114 per 1,000 (55 to 235) | 8.8% less (15.2% less to 4.2% more) | NA | The evidence is very uncertain |
|  |  | ⊕⊖⊖⊖ |  |  |  |  |  |
|  |  |  |  |  |  |  |  |
| Ustekinumab | 0.72 (0.34 to 1.53) | Low | 189 per 1,000 | 141 per 1,000 (67 to 300) | 5.9% less (13.9% less to 11.2% more) | NA | Maybe the same as placebo. Effect ranging from small effect less to small more |
|  |  |  |  |  |  |  |  |
|  |  | ⊕⊕⊖⊖ |  |  |  |  |  |
| Infliximab | 0.78 (0.41 to 1.51) | Very low | 189 per 1,000 | 153 per 1,000 (80 to 296) | 4.6% less (12.5% less to 10.8% more) | NA | The evidence is very uncertain |
|  |  | ⊕⊖⊖⊖ |  |  |  |  |  |
|  |  |  |  |  |  |  |  |
| Tofacitinib | 0.78 (0.36 to 1.69) | Low | 189 per 1,000 | 153 per 1,000 (71 to 331) | 4.6% less (13.5% less to 14.6% more) | NA | Maybe the same as placebo. Effect ranging from small less to small more |
|  |  |  |  |  |  |  |  |
|  |  | ⊕⊕⊖⊖ |  |  |  |  |  |
| Vedolizumab | 0.82 (0.52 to 1.29) | Moderate | 189 per 1,000 | 161 per 1,000 (102 to 253) | 3.9% less (10.2% less to 6% more) | NA | Maybe the same as placebo. Effect ranging from small less to trivial more |
|  |  |  |  |  |  |  |  |
|  |  | ⊕⊕⊕⊖ |  |  |  |  |  |
| Risankizumab | 0.87 (0.32 to 2.42) | Very low | 189 per 1,000 | 165 per 1,000 (59 to 455) | 2.7% less (14.5% less to 30.2% more) | NA | The evidence is very uncertain |
|  |  | ⊕⊖⊖⊖ |  |  |  |  |  |
|  |  |  |  |  |  |  |  |
| Adalimumab | 1.01 (0.56 to 1.84) | Very low | 189 per 1,000 | 198 per 1,000 (110 to 361) | 0.2% more (9.4% less to 17.8% more) | NA | The evidence is very uncertain |
|  |  | ⊕⊖⊖⊖ |  |  |  |  |  |
|  |  |  |  |  |  |  |  |
| CT-P13 | 1.02 (0.27 to 3.86) | Very low | 189 per 1,000 | 200 per 1,000 (53 to 757) | 0.4% more (15.5% less to 60.6% more) | NA | The evidence is very uncertain |
|  |  | ⊕⊖⊖⊖ |  |  |  |  |  |
|  |  |  |  |  |  |  |  |
| Updacitinib | 1.51 (0.6 to 3.8) | Low | 189 per 1,000 | 296 per 1,000 (118 to 745) | 10.8% more (8.5% less to 59.3% more) | NA | Maybe the same as placebo. Effect ranging from small less to large more |
|  |  |  |  |  |  |  |  |
|  |  | ⊕⊕⊖⊖ |  |  |  |  |  |
| Etrolizumab | 1.42 (0.59 to 3.42) | Low | 189 per 1,000 | 278 per 1,000 (116 to 670) | 8.9% more (8.7% less to 51.3% more) | NA | Maybe the same as placebo. Effect ranging from small less to large more |
|  |  |  |  |  |  |  |  |
|  |  | ⊕⊕⊖⊖ |  |  |  |  |  |
| Purine analogues | 1.82 (0.84 to 3.94) | Very low | 189 per 1,000 | 357 per 1,000 (165 to 772) | 17.4% more (3.3% less to 62.4% more) | NA | The evidence is very uncertain |
|  |  | ⊕⊖⊖⊖ |  |  |  |  |  |
|  |  |  |  |  |  |  |  |
| Filgotinib | 2.51 (0.89 to 7.06) | Very low | 189 per 1,000 | 474 per 1,000 (168 to 1000) | 32% more  (2.3% less to 100% more) | NA | The evidence is very uncertain |
|  |  | ⊕⊖⊖⊖ |  |  |  |  |  |
| Infliximab with purine analogues | 2.31 (0.79 to 6.78) | Very low | 189 per 1,000 | 453 per 1,000 (155 to 1000) | 27.8% less (4.5% less to 100% more) | NA | The evidence is very uncertain |
|  |  | ⊕⊖⊖⊖ |  |  |  |  |  |
|  |  |  |  |  |  |  |  |
| **GRADE Working Group grades of evidence**    **High certainty**: we are very confident that the true effect lies close to that of the estimate of the effect.  **Moderate certainty**: we are moderately confident in the effect estimate; the true effect is likely to be close to the estimate of the effect, but there is a possibility that it is substantially different.  **Low certainty**: our confidence in the effect estimate is limited; the true effect may be substantially different from the estimate of the effect.  **Very low certainty**: we have very little confidence in the effect estimate; the true effect is likely to be substantially different from the estimate of effect.    CI: confidence interval; RR: risk ratio    ^a^ The risk with placebo has been calculated based on the cumulative placebo rates of all studies with a placebo arm.  ^b^The risk with treatment has been calculated by multiplying the risk with control with the RR(95% CI). If the calculation results in more than 1000 per 1000 people the number has been capped to 1000. Numbers have been rounded up to the closest whole number.  ^c^The % risk difference has been calculated by subtracting the risk with control from the risk with treatment(95% CI) and dividing by 10. If the calculation results in more than 100% the number has been capped to 100%. Numbers have been rounded up to the closest whole number.  *red colouring indicates the treatment crosses the line of no effect | | | | | | | |

| **Sucra** | **Intervention (n=15)** | **network estimate RR** | **lower 95%CI** | **higher 95% CI** | **Number of direct studies** | **Direct GRADE** | **Reasons for direct downgrade** | **Indirect GRADE** | **Reasons for indirect downgrade** | **Network GRADE** | **Reasons for network downgrade** |
| --- | --- | --- | --- | --- | --- | --- | --- | --- | --- | --- | --- |
| 1 | Natalizumab | 0.48 | 0.22 | 1.05 | 1 | moderate | once rob | x | x | **very low** | twice imprecision |
| 2 | Certolizumab | 0.58 | 0.28 | 1.2 | 1 | moderate | once rob | x | x | **very low** | twice imprecision |
| 3 | Corticosteroids | n/a | n/a | n/a | n/a | n/a | n/a | n/a | n/a | n/a | n/a |
| 4 | Ustekinumab | 0.72 | 0.34 | 1.53 | 2 | high | none | x | x | **low** | twice imprecision |
| 5 | Infliximab | 0.78 | 0.41 | 1.51 | 2 | moderate | once rob | moderate | once rob | **very low** | twice imprecision, once incoherence |
| 6 | Tofacitinib | 0.78 | 0.36 | 1.69 | 1 | high | none | x | x | **low** | twice imprecision |
| 7 | Vedolizumab | 0.82 | 0.52 | 1.29 | 3 | high | none | x | x | **moderate** | once imprecision |
| 8 | Risankizumab | 0.84 | 0.3 | 2.32 | 1 | moderate | once rob | x | x | **very low** | twice imprecision |
| 9 | Adalimumab | 1.01 | 0.56 | 1.84 | 3 | moderate | once rob | moderate | once rob | **very low** | twice imprecision, once incoherence |
| 10 | Placebo | 1 | n/a | n/a | n/a | n/a | n/a | n/a | n/a | n/a | n/a |
| 11 | CT-P13 | 1.02 | 0.27 | 3.86 | 1 | moderate | once rob | moderate | once rob | **very low** | twice imprecision |
| 12 | 5-ASA | n/a | n/a | n/a | n/a | n/a | n/a | n/a | n/a | n/a | n/a |
| 13 | Updacitinib | 1.51 | 0.6 | 3.8 | 1 | high | none | high | none | **low** | twice imprecision |
| 14 | Etrolizumab | 1.42 | 0.59 | 3.42 | 1 | high | none | x | x | **low** | twice imprecision |
| 15 | Purine analogues | 1.82 | 0.84 | 3.94 | 5 | moderate | once rob | low | twice rob | **very low** | twice imprecision, twice incoherence |
| 16 | Filgotinib | 2.51 | 0.89 | 7.06 | 1 | moderate | Once rob | x | x | **Very low** | Twice imprecision |
| 17 | Infliximab with purine analogues | 2.31 | 0.79 | 6.78 | 0 | x | x | moderate | once rob | **very low** | twice imprecision |

**Summary of Findings Tables and GRADE decisions (Serious adverse events)**

| **Serious adverse events** | | | | | | | |
| --- | --- | --- | --- | --- | --- | --- | --- |
| Patient or population: people with Crohn's disease in remission or response to treatment prior to randomisation, not biologically naïve | | | | | | | |
| Settings: hospital setting | | | | | | | |
| Intervention: advanced treatments/purine analogues/methotrexate | | | | | | | |
| Comparison: placebo | | | | | | | |
| **Treatment** | **Network evidence** | | **Anticipated absolute effects for network estimate** | | | **NNT (95% CI)** | **Notes** |
|  | **RR** | **Certainty** | **Risk with Placebo^a^** | **Risk with Agent^b^ (95% CI)** | **% Risk Difference with Agent^c^  (95% CI)** |  |  |
|  | **(95% CI)** |  |  |  |  |  |  |
| Adalimumab | 0.53 (0.2 to 1.43) | Low | 114 per 1,000 | 61 per 1,000 (22 to 166) | 5.4% less (9.2% less to 4.9% more) | NA | Maybe the same as placebo. Effect ranging from small effect less to trivial more |
|  |  | ⊕⊖⊖⊖ |  |  |  |  |  |
|  |  |  |  |  |  |  |  |
| Vedolizumab | 0.7 ( 0.25 to 1.92) | Low | 114 per 1,000 | 81 per 1,000 (29 to 223) | 3.5% less (8.5% less to 10.5% more) | NA | Maybe the same as placebo. Effect ranging from small less to moderate more. |
|  |  |  |  |  |  |  |  |
|  |  | ⊕⊕⊖⊖ |  |  |  |  |  |
| Ustekinumab | 0.73 (0.23 to 2.37) | Low | 114 per 1,000 | 85 per 1,000 (27 to 275) | 3.1% less (8.8% less to 15.6% more) | NA | Maybe the same as placebo. Effect ranging from small less to moderate more. |
|  |  |  |  |  |  |  |  |
|  |  | ⊕⊕⊖⊖ |  |  |  |  |  |
| Natalizumab | 0.86 (0.26 to 2.87) | Very low | 114 per 1,000 | 100 per 1,000 (30 to 333) | 1.6% less (8.5% less to 21.3% more) | NA | The evidence is very uncertain |
|  |  | ⊕⊖⊖⊖ |  |  |  |  |  |
|  |  |  |  |  |  |  |  |
| Updacitinib | 0.8 (0.26 to 2.47) | Low | 114 per 1,000 | 93 per 1,000 (30 to 287) | 2.3% less (8.5% less to 16.8% more) | NA | Maybe the same as placebo. Effect ranging from small less to moderate more. |
|  |  |  |  |  |  |  |  |
|  |  | ⊕⊕⊖⊖ |  |  |  |  |  |
| Certolizumab | 0.84 (0.23 to 3.05) | Very low | 114 per 1,000 | 97 per 1,000 (27 to 354) | 1.8% less (8.8% less to 23.4% more) | NA | The evidence is very uncertain |
|  |  | ⊕⊖⊖⊖ |  |  |  |  |  |
|  |  |  |  |  |  |  |  |
| Etrolizumab | 1 (0.25 to 3.99) | Low | 114 per 1,000 | 116 per 1,000 (29 to 463) | 0% (8.5% less to 34.1% more) | NA | Maybe the same as placebo. Effect ranging from small less to large more |
|  |  | ⊕⊕⊖⊖ |  |  |  |  |  |
|  |  |  |  |  |  |  |  |
| Tofacitinib | 0.94 (0.28 to 3.13) | Low | 114 per 1,000 | 109 per 1,000 (32 to 363) | 0.7% less (8.2% less to 24.3% more) | NA | Maybe the same as placebo. Effect ranging from small less to large more. |
|  |  |  |  |  |  |  |  |
|  |  | ⊕⊕⊖⊖ |  |  |  |  |  |
| Risankizumab | 1.03 (0.33 to 3.24) | Very low | 114 per 1,000 | 119 per 1,000 (38 to 376) | 0.3% less (7.7% less to 25.5% more) | NA | The evidence is very uncertain |
|  |  | ⊕⊖⊖⊖ |  |  |  |  |  |
|  |  |  |  |  |  |  |  |
| CT-P13 | 1.38 (0.46 to 4.14) | Very low | 114 per 1,000 | 160 per 1,000 (53 to 480) | 4.3% more (6.2% less to 35.8% more) | NA | The evidence is very uncertain |
|  |  | ⊕⊖⊖⊖ |  |  |  |  |  |
|  |  |  |  |  |  |  |  |
| Filgotinib | 1.70 (0.47 to 6.17) | Very low | 114 per 1,000 | 194 per 1,000 (54 to 860) | 7.9% more  (6.1% less to 59% more) | NA | The evidence is very uncertain |
|  |  | ⊕⊖⊖⊖ |  |  |  |  |  |
| Infliximab with purine analogues | 1.88 (0.47 to 7.54) | Very low | 114 per 1,000 | 218 per 1,000 (55 to 875) | 10% more (6.1% less to 74.6% more) | NA | The evidence is very uncertain |
|  |  | ⊕⊖⊖⊖ |  |  |  |  |  |
|  |  |  |  |  |  |  |  |
| Purine analogues | 1.74 (0.88 to 3.46) | Very low | 114 per 1,000 | 202 per 1,000 (102 to 401) | 8.4% more (1.4% less to 28% more) | NA | The evidence is very uncertain |
|  |  | ⊕⊖⊖⊖ |  |  |  |  |  |
|  |  |  |  |  |  |  |  |
| Vedolizumab with purine analogues | 1.99 (0.53 to 7.55) | Very low | 114 per 1,000 | 231 per 1,000 (61 to 876) | 11.3% more (5.4% less to 74.6% more) | NA | The evidence is very uncertain |
|  |  | ⊕⊖⊖⊖ |  |  |  |  |  |
|  |  |  |  |  |  |  |  |
| Infliximab | 2.23 (0.73 to 6.81) | Very low | 114 per 1,000 | 259 per 1,000 (85 to 790) | 14% more (3.1% less to 66.2% more) | NA | The evidence is very uncertain |
|  |  | ⊕⊖⊖⊖ |  |  |  |  |  |
|  |  |  |  |  |  |  |  |
| **GRADE Working Group grades of evidence**    **High certainty**: we are very confident that the true effect lies close to that of the estimate of the effect.    **Moderate certainty**: we are moderately confident in the effect estimate; the true effect is likely to be close to the estimate of the effect, but there is a possibility that it is substantially different.  **Low certainty**: our confidence in the effect estimate is limited; the true effect may be substantially different from the estimate of the effect.    **Very low certainty**: we have very little confidence in the effect estimate; the true effect is likely to be substantially different from the estimate of effect.    CI: confidence interval; RR: risk ratio  ^a^ The risk with placebo has been calculated based on the cumulative placebo rates of all studies with a placebo arm.  ^b^The risk with treatment has been calculated by multiplying the risk with control with the RR(95% CI). If the calculation results in more than 1000 per 1000 people the number has been capped to 1000. Numbers have been rounded up to the closest whole number.  ^c^The % risk difference has been calculated by subtracting the risk with control from the risk with treatment(95% CI) and dividing by 10. If the calculation results in more than 100% the number has been capped to 100%. Numbers have been rounded up to the closest whole number.  *red colouring indicates the treatment crosses the line of no effect | | | | | | | |

| **Sucra** | **Intervention (n=16)** | **network estimate RR** | **lower 95%CI** | **higher 95% CI** | **Number of direct studies** | **Direct GRADE** | **Reasons for direct downgrade** | **Indirect GRADE** | **Reasons for indirect downgrade** | **Network GRADE** | **Reasons for network downgrade** |
| --- | --- | --- | --- | --- | --- | --- | --- | --- | --- | --- | --- |
| 1 | 5-ASA | n/a | n/a | n/a | n/a | n/a | n/a | n/a | n/a | n/a | n/a |
| 2 | Adalimumab | 0.53 | 0.2 | 1.43 | 3 | moderate | once rob | low | twice rob | **low** | once imprecision |
| 3 | Vedolizumab | 0.7 | 0.25 | 1.92 | 2 | high | none | x | x | **low** | twice imprecision |
| 4 | Corticosteroids | n/a | n/a | n/a | n/a | n/a | n/a | n/a | n/a | n/a | n/a |
| 5 | Ustekinumab | 0.73 | 0.23 | 2.37 | 1 | high | none | x | x | **low** | twice imprecision |
| 6 | Natalizumab | 0.86 | 0.26 | 2.87 | 1 | moderate | once rob | x | x | **very low** | twice imprecision |
| 7 | Updacitinib | 0.8 | 0.26 | 2.47 | 1 | high | none | x | x | **low** | twice imprecision |
| 8 | Certolizumab | 0.84 | 0.23 | 3.05 | 1 | moderate | once rob | x | x | **very low** | twice imprecision |
| 9 | Etrolizumab | 1 | 0.25 | 3.99 | 1 | high | none | x | x | **low** | twice imprecision |
| 10 | Tofacitinib | 0.94 | 0.28 | 3.13 | 1 | high | none | x | x | **low** | twice imprecision |
| 11 | Risankizumab | 1.03 | 0.33 | 3.24 | 1 | moderate | once rob | x | x | **very low** | twice imprecision |
| 12 | Placebo | 1 | n/a | n/a | n/a | n/a | n/a | n/a | n/a | n/a | n/a |
| 13 | CT-P13 | 1.38 | 0.46 | 4.14 | 1 | moderate | once rob | moderate | once rob | **very low** | twice imprecision |
| 14 | Filgotinib | 1.7 | 0.47 | 6.17 | 1 | Moderate | Once rob | x | x | **Very low** | Twice imprecision |
| 15 | Infliximab with purine analogues | 1.88 | 0.47 | 7.54 | 0 | x | x | moderate | once rob | **very low** | twice imprecision |
| 16 | Purine analogues | 1.74 | 0.88 | 3.46 | 4 | low | once inconsistency, once rob | |  | **very low** | twice imprecision |
| 17 | Vedolizumab with purine analogues | 1.99 | 0.53 | 7.55 | 0 | x | x | low | twice rob | **very low** | twice imprecision |
| 18 | Infliximab | 2.23 | 0.73 | 6.81 | 0 | x | x | moderate | once rob | **very low** | twice imprecision |

**Summary of Findings Tables and GRADE decisions (Total adverse events)**

| **Total adverse events** | | | | | | | |
| --- | --- | --- | --- | --- | --- | --- | --- |
| Patient or population: people with Crohn's disease in remission or response to treatment prior to randomisation, not biologically naïve | | | | | | | |
| Settings: hospital setting | | | | | | | |
| Intervention: advanced treatments/purine analogues/methotrexate | | | | | | | |
| Comparison: placebo | | | | | | | |
| **Treatment** | **Network evidence** | | **Anticipated absolute effects for network estimate** | | | **NNT (95% CI)** | **Notes** |
|  | **RR** | **Certainty** | **Risk with Placebo^a^** | **Risk with Agent^b^ (95% CI)** | **% Risk Difference with Agent^c^  (95% CI)** |  |  |
|  | **(95% CI)** |  |  |  |  |  |  |
| Natalizumab | 0.94 (0.69 to 1.28) | Very low | 745 per 1,000 | 707 per 1,000 (519 to 963) | 4.7% less (23.4% less to 20.9 % more) | NA | The evidence is very uncertain. |
|  |  | ⊕⊖⊖⊖ |  |  |  |  |  |
|  |  |  |  |  |  |  |  |
| Adalimumab | 0.94 (0.78 to 1.14) | Very low | 745 per 1,000 | 707 per 1,000 (587 to 857) | 4.2% less (16.2% less to 10.2% more) | NA | The evidence is very uncertain. |
|  |  | ⊕⊖⊖⊖ |  |  |  |  |  |
|  |  |  |  |  |  |  |  |
| CT-P13 | 0.97 (0.75 to 1.27) | Very low | 745 per 1,000 | 729 per 1,000 (564 to 955) | 1.9% less (18.9% less to 20.3% more) | NA | The evidence is very uncertain. |
|  |  | ⊕⊖⊖⊖ |  |  |  |  |  |
|  |  |  |  |  |  |  |  |
| Vedolizumab | 0.95 (0.72 to 1.26) | Low | 745 per 1,000 | 714 per 1,000 (541 to 948) | 3.7% less (20.9% less to 19% more) | NA | Maybe the same as placebo. Effect ranging from moderate less to moderate more. |
|  |  |  |  |  |  |  |  |
|  |  | ⊕⊕⊖⊖ |  |  |  |  |  |
| Etrolizumab | 0.99 (0.72 to 1.36) | Low | 745 per 1,000 | 744 per 1,000 (541 to 1000) | 0.4% less (20.5% less to 27.2% more) | NA | Maybe the same as placebo. Effect ranging from moderate less to large more. |
|  |  |  |  |  |  |  |  |
|  |  | ⊕⊕⊖⊖ |  |  |  |  |  |
| Certolizumab | 0.96 (0.69 to 1.35) | Very low | 745 per 1,000 | 722 per 1,000 (519 to 1000) | 2.9% less (23.4% less to 25.7% more) | NA | The evidence is very uncertain. |
|  |  | ⊕⊖⊖⊖ |  |  |  |  |  |
|  |  |  |  |  |  |  |  |
| Ustekinumab | 0.97 (0.7 to 1.33) | Low | 745 per 1,000 | 729 per 1,000 (526 to 1000) | 2.5% less (22.3% less to 25% more) | NA | Maybe the same as placebo. Effect ranging from moderate less to large more. |
|  |  |  |  |  |  |  |  |
|  |  | ⊕⊕⊖⊖ |  |  |  |  |  |
| Risankizumab | 0.98 (0.71 to 1.36) | Low | 745 per 1,000 | 737 per 1,000 (534 to 1000) | 1.6% less (21.9% less to 26.6% more) | NA | Maybe the same as placebo. Effect ranging from trivial less to moderate more. |
|  |  |  |  |  |  |  |  |
|  |  | ⊕⊕⊖⊖ |  |  |  |  |  |
| Updacitinib | 1 (0.73 to 1.38) | Low | 745 per 1,000 | 752 per 1,000 (549 to 1000) | 0% (20.5% less to 28.2% more) | NA | Maybe the same as placebo. Effect ranging from moderate less to large more. |
|  |  |  |  |  |  |  |  |
|  |  | ⊕⊕⊖⊖ |  |  |  |  |  |
| Filgotinib | 1.10 (0.77 to 1.56) | Very low | 745 per 1,000 | 820 per 1,000 (574 to 1000) | 7.2% more  (16.9% less to 41.4% more) | NA | The evidence is very uncertain |
|  |  | ⊕⊖⊖⊖ |  |  |  |  |  |
| Tofacitinib | 1.09 (0.76 to 1.55) | Low | 745 per 1,000 | 820 per 1,000 (572 to 1000) | 6.4% more (17.7% less to 40.7% more) | NA | Maybe the same as placebo. Effect ranging from moderate less to large more. |
|  |  |  |  |  |  |  |  |
|  |  | ⊕⊕⊖⊖ |  |  |  |  |  |
| Infliximab | 1.09 (0.86 to 1.36) | Very low | 745 per 1,000 | 820 per 1,000 (647 to 1000) | 6.4% less (10.1% less to 27.1% more) | NA | The evidence is very uncertain. |
|  |  | ⊕⊖⊖⊖ |  |  |  |  |  |
|  |  |  |  |  |  |  |  |
| Purine analogues | 1.34 (0.93 to 1.93) | Very low | 745 per 1,000 | 1000 per 1,000 (1000to 1000) | 25.1% more (5.5% less to 69.3% more) | NA | The evidence is very uncertain. |
|  |  | ⊕⊖⊖⊖ |  |  |  |  |  |
|  |  |  |  |  |  |  |  |
| **GRADE Working Group grades of evidence**    **High certainty**: we are very confident that the true effect lies close to that of the estimate of the effect.    **Moderate certainty**: we are moderately confident in the effect estimate; the true effect is likely to be close to the estimate of the effect, but there is a possibility that it is substantially different.  **Low certainty**: our confidence in the effect estimate is limited; the true effect may be substantially different from the estimate of the effect.    **Very low certainty**: we have very little confidence in the effect estimate; the true effect is likely to be substantially different from the estimate of effect.    CI: confidence interval; RR: risk ratio    ^a^ The risk with placebo has been calculated based on the cumulative placebo rates of all studies with a placebo arm.  ^b^The risk with treatment has been calculated by multiplying the risk with control with the RR(95% CI). If the calculation results in more than 1000 per 1000 people the number has been capped to 1000. Numbers have been rounded up to the closest whole number.  ^c^The % risk difference has been calculated by subtracting the risk with control from the risk with treatment(95% CI) and dividing by 10. If the calculation results in more than 100% the number has been capped to 100%. Numbers have been rounded up to the closest whole number.  *red colouring indicates the treatment crosses the line of no effect | | | | | | | |

| **Sucra** | **Intervention (n=12)** | **network estimate RR** | **lower 95%CI** | **higher 95% CI** | **Number of direct studies** | **Direct GRADE** | **Reasons for direct downgrade** | **Indirect GRADE** | **Reasons for indirect downgrade** | **Network GRADE** | **Reasons for network downgrade** |
| --- | --- | --- | --- | --- | --- | --- | --- | --- | --- | --- | --- |
| 1 | Natalizumab | 0.94 | 0.69 | 1.28 | 1 | moderate | once rob | x | x | **very low** | twice imprecision |
| 2 | Adalimumab | 0.94 | 0.78 | 1.14 | 3 | very low | twice inconsistency, once rob | moderate | once rob | **very low** | twice imprecision, once incoherence |
| 3 | CT-P13 | 0.97 | 0.75 | 1.27 | 1 | moderate | once rob | moderate | once rob | **very low** | twice imprecision, twice incoherence |
| 4 | Vedolizumab | 0.95 | 0.72 | 1.26 | 2 | high | none | x | x | **low** | twice imprecision |
| 5 | Etrolizumab | 0.99 | 0.72 | 1.36 | 1 | high | none | x | x | **low** | twice imprecision |
| 6 | Certolizumab | 0.96 | 0.69 | 1.35 | 1 | moderate | once rob | x | x | **very low** | twice imprecision |
| 7 | Ustekinumab | 0.97 | 0.7 | 1.33 | 1 | high | none | x | x | **low** | twice imprecision |
| 8 | Risankizumab | 0.98 | 0.71 | 1.36 | 1 | moderate | once rob | x | x | **low** | twice imprecision |
| 9 | Placebo | 1 | n/a | n/a | n/a | n/a | n/a | n/a | n/a | n/a | n/a |
| 10 | Updacitinib | 1 | 0.73 | 1.38 | 1 | high | none | x | x | **low** | twice imprecision |
| 11 | Filgotinib | 1.1 | 0.77 | 1.56 | 1 | moderate | Once rob | x | x | **Very low** | Twice imprecision |
| 12 | Tofacitinib | 1.09 | 0.76 | 1.55 | 1 | high | none | x | x | **low** | twice imprecision |
| 13 | Infliximab | 1.09 | 0.86 | 1.36 | 1 | moderate | once rob | moderate | once rob | **very low** | twice imprecision, once incoherence |
| 14 | Purine analogues | 1.34 | 0.93 | 1.93 | 5 | moderate | once rob | x | x | **very low** | twice imprecision |

**eFigures 1. GORDON PLOTS**

**
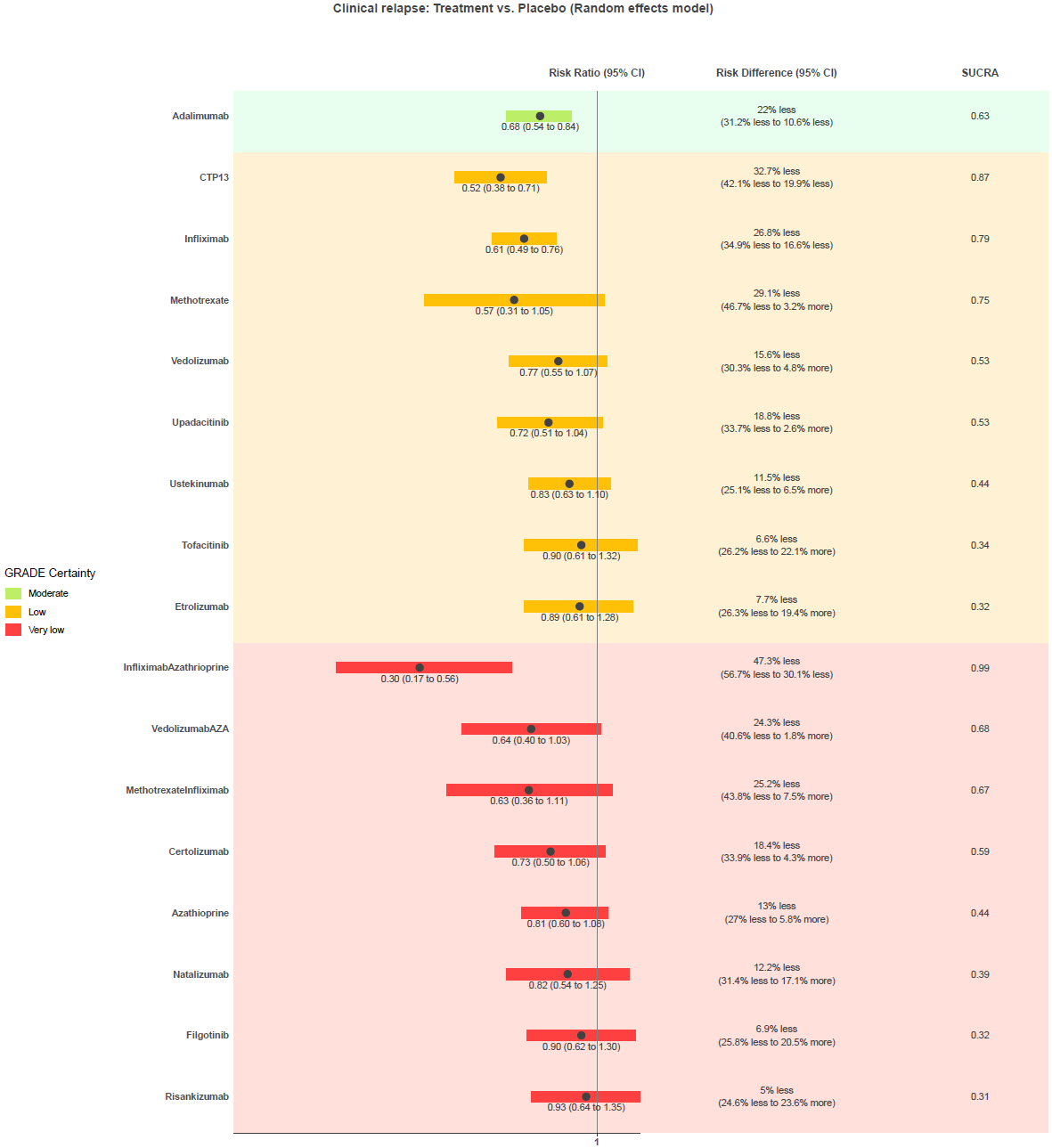
**

**
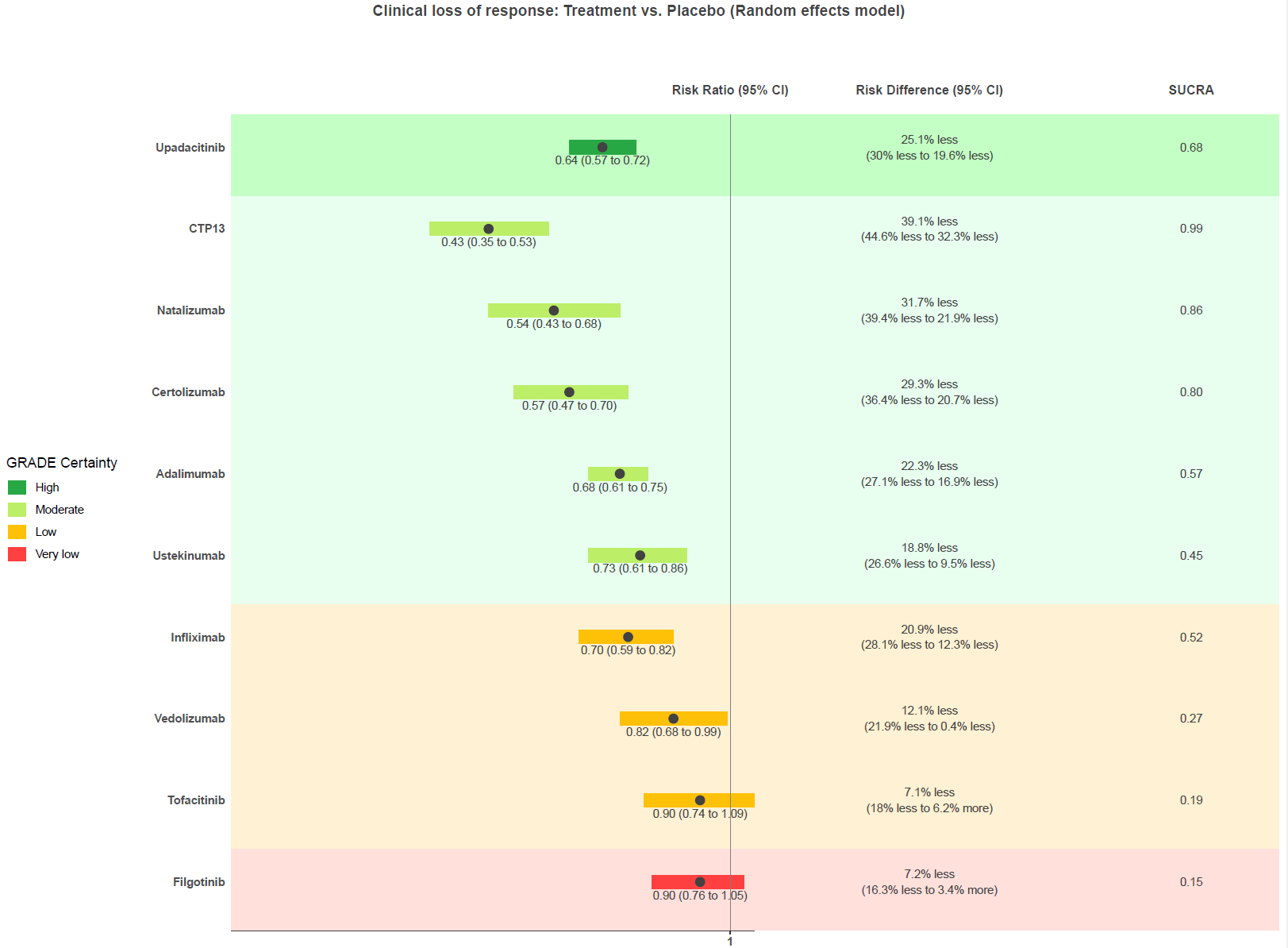
**

**
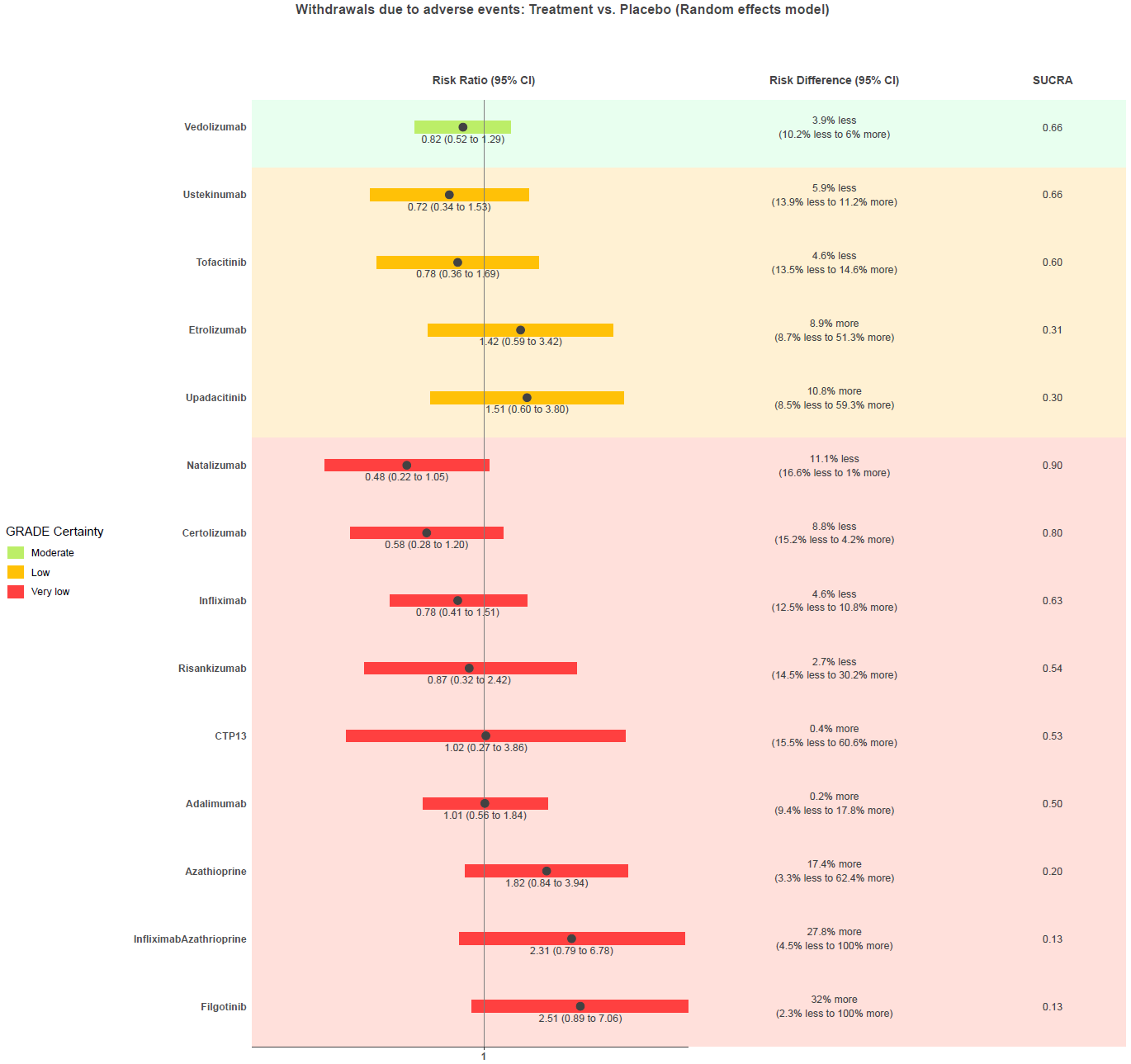
**

**
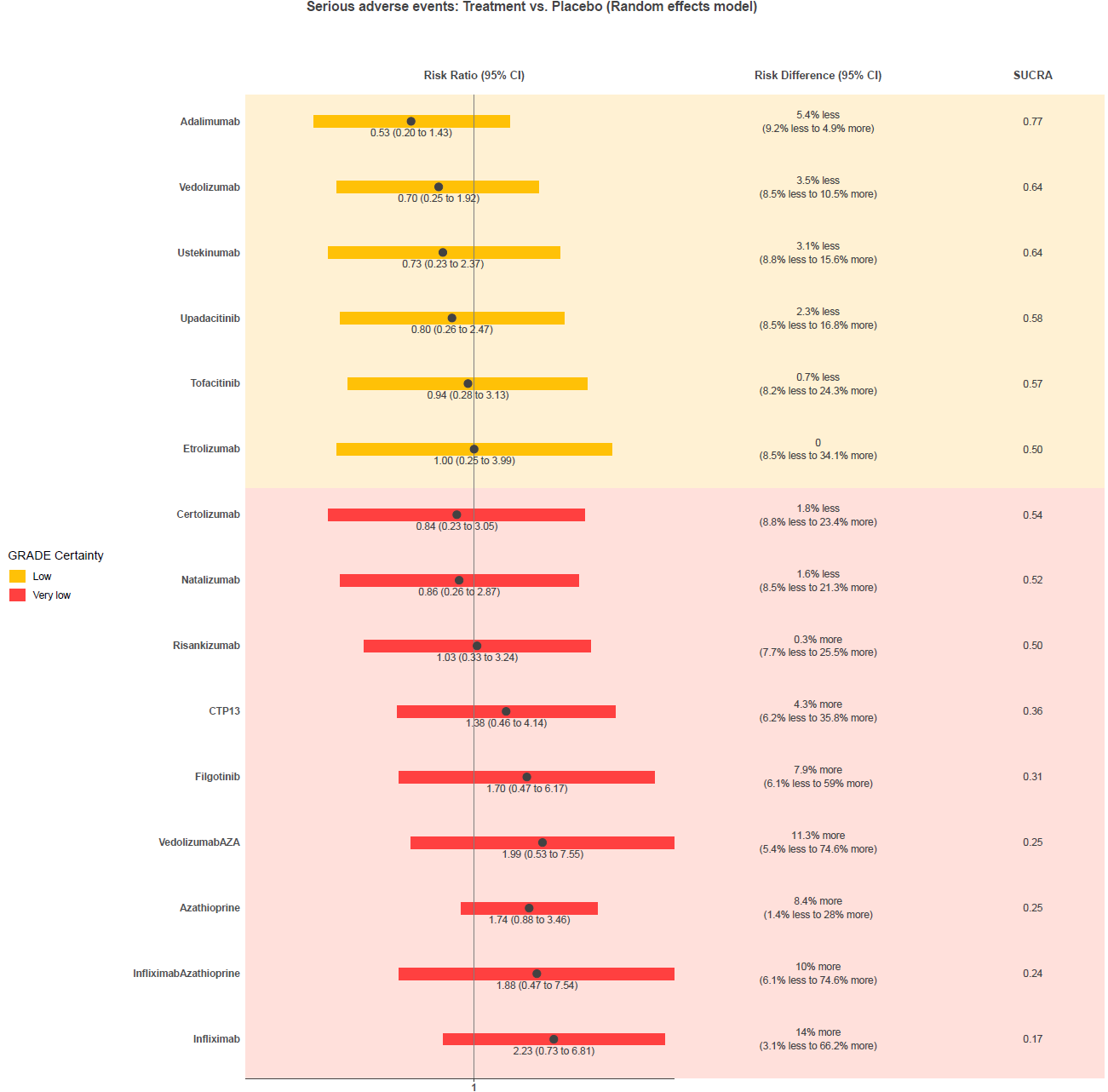
**

**
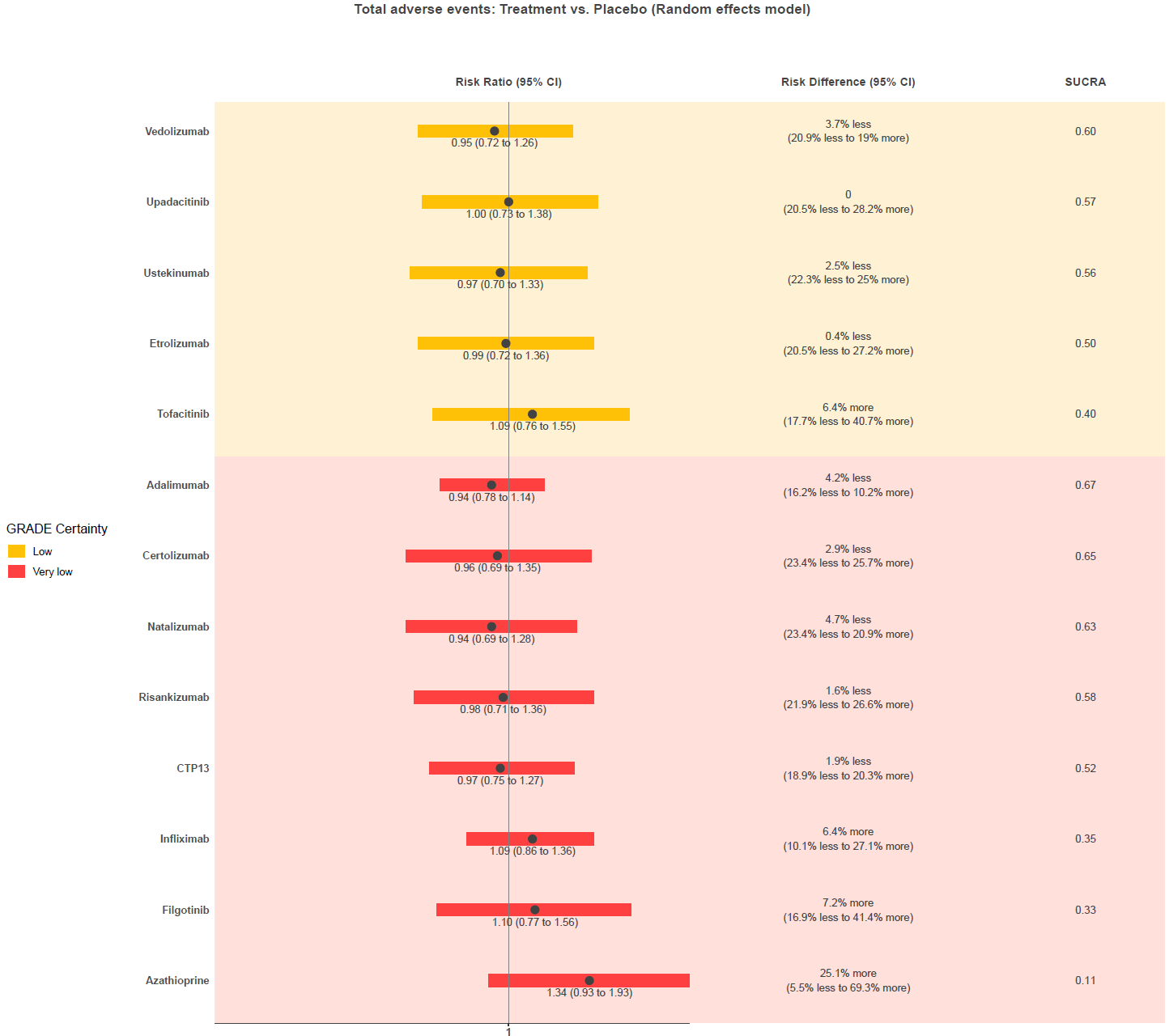
**

**eFigures 2**. **NETWORK PLOTS**

**Network plot for Clinical Relapse**

**
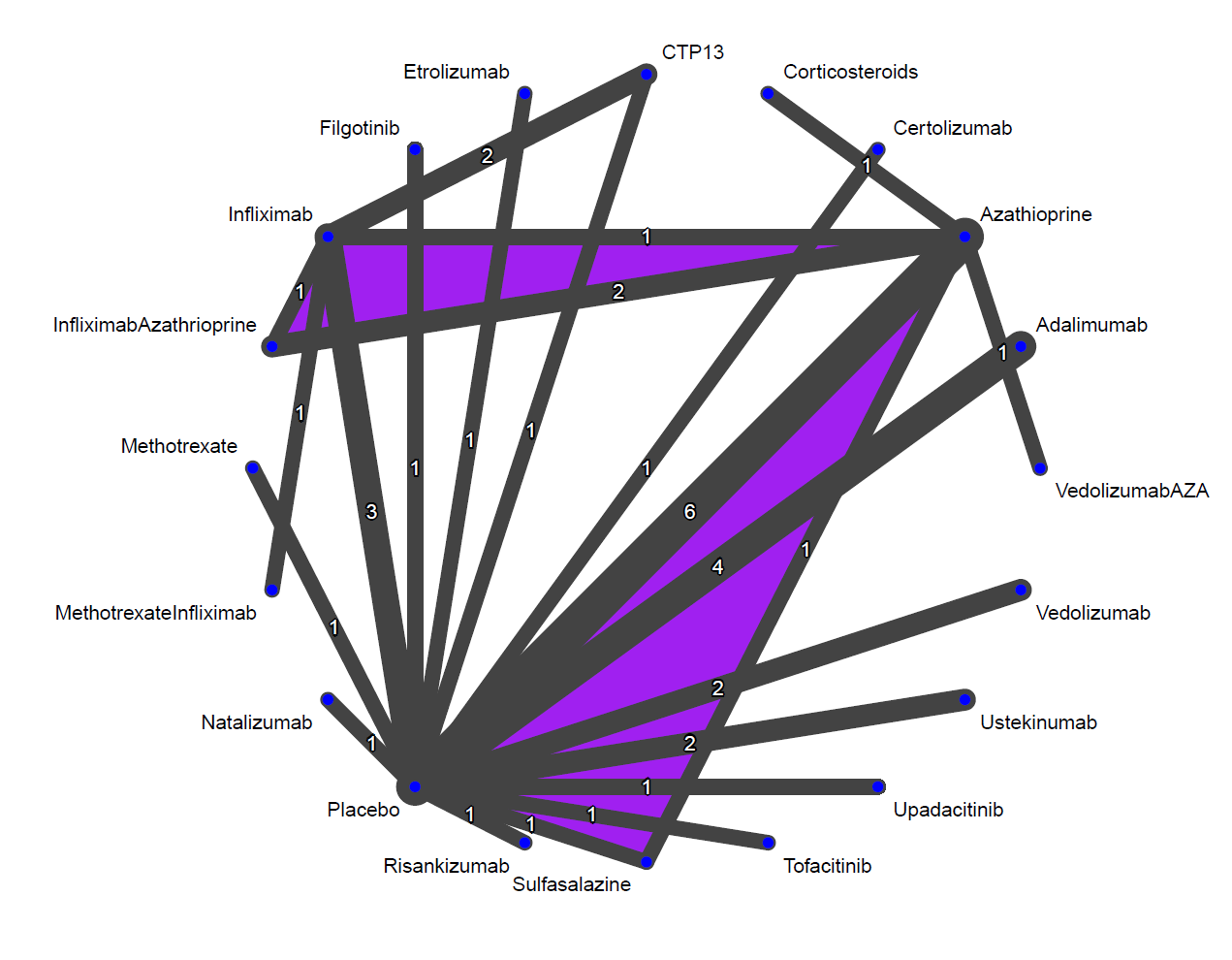
**

**Network plot for Clinical Loss of Response**


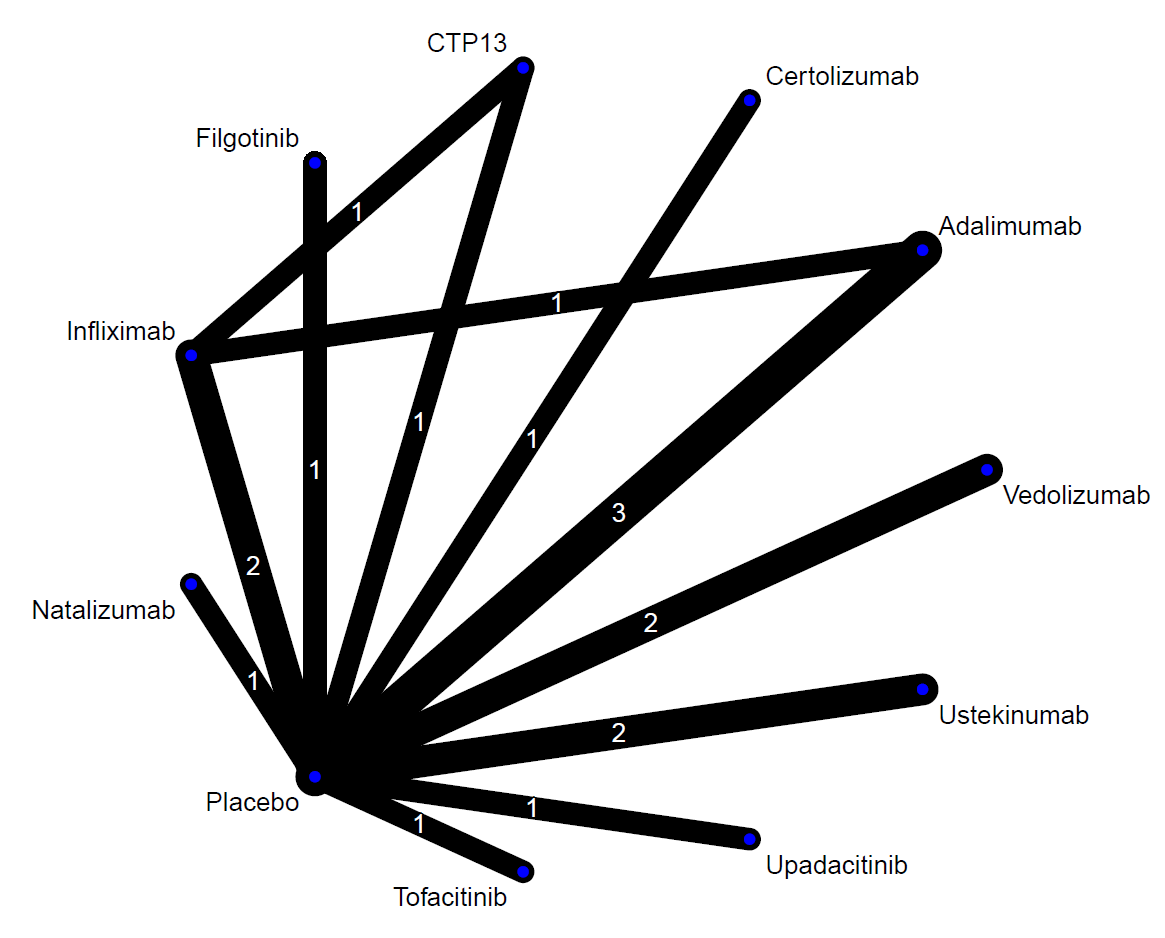


**Network plot for Withdrawal due to adverse events**


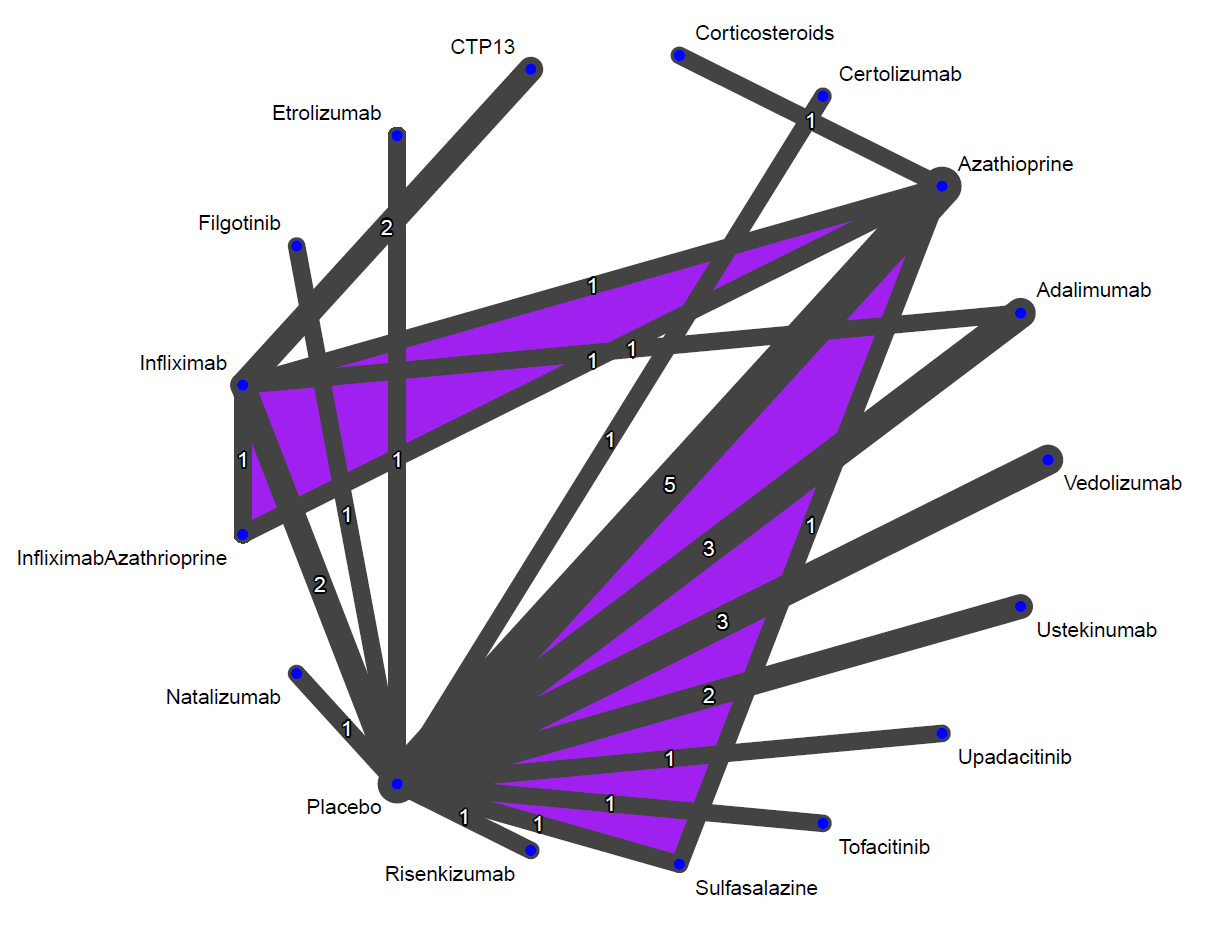


**Network plot for Serious adverse events**


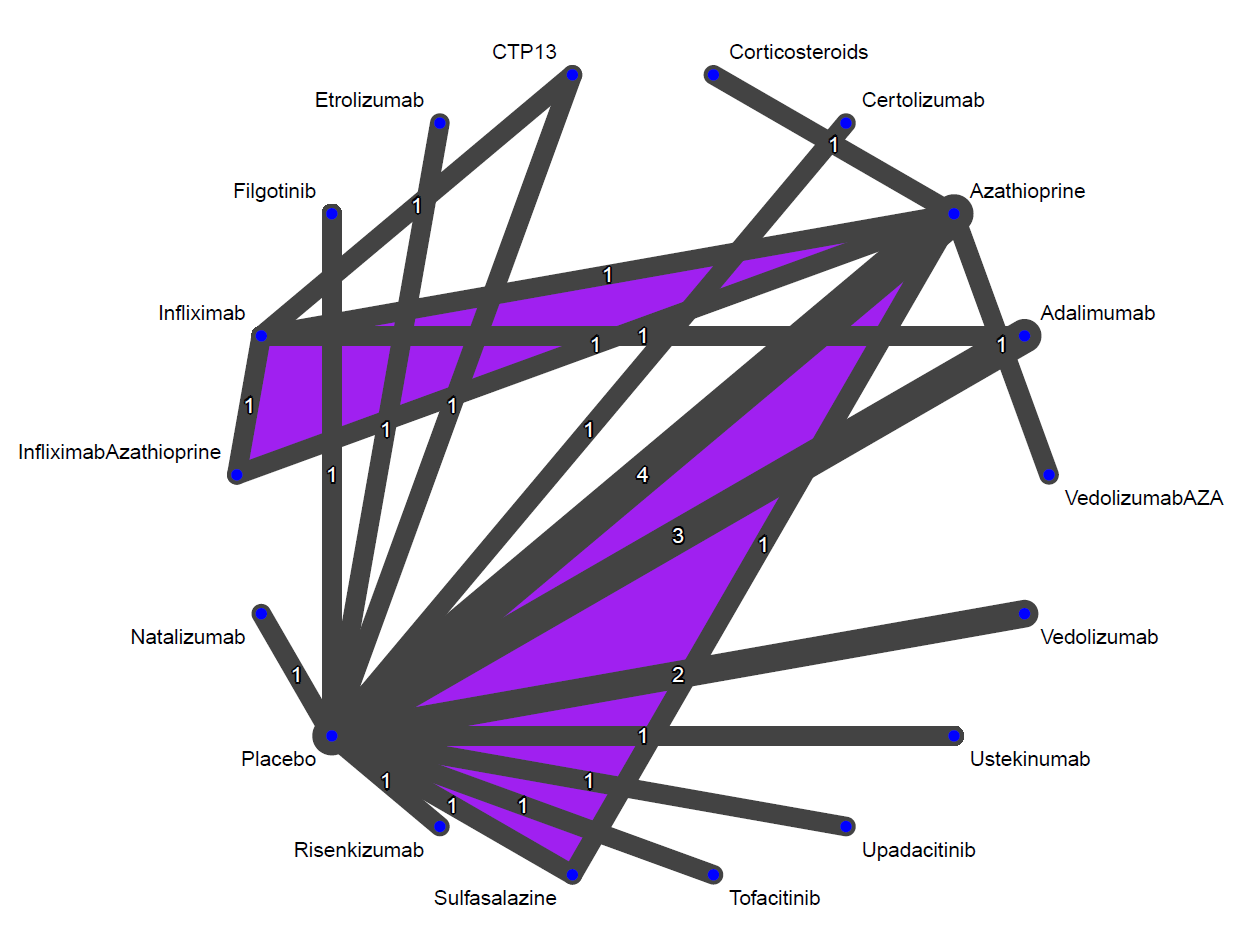


**Network plot for Total adverse events**


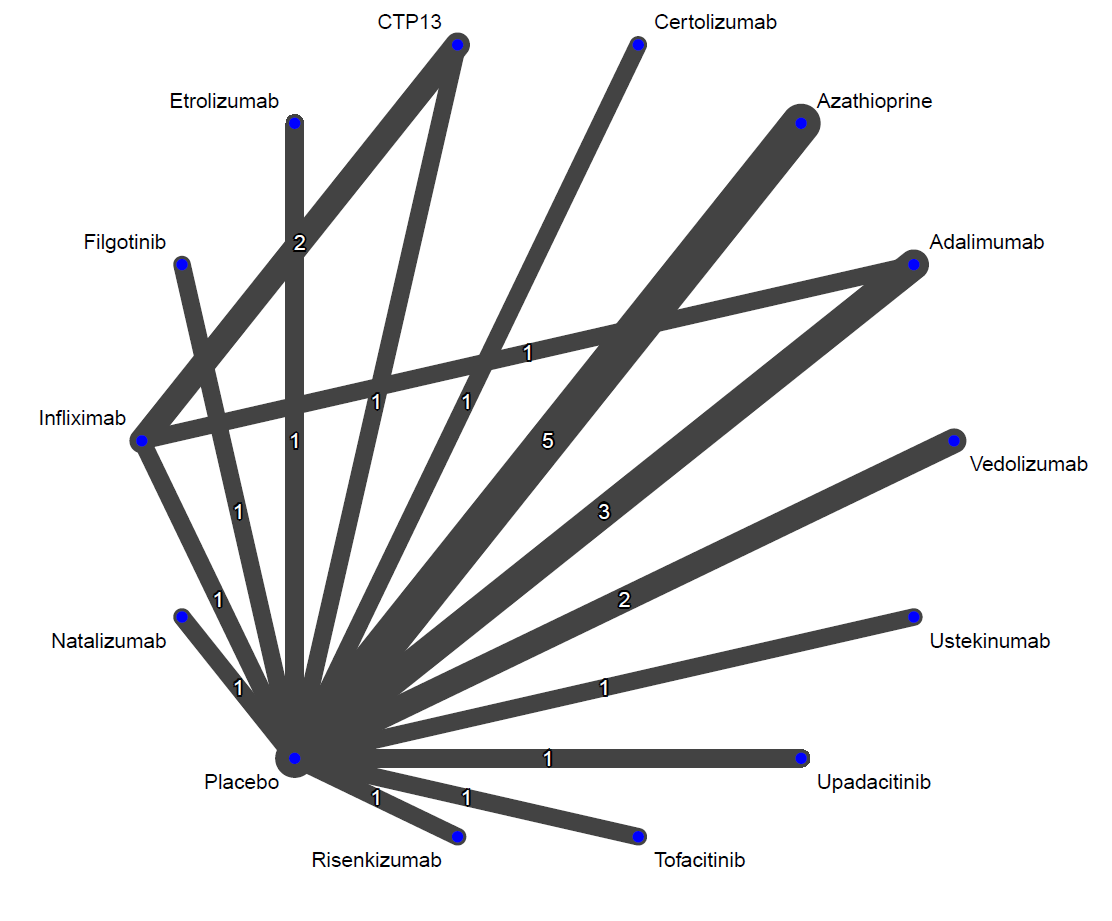


**eFigures 3.** **NETWORK FOREST PLOTS, SUCRA PROBABILITIES, AND DIRECT/INDIRECT/NETWORK ESTIMATES FOREST PLOTS**

**Network forest plot for Clinical Relapse**


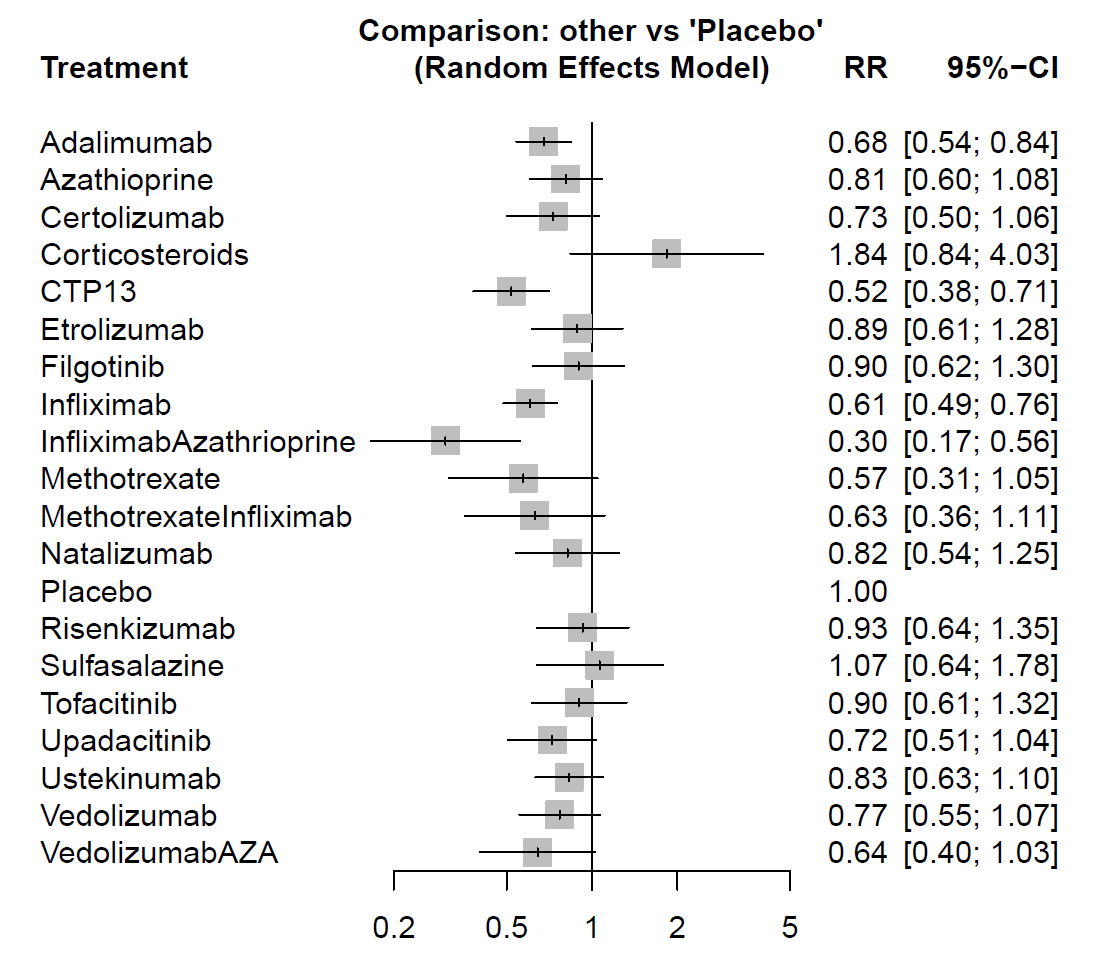


**SUCRA probabilities for Clinical Relapse**


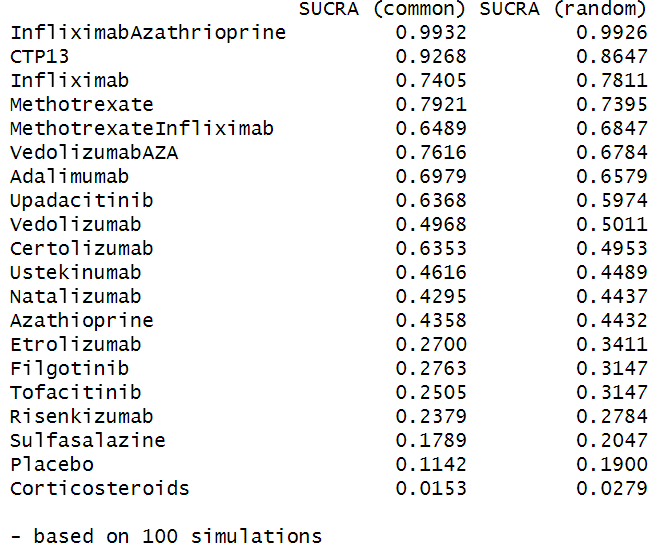


**Direct/indirect/network estimates forest plots for Clinical Relapse**

**
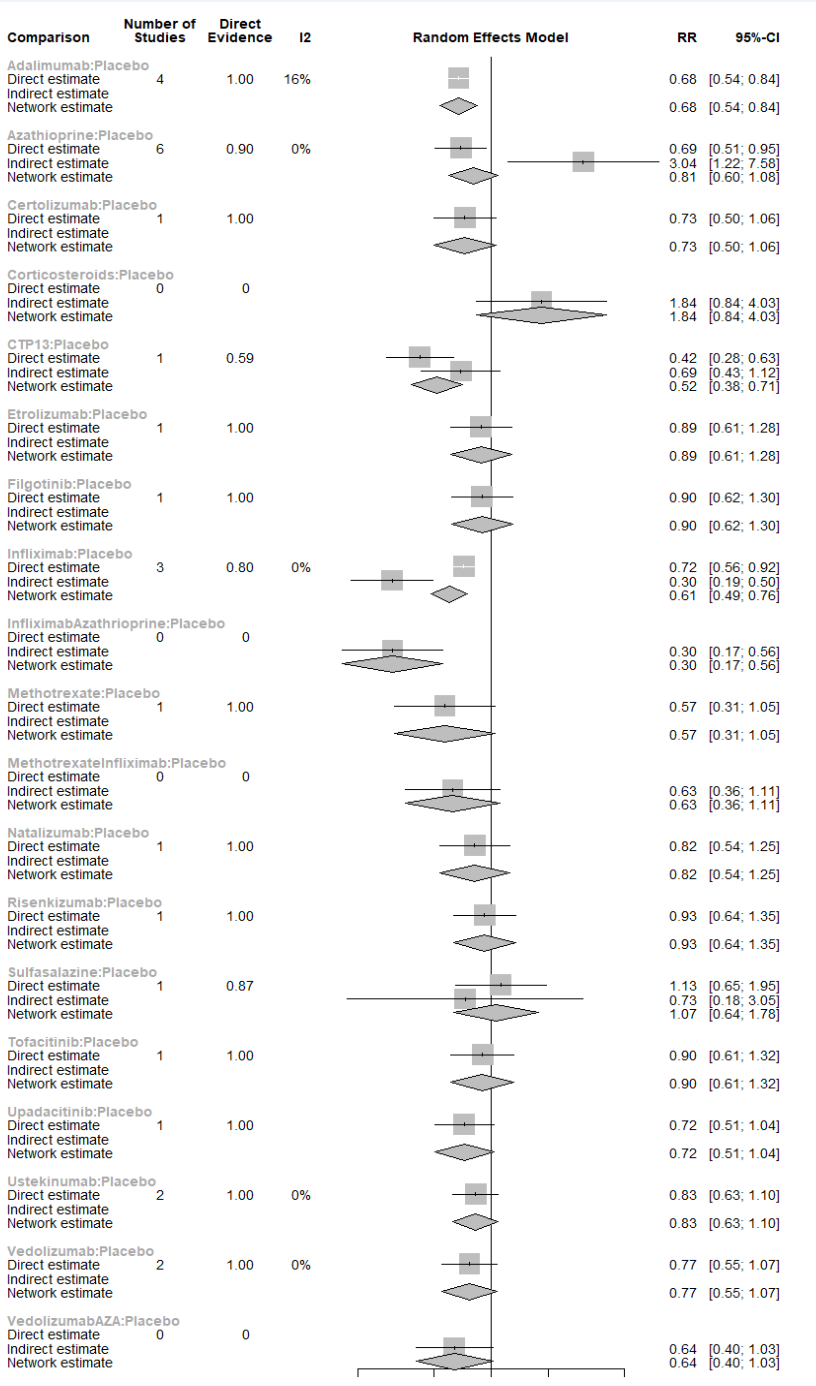
**

**Network forest plots for Clinical Loss of Response**


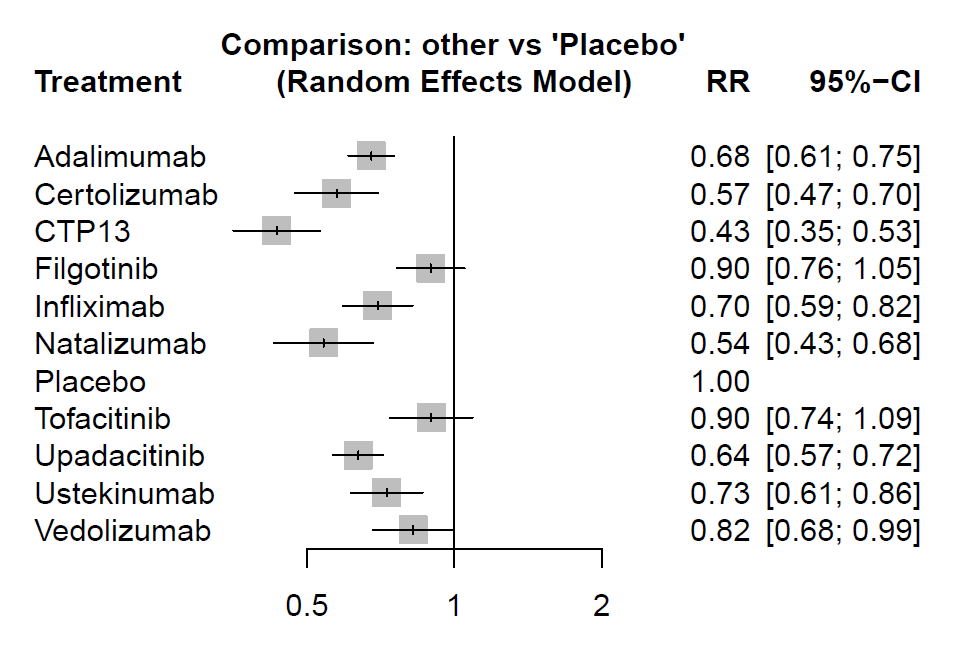


**SUCRA probabilities for Clinical Loss of Response**


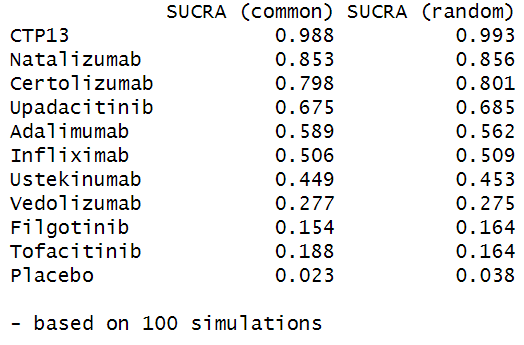


**Direct/indirect/network estimates forest plots for Clinical Loss of Response**


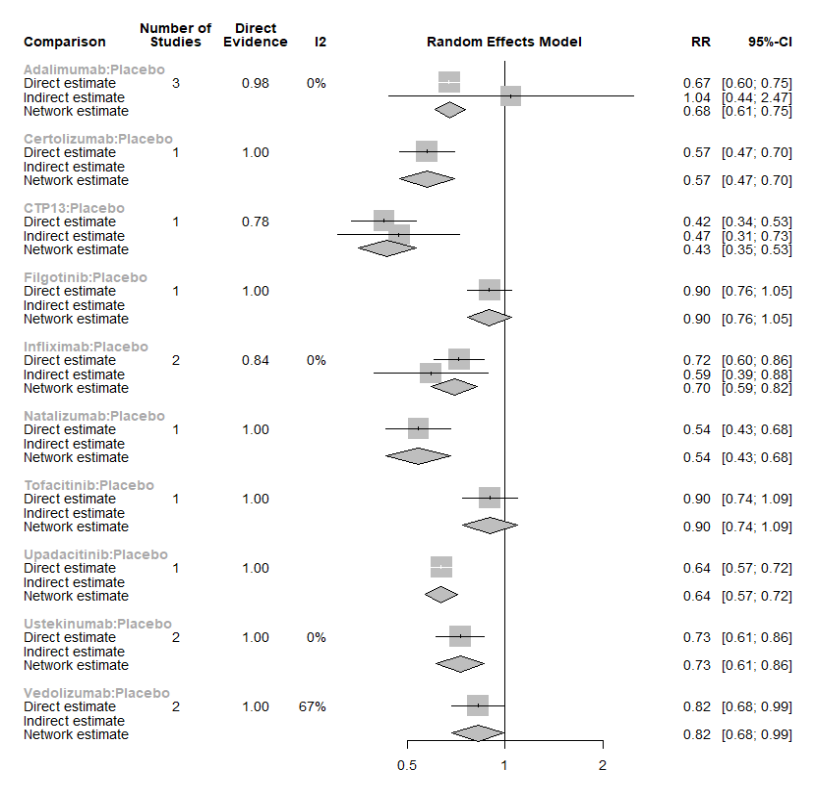


**Network forest plots for Withdrawals due to adverse events**


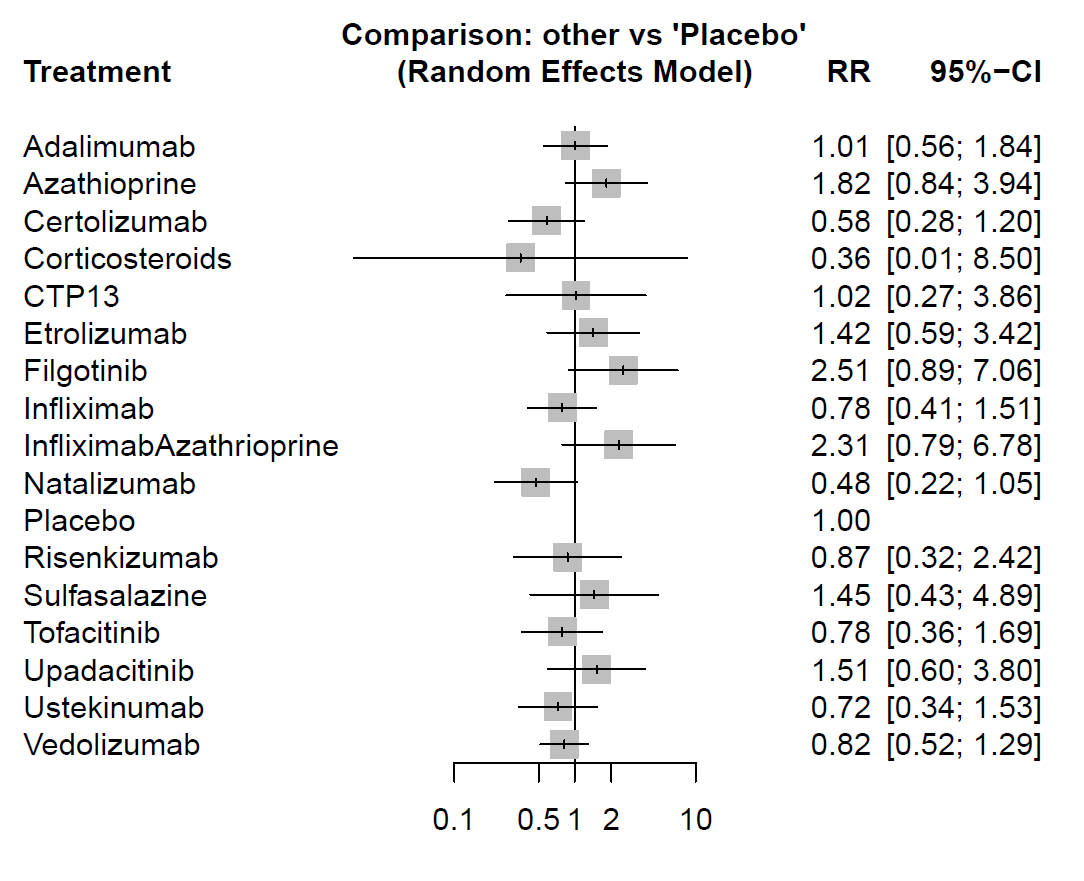


**SUCRA probabilities for Withdrawals due to adverse events**


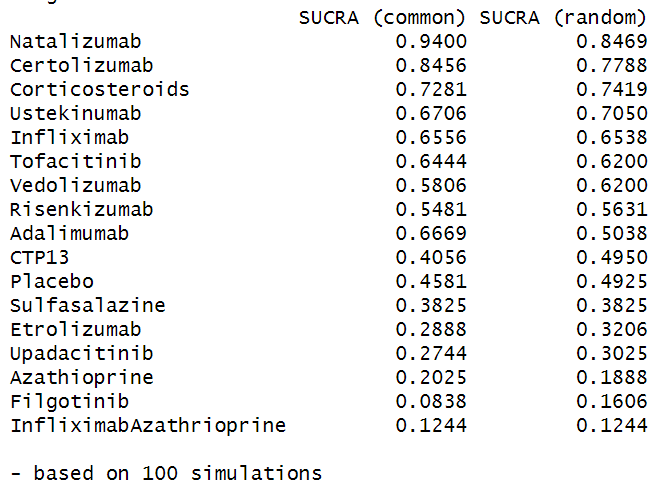


**Direct/indirect/network estimates forest plots for Withdrawals due to adverse events**

**
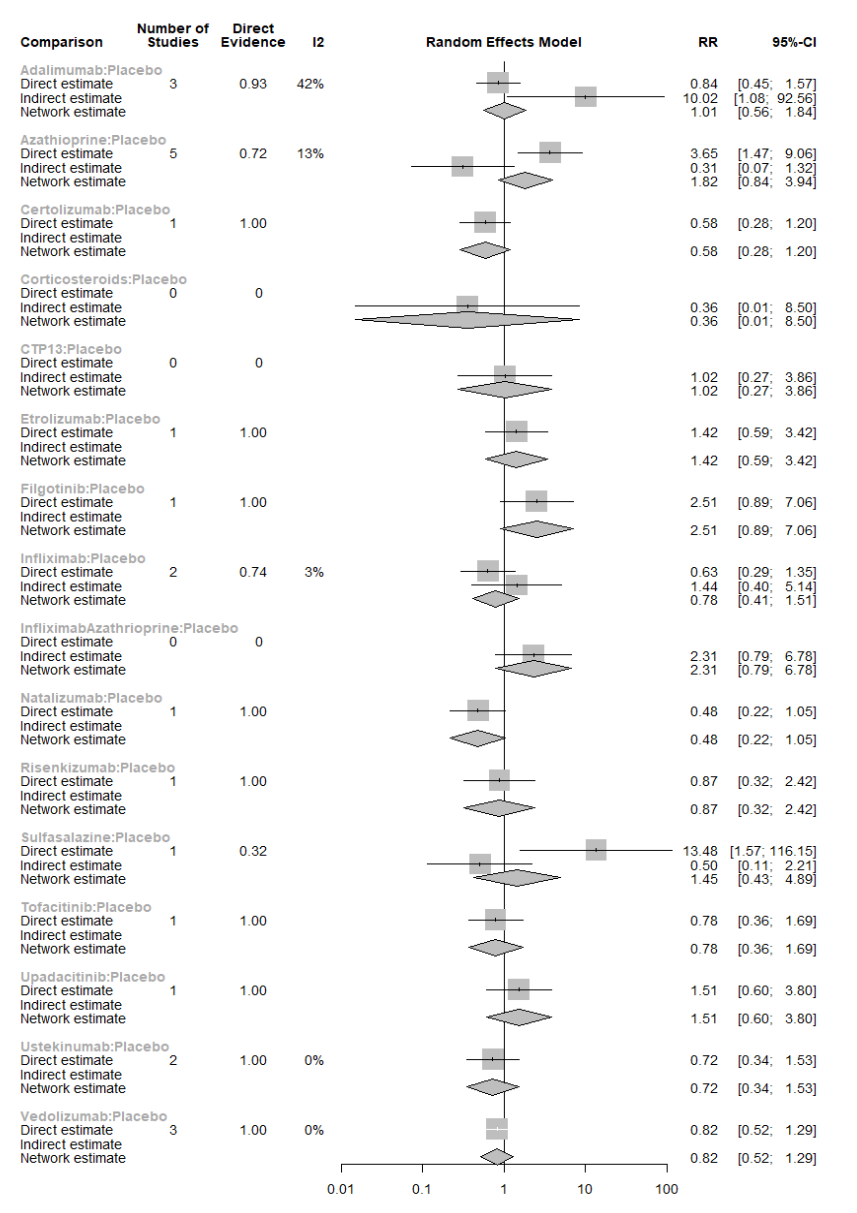
**

**Network forest plots for Serious Adverse Events**


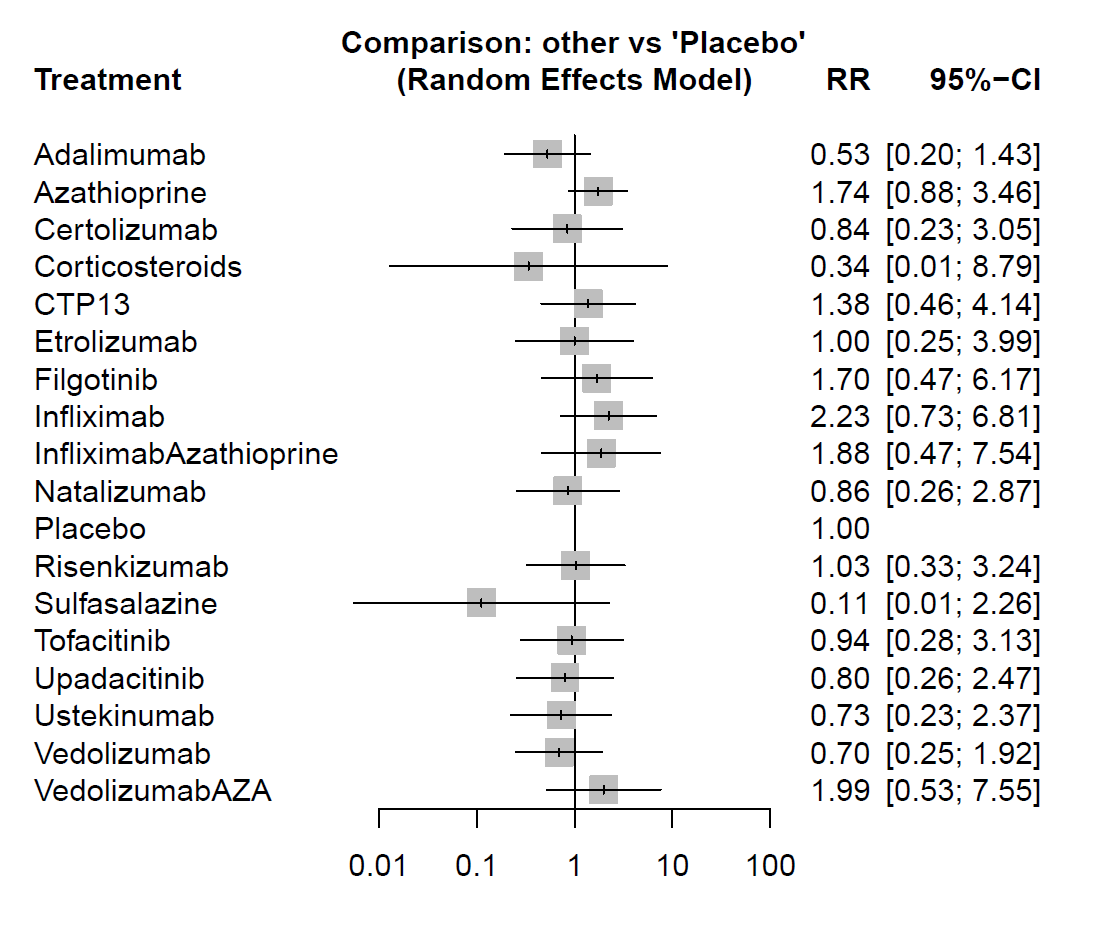


**SUCRA probabilities for Serious Adverse Events**


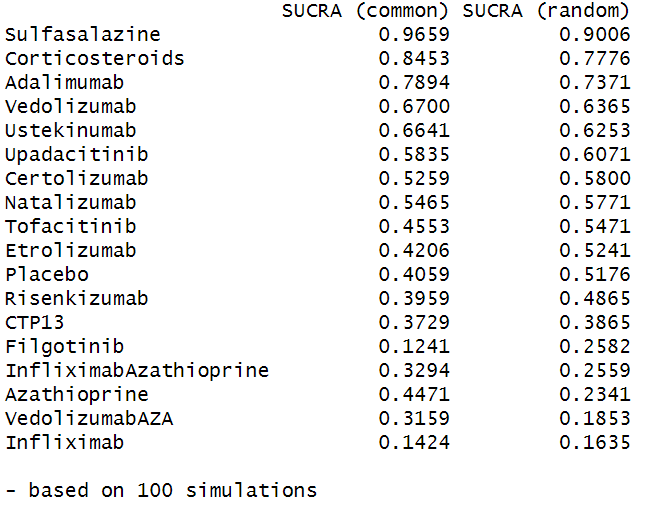


**Direct/indirect/network estimates forest plots for Serious Adverse Events**

**
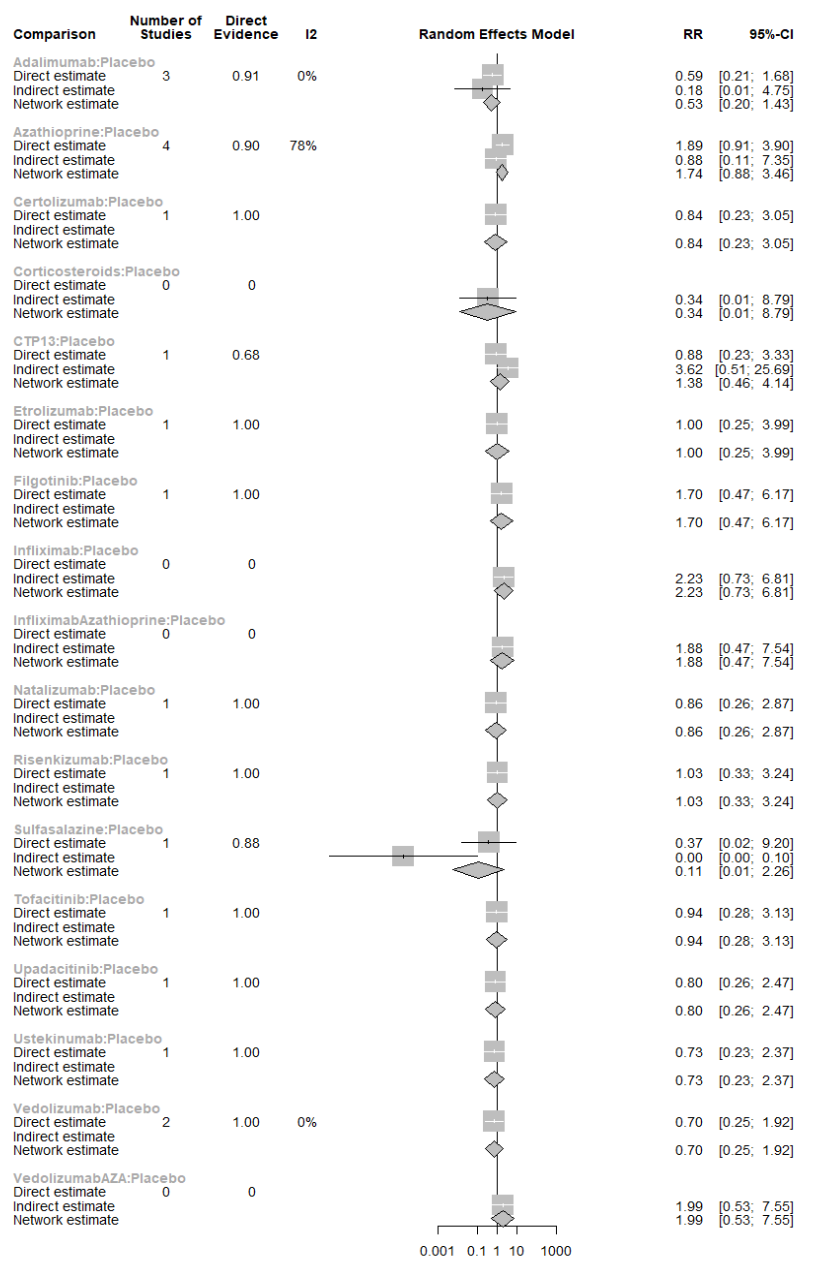
**

**Network forest plots for Total Adverse Events**


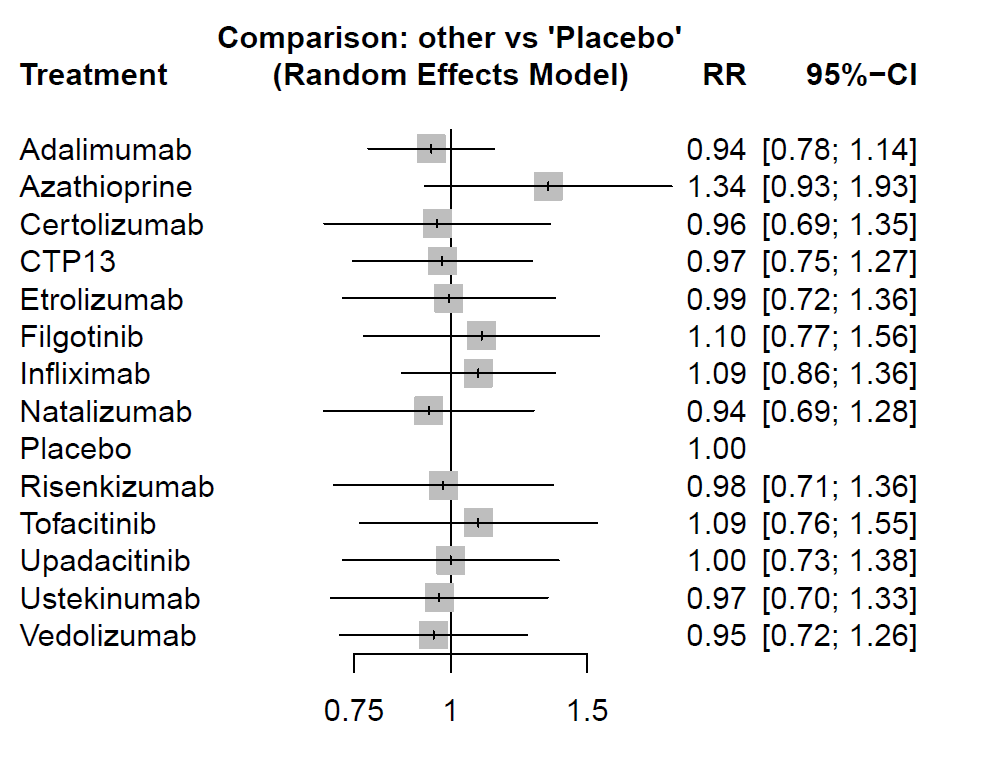


**SUCRA probabilities for Total Adverse Events**


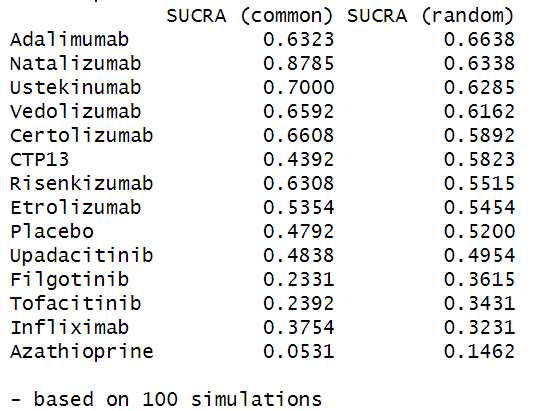


**Direct/indirect/network estimates forest plots for Total Adverse Events**


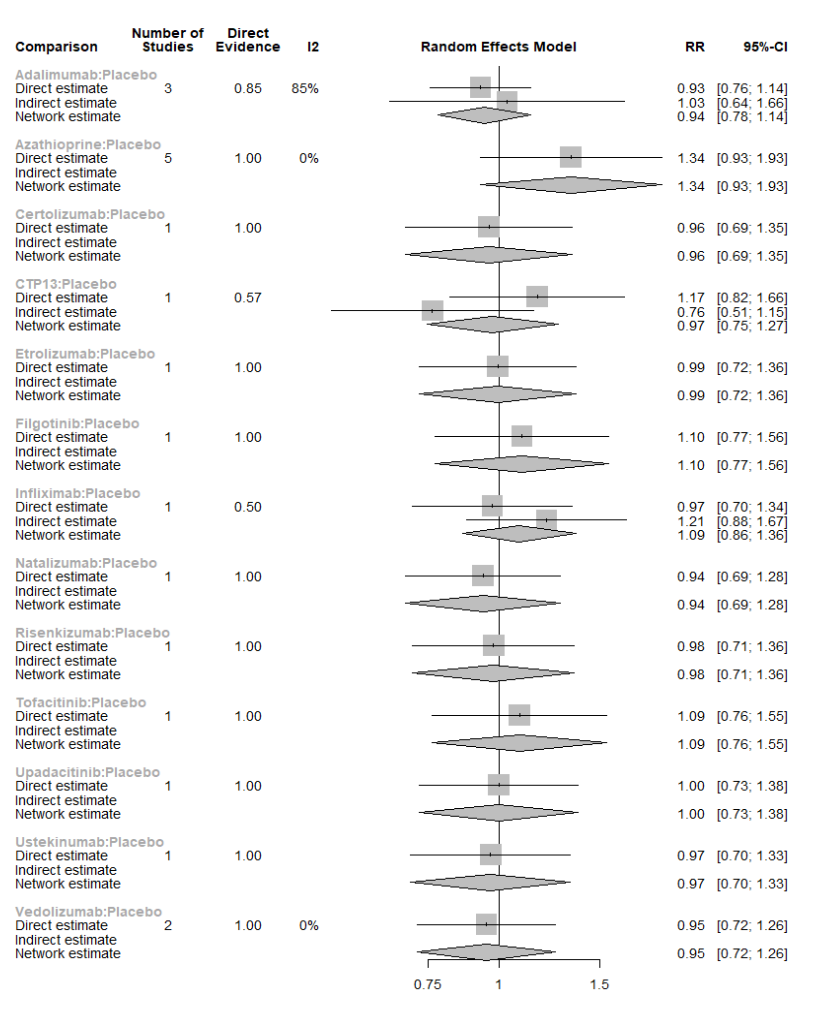


**eFigures 4. SUBGROUP AND SENSITIVITY ANALYSES**

**Clinical relapse – sensitivity analysis for advanced treatment studies only (studies on purine analogues and methotrexate removed, if not compared to advanced treatments)**


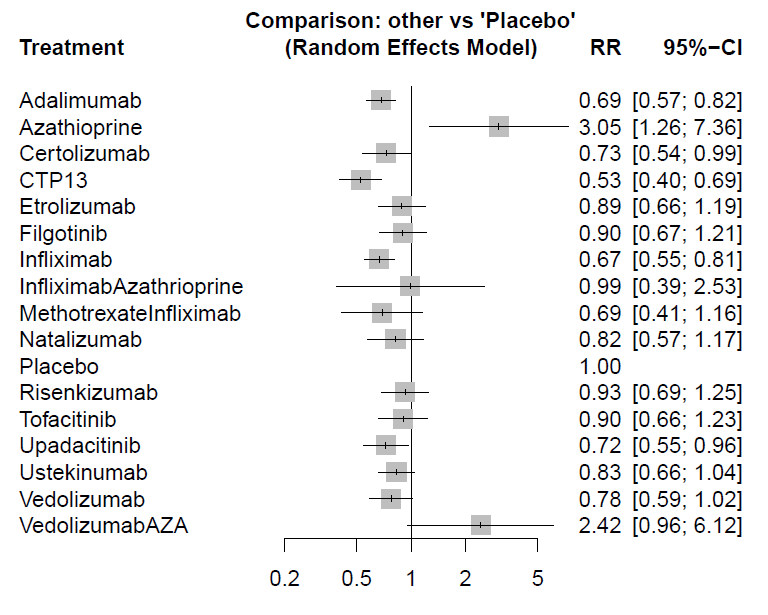


**Clinical relapse -sensitivity analysis for studies taking place in the ‘biologic era’ (studies from 2003 and prior removed)**


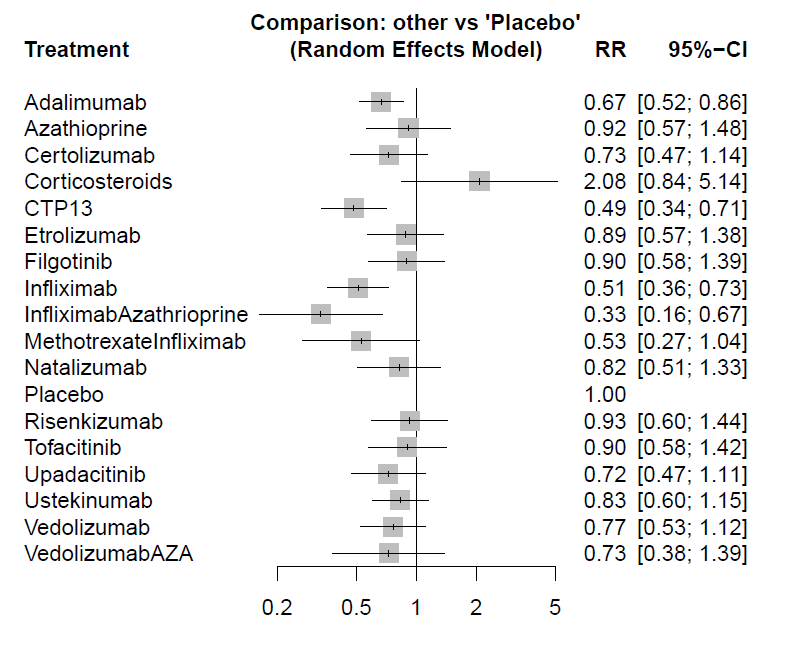


**Clinical relapse – sensitivity analysis for studies of more than 50 participants (studies of 50 participants or less removed)**


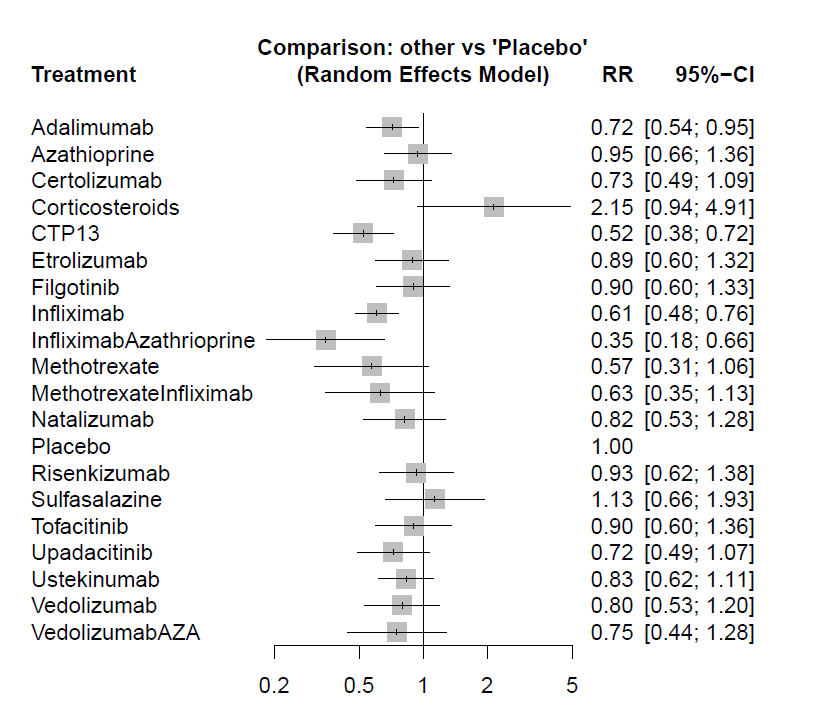


**eFigures 5. Comparison adjusted funnel plots for the assessment of small study effects**

**Clinical relapse**

**
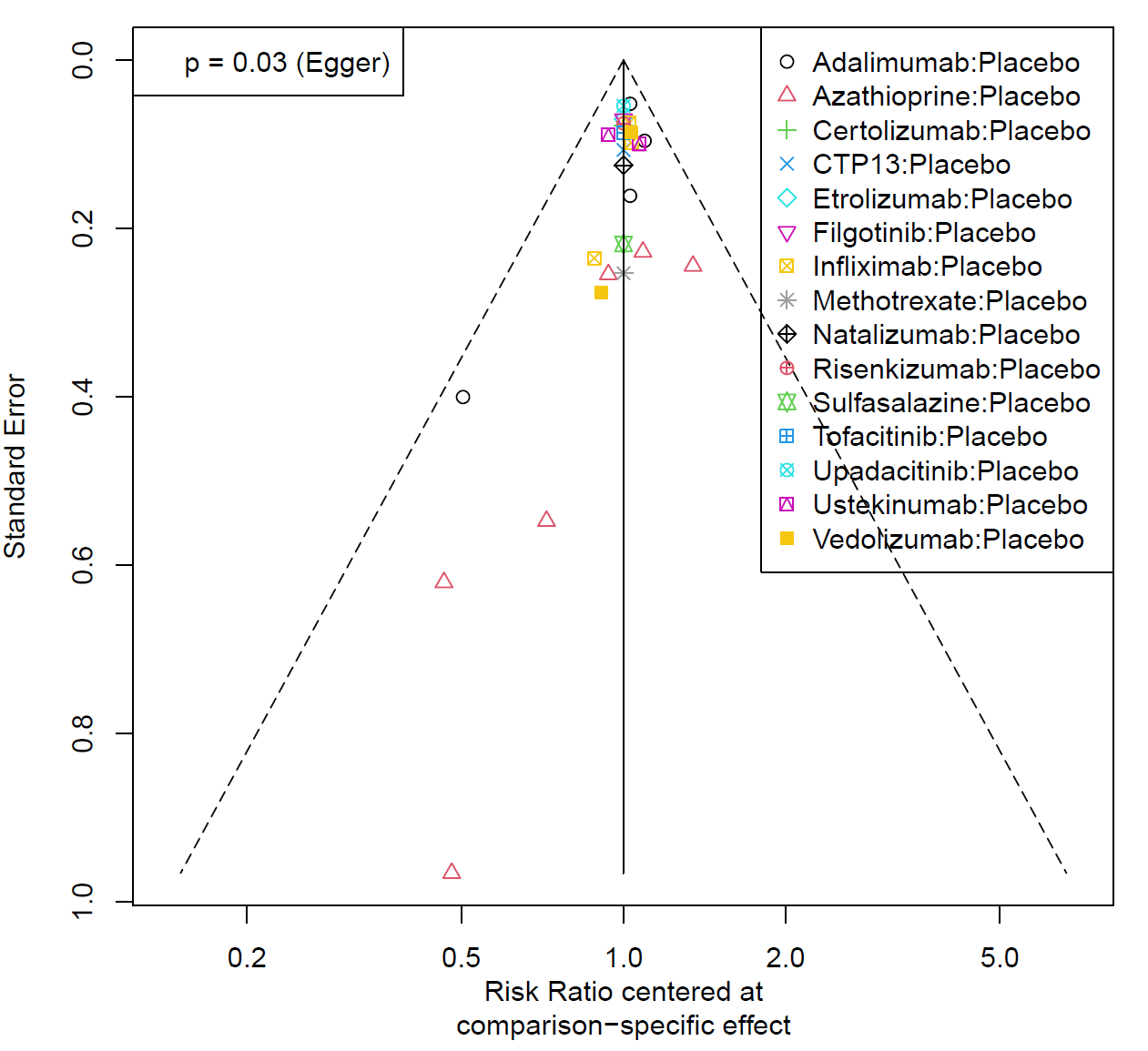
**

**Clinical relapse without studies of 50 participants or less**

**
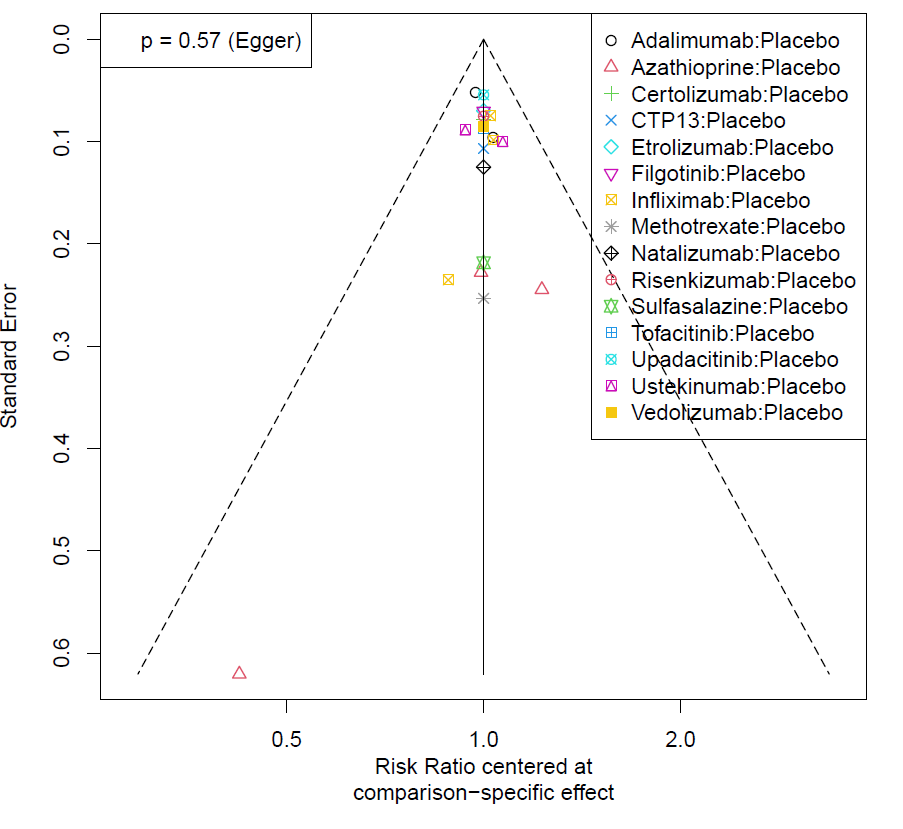
**

**Loss of clinical response**

**
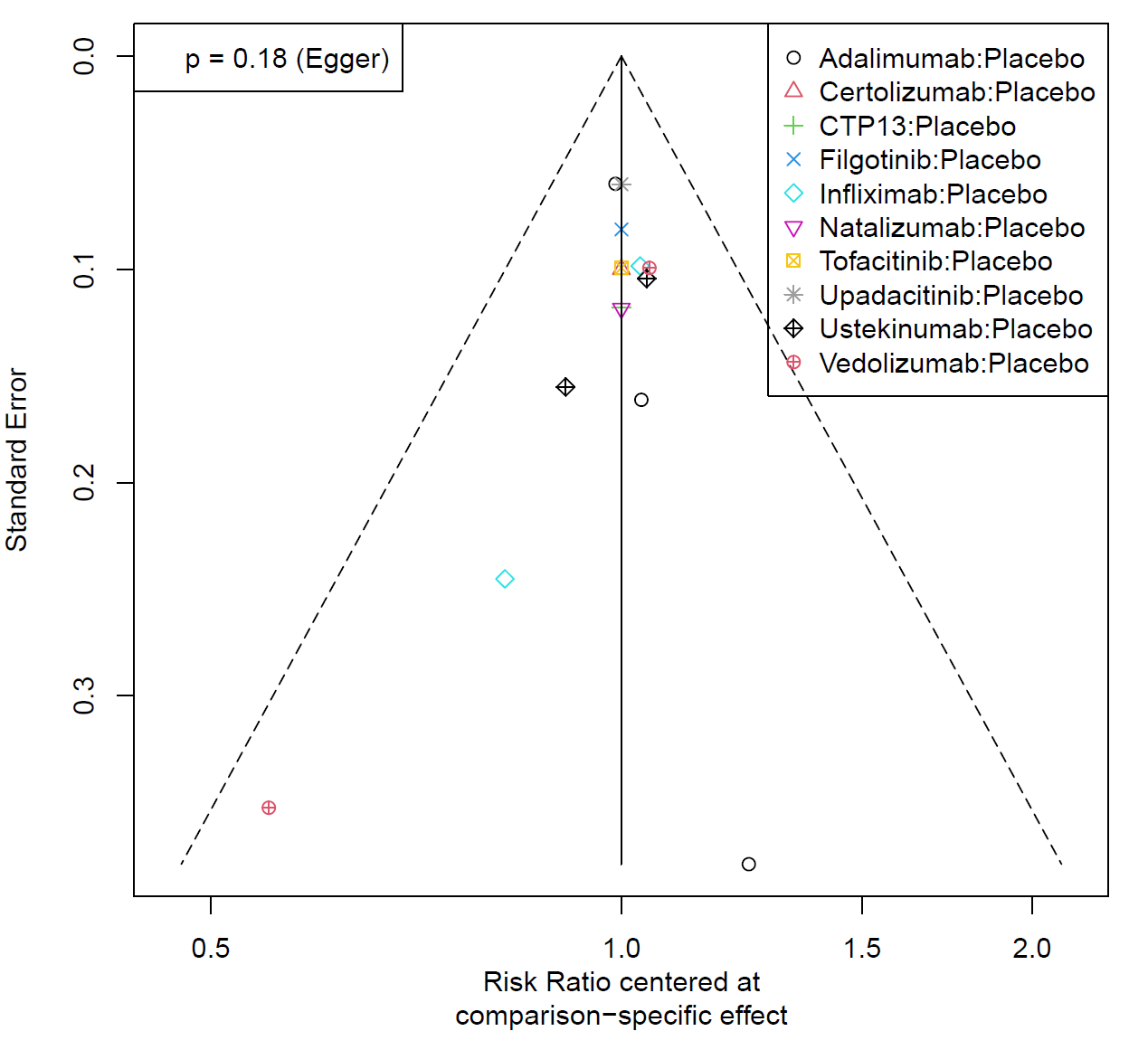
**

**Withdrawals due to adverse events**

**
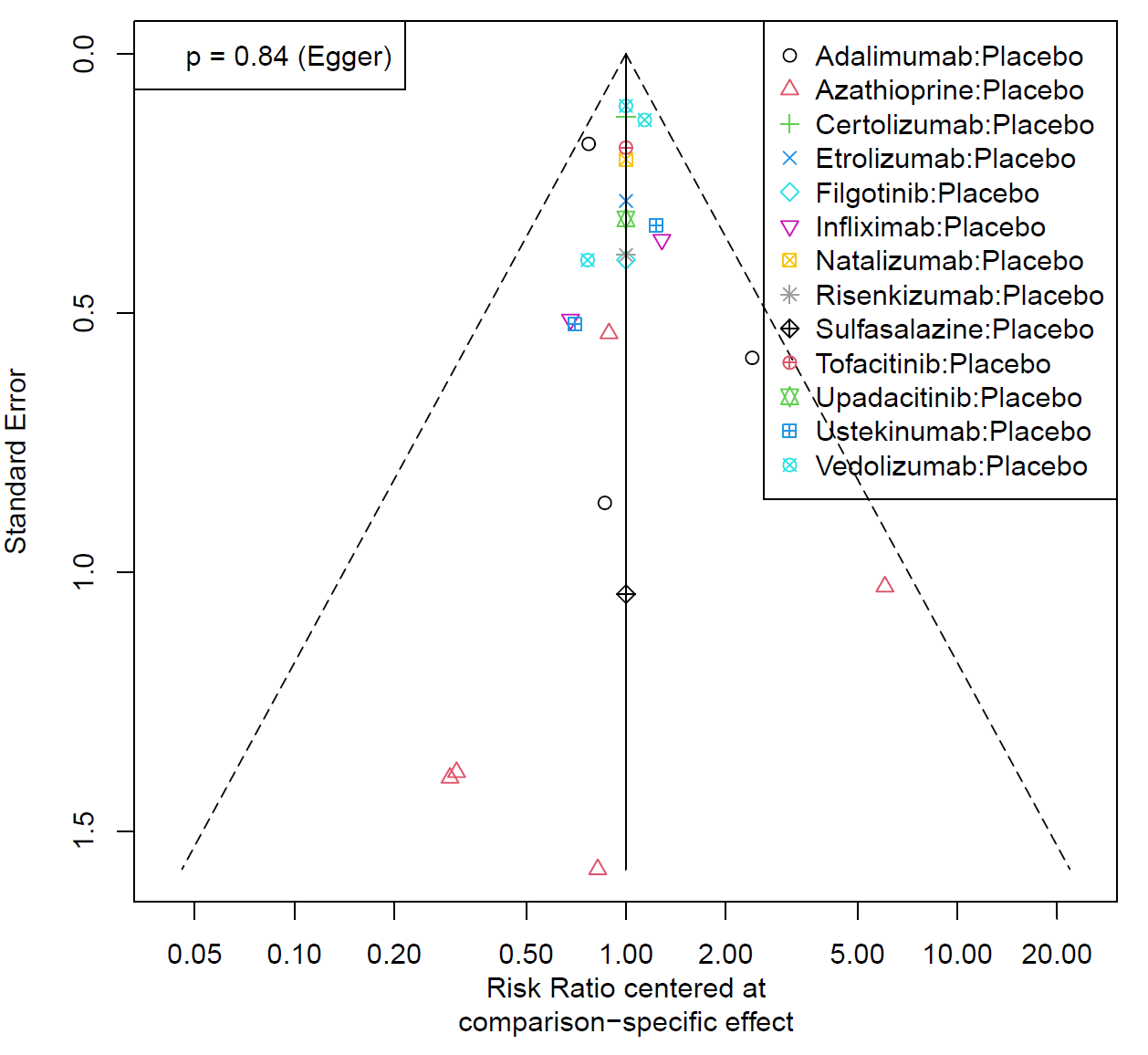
**

**Serious adverse events**

**
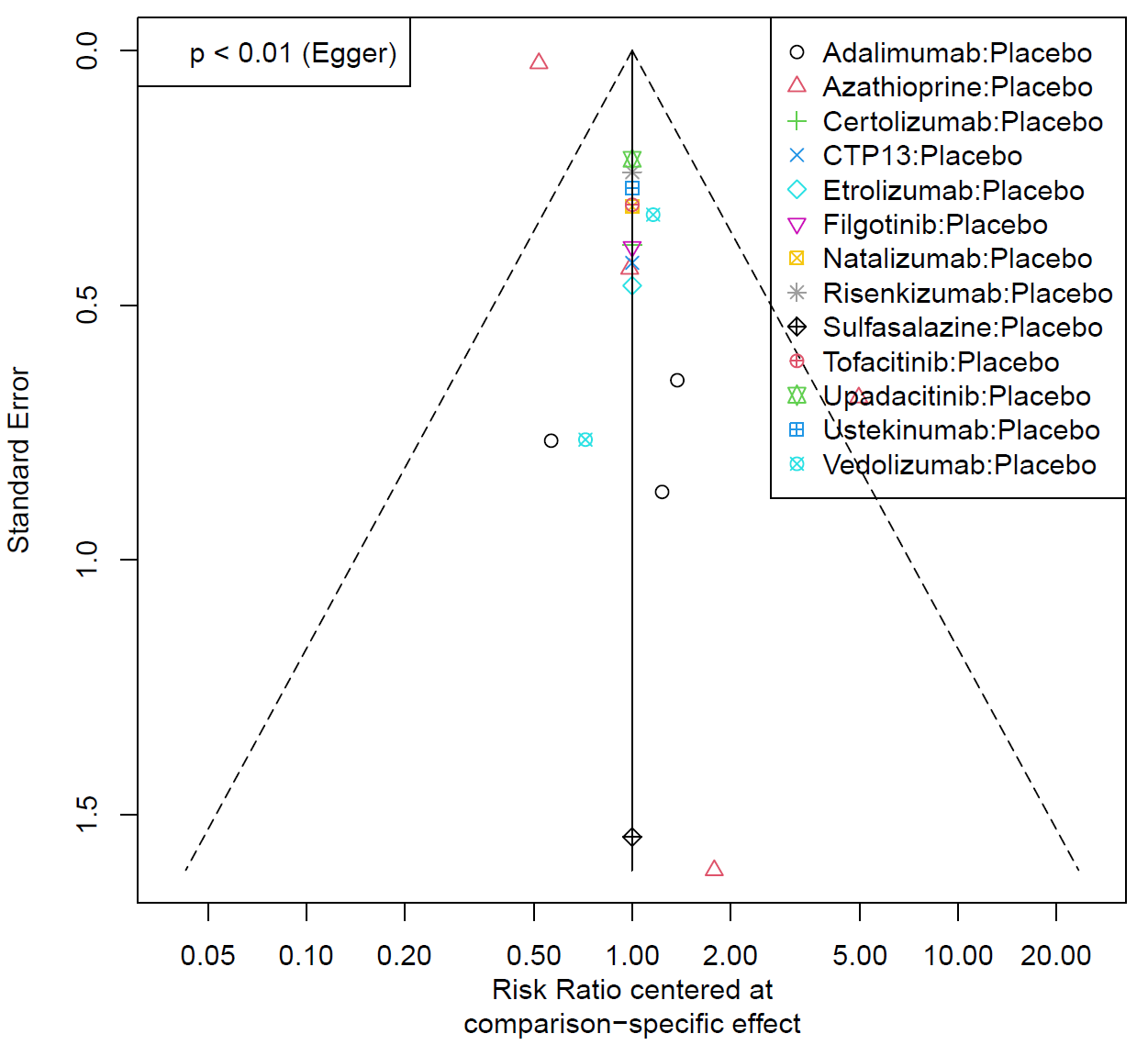
**

**Total adverse events**

**
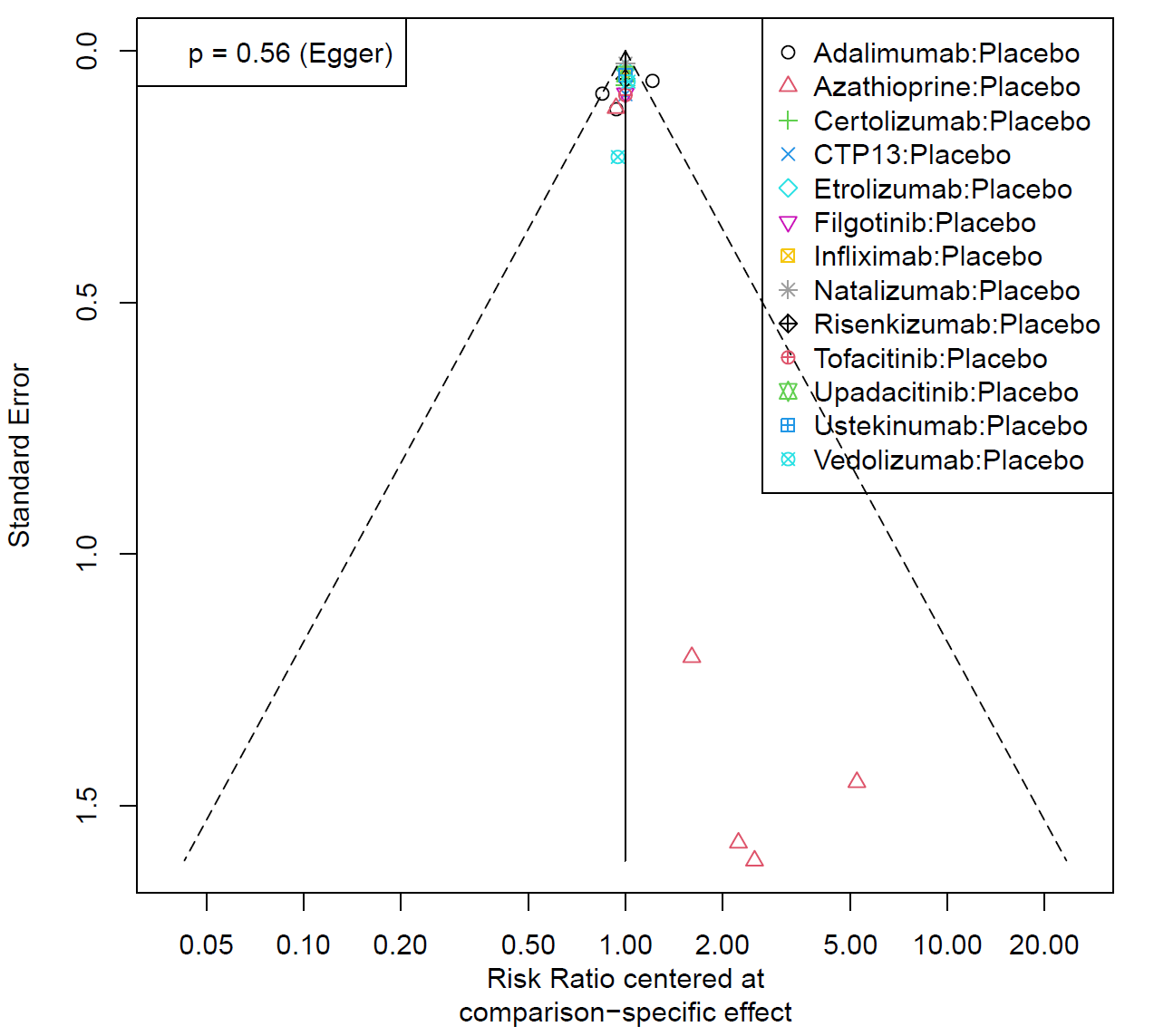
**

**eAppendix 1**. Search strategy

CENTRAL via Cochrane Library

Date Run: 11/04/2022 18:26:28

#1 [mh "Inflammatory Bowel Diseases"] or [mh "Colitis, Ulcerative"] or [mh "Crohn Disease"] or ("Inflammatory Bowel Disease" or "Inflammatory Bowel Diseases" or Crohn* or Colitis or Enteritis or Proctocolitis" or Ileocolitis or Enterocolitis or Ileitides or Ileitis or Colorectitis"):ti,ab with Cochrane Library publication date from Jan 2018 to present, in Trials 6923

#2 (clinicaltrials* or trialsearch*):so 397441

#3 #1 NOT #2 3743

MEDLINE via Ovid SP

Database: Ovid MEDLINE(R) ALL <1946 to April 08, 2022>

1 exp Inflammatory Bowel Diseases/ or Colitis, Ulcerative/ or Crohn Disease/ or ("Inflammatory Bowel Disease" or "Inflammatory Bowel Diseases" or Crohn* or Colitis or Enteritis or Proctocolitis or Ileocolitis or Enterocolitis or Ileitides or Ileitis or Colorectitis).ti,ab. (167093)

2 ((Randomized Controlled Trial or Controlled Clinical Trial).pt. or (Randomi?ed or Placebo or Randomly or Trial or Groups).ab. or Drug Therapy.fs.) not (exp Animals/ not Humans.sh.) (4641476)

3 1 and 2 (43216)

4 limit 3 to ed=20180101-20221231 (10906)

5 limit 3 to dt=20180101-20221231 (10714)

6 4 or 5 (12373)

Embase via Ovid SP

Database: Embase <1974 to 2022 April 08>

1 Randomized controlled trial/ or Controlled clinical study/ or randomization/ or intermethod comparison/ or double blind procedure/ or human experiment/ or (random$ or placebo or (open adj label) or ((double or single or doubly or singly) adj (blind or blinded or blindly)) or parallel group$1 or crossover or cross over or ((assign$ or match or matched or allocation) adj5 (alternate or group$1 or intervention$1 or patient$1 or subject$1 or participant$1)) or assigned or allocated or (controlled adj7 (study or design or trial)) or volunteer or volunteers).ti,ab. or (compare or compared or comparison or trial).ti. or ((evaluated or evaluate or evaluating or assessed or assess) and (compare or compared or comparing or comparison)).ab. (5728242)

2 (random$ adj sampl$ adj7 ("cross section$" or questionnaire$1 or survey$ or database$1)).ti,ab. not (comparative study/ or controlled study/ or randomi?ed controlled.ti,ab. or randomly assigned.ti,ab.) (8944)

3 Cross-sectional study/ not (randomized controlled trial/ or controlled clinical study/ or controlled study/ or (randomi?ed controlled or control group$1).ti,ab.) (305385)

4 (((case adj control$) and random$) not randomi?ed controlled).ti,ab. (19659)

5 (Systematic review not (trial or study)).ti. (206139)

6 (nonrandom$ not random$).ti,ab. (17705)

7 ("Random field$" or (random cluster adj3 sampl$)).ti,ab. (4107)

8 (review.ab. and review.pt.) not trial.ti. (982757)

9 "we searched".ab. and (review.ti. or review.pt.) (41335)

10 ("update review" or (databases adj4 searched)).ab. (49993)

11 (rat or rats or mouse or mice or swine or porcine or murine or sheep or lambs or pigs or piglets or rabbit or rabbits or cat or cats or dog or dogs or cattle or bovine or monkey or monkeys or trout or marmoset$1).ti. and animal experiment/ (1145284)

12 Animal experiment/ not (human experiment/ or human/) (2404090)

13 or/2-12 (3934547)

14 1 not 13 (5075178)

15 Inflammatory Bowel Disease/ or exp Crohn Disease/ or Ulcerative Colitis/ or Acute Severe Ulcerative Colitis/ or ("Inflammatory Bowel Disease" or "Inflammatory Bowel Diseases" or Crohn* or Colitis or Enteritis or Proctocolitis or Ileocolitis or Enterocolitis or Ileitides or Ileitis or Colorectitis).ti,ab. (263786)

16 14 and 15 (45879)

17 limit 16 to dc=20180101-20221231 (15753)

18 limit 16 to dd=20180101-20221231 (8910)

19 17 or 18 (15770)

20 limit 19 to embase (5793)

Science Citation Index-Expanded via Web of Science Core Collection

#3 #1 AND #2 Editions: Science Citation Index Expanded (SCI-EXPANDED)--10:49 PM | Timespan: 2018-01-01 to 2022-12-31 (Publication Date) 4,447

#2 "Inflammatory Bowel Disease" or "Inflammatory Bowel Diseases" or Crohn* or Colitis or Enteritis or Proctocolitis or Ileocolitis or Enterocolitis or Ileitides or Ileitis or Colorectitis (Title) or "Inflammatory Bowel Disease" or "Inflammatory Bowel Diseases" or Crohn* or Colitis or Enteritis or Proctocolitis or Ileocolitis or Enterocolitis or Ileitides or Ileitis or Colorectitis (Abstract) 177,968

#1 Random* OR Blind* OR Allocat* OR Assign* OR Trial* OR Placebo* OR Crossover* OR Cross-Over* (Title) or Random* OR Blind* OR Allocat* OR Assign* OR Trial* OR Placebo* OR Crossover* OR Cross-Over* (Abstract) 2,995,649

**eAppendix 2.** References of included studies

1. Buhl S, Steenholdt C, Brynskov J, et al. Discontinuation of Infliximab Therapy in Patients with Crohn's Disease. NEJM Evid. 2022;1(8):EVIDoa2200061. Epub 2022/08/01.

2. Colombel JF, Sandborn WJ, Rutgeerts P, et al. Adalimumab for maintenance of clinical response and remission in patients with Crohn's disease: the CHARM trial. Gastroenterology. 2007;132(1):52-65. Epub 2007/01/24.

3. Feagan BG, Fedorak RN, Irvine EJ, et al. A comparison of methotrexate with placebo for the maintenance of remission in Crohn's disease. North American Crohn's Study Group Investigators. N Engl J Med. 2000;342(22):1627-32. Epub 2000/06/01.

4. Feagan BG, McDonald JW, Panaccione R, et al. Methotrexate in combination with infliximab is no more effective than infliximab alone in patients with Crohn's disease. Gastroenterology. 2014;146(3):681-8.e1. Epub 2013/11/26.

5. Feagan BG, Sandborn WJ, Gasink C, et al. Ustekinumab as Induction and Maintenance Therapy for Crohn's Disease. N Engl J Med. 2016;375(20):1946-60. Epub 2016/12/14.

6. Ferrante M, Panaccione R, Baert F, et al. Risankizumab as maintenance therapy for moderately to severely active Crohn's disease: results from the multicentre, randomised, double-blind, placebo-controlled, withdrawal phase 3 FORTIFY maintenance trial. Lancet. 2022;399(10340):2031-46. Epub 2022/06/02.

7. Hanauer S, Feagan BG, Lichtenstein GR, et al. Maintenance infliximab for Crohn’s disease: the ACCENT I randomised trial. The Lancet. 2002;359.

8. Hanauer SB, Sands BE, Schreiber S, Danese S, Kłopocka M, Kierkuś J, Kulynych R, Gonciarz M, Sołtysiak A, Smoliński P, Srećković S. Subcutaneous infliximab (CT-P13 SC) as maintenance therapy for inflammatory bowel disease: two randomized phase 3 trials (LIBERTY). Gastroenterology. 2024 Oct 1;167(5):919-33.

9. Jorgensen KK, Olsen IC, Goll GL, et al. Switching from originator infliximab to biosimilar CT-P13 compared with maintained treatment with originator infliximab (NOR-SWITCH): a 52-week, randomised, double-blind, non-inferiority trial. Lancet. 2017;389(10086):2304-16. Epub 2017/05/16.

10. Lemann M, Mary JY, Colombel JF, et al. A randomized, double-blind, controlled withdrawal trial in Crohn's disease patients in long-term remission on azathioprine. Gastroenterology. 2005;128(7):1812-8. Epub 2005/06/09.

11. Loftus EV, Jr., Panes J, Lacerda AP, et al. Upadacitinib Induction and Maintenance Therapy for Crohn's Disease. N Engl J Med. 2023;388(21):1966-80. Epub 2023/05/24.

12. Louis E, Resche-Rigon M, Laharie D, et al. Withdrawal of infliximab or concomitant immunosuppressant therapy in patients with Crohn's disease on combination therapy (SPARE): a multicentre, open-label, randomised controlled trial. Lancet Gastroenterol Hepatol. 2023;8(3):215-27. Epub 2023/01/15.

13. Mantzaris GJ, Christidou A, Sfakianakis M, et al. Azathioprine is superior to budesonide in achieving and maintaining mucosal healing and histologic remission in steroid-dependent Crohn's disease. Inflamm Bowel Dis. 2009;15(3):375-82. Epub 2008/11/15.

14. O'Donoghue D, Dawson A, Powell-Tuck J, et al. Double-blind withdrawal trial of azathioprine as maintenance treatment for Crohn's disease. The Lancet. 1978;312(8097):955-7.

15. Panes J, Lopez-Sanroman A, Bermejo F, et al. Early azathioprine therapy is no more effective than placebo for newly diagnosed Crohn's disease. Gastroenterology. 2013;145(4):766-74 e1. Epub 2013/06/19.

16. Panes J, Sandborn WJ, Schreiber S, et al. Tofacitinib for induction and maintenance therapy of Crohn's disease: results of two phase IIb randomised placebo-controlled trials. Gut. 2017;66(6):1049-59. Epub 2017/02/18.

17. Röder H. Kontrollierte Einführung des Infliximab-Biosimilars CT-P13 bei Patienten mit Morbus Crohn und Colitis ulcerosa in einer großen deutschen Zentrumskohorte: lmu; 2021.

18. Rosenberg JL, Levin B, Wall AJ, Kirsner JB. A controlled trial of azathioprine in Crohn's disease. The American Journal of Digestive Diseases. 1975;20(8):721-6.

19. Rutgeerts P, D’Haens G, Targan S, et al. Efficacy and Safety of Retreatment With Anti–Tumor Necrosis Factor Antibody (Infliximab) to Maintain Remission in Crohn’s Disease. GASTROENTEROLOGY. 1999;117:761–9.

20. Rutgeerts P, Van Assche G, Sandborn WJ, et al. Adalimumab induces and maintains mucosal healing in patients with Crohn's disease: data from the EXTEND trial. Gastroenterology. 2012;142(5):1102-11 e2. Epub 2012/02/14.

21. Sandborn WJ, Colombel JF, Enns R, et al. Natalizumab Induction and Maintenance Therapy for Crohn’s Disease. N engl j med. 2005;353(18):1913-25.

22. Sandborn WJ, Hanauer SB, Rutgeerts P, et al. Adalimumab for maintenance treatment of Crohn's disease: results of the CLASSIC II trial. Gut. 2007;56(9):1232-9. Epub 2007/02/15.

23. Sandborn WJ, Gasink C, Gao LL, et al. Ustekinumab induction and maintenance therapy in refractory Crohn's disease. N Engl J Med. 2012;367(16):1519-28. Epub 2012/10/19.

24. Sandborn WJ, Feagan BG, Rutgeerts P, et al. Vedolizumab as induction and maintenance therapy for Crohn's disease. N Engl J Med. 2013;369(8):711-21. Epub 2013/08/24.

25. Sandborn WJ, Panes J, Danese S, et al. Etrolizumab as induction and maintenance therapy in patients with moderately to severely active Crohn's disease (BERGAMOT): a randomised, placebo-controlled, double-blind, phase 3 trial. Lancet Gastroenterol Hepatol. 2023;8(1):43-55. Epub 2022/10/15.

26. Sands BE, Anderson FH, Bernstein CN, et al. Infliximab Maintenance Therapy for Fistulizing Crohn’s Disease. N Engl J Med. 2004;350(9):876-85.

27. Schreiber S, Khaliq-Kareemi M, Lawrance IC, et al. Maintenance Therapy with Certolizumab Pegol for Crohn’s Disease. The New England Journal of Medicine. 2007;357(3):239-50.

28. Summers RW, Switz DM, Sessions JT, Jr., et al. National Cooperative Crohn's Disease Study: results of drug treatment. Gastroenterology. 1979;77(4 Pt 2):847-69. Epub 1979/10/01.

29. Van Assche G, Vermeire S, Ballet V, et al. Switch to adalimumab in patients with Crohn's disease controlled by maintenance infliximab: prospective randomised SWITCH trial. Gut. 2012;61(2):229-34. Epub 2011/09/29.

30. Vermeire S, Schreiber S, Rubin DT, D'Haens G, Reinisch W, Watanabe M, Mehta R, Roblin X, Beales I, Gietka P, Hibi T. Efficacy and safety of filgotinib as induction and maintenance therapy for Crohn's disease (DIVERSITY): a phase 3, double-blind, randomised, placebo-controlled trial. The Lancet Gastroenterology & Hepatology. 2025 Feb 1;10(2):138-53.

31. Vermeire S, Schreiber S, Petryka R, et al. Clinical remission in patients with moderate-to-severe Crohn's disease treated with filgotinib (the FITZROY study): results from a phase 2, double-blind, randomised, placebo-controlled trial. Lancet. 2017;389(10066):266-75. Epub 2016/12/19.

32. Vermeire S, D'Haens G, Baert F, et al. Efficacy and Safety of Subcutaneous Vedolizumab in Patients With Moderately to Severely Active Crohn's Disease: Results From the VISIBLE 2 Randomised Trial. J Crohns Colitis. 2022;16(1):27-38. Epub 2021/08/18.

33. Volkers A, Jansen J. SIMILAR trial—efficacy of infliximab-biosimilar compared to infliximab-biological in patients with inflammatory bowel disease in remission—a randomized, controlled, double blind, phase 4 noninferiority trial. United Eur Gastroenterol J. 2017;5:A307.

34. Watanabe M, Hibi T, Lomax KG, et al. Adalimumab for the induction and maintenance of clinical remission in Japanese patients with Crohn's disease. J Crohns Colitis. 2012;6(2):160-73. Epub 2012/02/14.

35. Watanabe K, Motoya S, Ogata H, et al. Effects of vedolizumab in Japanese patients with Crohn's disease: a prospective, multicenter, randomized, placebo-controlled Phase 3 trial with exploratory analyses. J Gastroenterol. 2020;55(3):291-306. Epub 2019/12/15.

36. Willoughby JM, Beckett J, Kumar PJ, Dawson AM. Controlled trial of azathioprine in Crohn's disease. Lancet. 1971;2(7731):944-7. Epub 1971/10/30.

37. Young D, Harris C, Rahmany S, Iria I, Gonçalves J, Addison J, Harvey J, Latter S, Cummings F. A randomised, crossover trial exploring the patient perspective and effectiveness of biosimilar adalimumab transition: IBD reference and biosimilar adalimumab cross over study (iBaSS). International Journal of Clinical Pharmacy. 2024 Oct;46(5):1091-101.
