# Supplementary material for: Immunomodulators and advanced therapies for maintenance of remission in Crohn’s disease: systematic review and network meta-analysis
Source: Ther Adv Gastroenterol. 2026 Jul 27;19:17562848261470683. doi: 10.1177/17562848261470683 (PMC13408072; doi:10.1177/17562848261470683)
Supplement: sj-pdf-1-tag-10.1177_17562848261470683 – Supplemental material for Immunomodulators and advanced therapies for maintenance of remission in Crohn’s disease: systematic review and network meta-analysis [file sj-pdf-1-tag-10.1177_17562848261470683.pdf]

Figure 1. Risk of bias summary for the included studies in the advanced treatments for the maintenance of remission in Crohn’s Disease NMA (n=37.

	Random sequence generation (selection bias)	Allocation concealment (selection bias)	Blinding of participants and personnel (performance bias)	Blinding of outcome assessment (detection bias)	Incomplete outcome data (attrition bias)	Selective reporting (reporting bias)	Other bias
Buhl 2022 (STOP-IT)	+	+	+	+	+	?	+
Colombel 2007 (CHARM) - Maintenance (Responders)	+	+	+	+	?	+	?
Colombel 2023 - LIBERTY CD	?	?	+	?	?	+	?
Feagan 2000	+	+	+	+	+	+	+
Feagan 2014	+	+	+	+	+	+	+
Feagan 2015c (IM-UNITI)	+	+	+	?	+	+	+
Ferrante 2022 - FORTIFY	+	+	+	+	?	?	+
Hanauer 2002 (ACCENT I) - responders (maint.)	+	+	+	+	+	?	?
Jorgensen 2017 (NORSWITCH)	+	+	+	+	+	+	+
Lemann 2005	?	+	+	+	+	+	+
Loftus 2023 (U-ENDURE)	+	+	+	?	+	+	+
Louis 2023 - SPARE	+	+	−	−	+	+	+
Mantzaris 2009	+	?	−	−	+	+	+
O'Donoghue 1978	?	?	?	?	+	+	+
Panes 2013	+	+	+	+	+	+	+
Panes 2017 Maintenance	+	+	+	+	+	+	+
Roder 2021	+	?	+	?	+	?	?
Rosenberg 1975	?	+	+	+	+	+	+
Rutgeerts 1999	+	?	+	+	+	?	+
Rutgeerts 2012 (EXTEND)	?	?	+	+	+	+	+
Sandborn 2005a (ENACT 2)	+	+	+	+	?	?	+
Sandborn 2007b (CLASSIC II)	?	+	+	+	+	+	+
Sandborn 2012 (CERTIFI) - Maintenance	+	+	+	+	+	+	+
Sandborn 2013 (GEMINI II) - Maintenance	+	+	+	+	+	+	+
Sandborn 2023 - BERGAMOT Maintenance	+	+	+	?	+	+	+
Sands 2004 (ACCENT II) Maintenance	+	+	+	?	+	−	+
Schreiber 2007 (PRECISE 2)	+	+	+	+	−	+	+
Summers 1979 (Part 2)	?	+	+	+	?	+	+
Van Assche 2012 (SWITCH)	+	+	−	−	?	?	+
Vermeire 2017 (FITZROY -maintenance)	+	+	+	+	+	?	+
Vermeire 2021 (VISIBLE 2)	+	+	+	?	+	+	+
Vermeire 2025 (DIVERSITY - maintenance)	+	?	+	+	−	+	?
Volkers2017 (SIMILAR)	?	?	+	?	?	?	?
Watanabe 2012 - Maintenance	?	?	+	?	+	+	+
Watanabe 2020 - Maintenance	+	+	+	?	+	+	+
Willoughby 1971	?	?	+	+	?	+	+
Young 2024 - iBaSS	+	+	−	−	+	?	?

Table 1. Risk of bias judgements and justifications for the included studies in the advanced treatments for the maintenance of remission in Crohn's Disease NMA. Judgements for Lemann 2005; Mantzaris 2009; O'Donoghue 1978; Panes 2013; Rosenberg 1975; Summers 1979; Willoughby 1971; are taken from the Chande 2015 Cochrane systematic review (DOI: 10.1002/14651858.CD000067.pub3). Judgements for Feagan 2000; Feagan 2014; are taken from the Patel 2014 Cochrane systematic review (DOI: 10.1002/14651858.CD006884.pub3).

Buhl 2022 (STOP-IT)

Bias	Authors' judgement	Support for judgement
Random sequence generation (selection bias)	Low risk	Randomization was performed centrally at Herlev University Hospital, Denmark, by non-blinded laboratory personnel who did not have contact with the patients or physicians, and used opaque, sealed envelopes.
Allocation concealment (selection bias)	Low risk	A non-blinded nurse, who was not involved in the treatment of the patients, received the allocation results and subsequently prepared and labeled infliximab or placebo medication accordingly
Blinding of participants and personnel (performance bias)	Low risk	Matching infliximab and placebo infusions, participants and personnel were blinded
Blinding of outcome assessment (detection bias)	Low risk	The treating physicians who performed the assessments were blinded.
Incomplete outcome data (attrition bias)	Low risk	Attrition was balanced and explained in both groups. Not likely to have influenced outcomes
Selective reporting (reporting bias)	Unclear risk	The trial has been registered and a protocol was published in 2014.<br>The primary and secondary outcomes were swapped, before blinding was broken, and this was justified as "using the originally proposed primary endpoint would not have incorporated all data collected, given that patients discontinuing treatment for reasons other than relapse would not have been included in the analysis, thus decreasing the power".<br>Authors stated that analyses of key secondary endpoints were performed only if the primary efficacy analysis yielded statistically significant results.
Other bias	Low risk	Baseline characteristics are balanced. No other concerns.

Colombel 2007 (CHARM) - Maintenance (Responders)

Bias	Authors' judgement	Support for judgement
Random sequence generation (selection bias)	Low risk	Author states that patients were "randomised centrally using an interactive voice response system".
Allocation concealment (selection bias)	Low risk	Author states "Patients, study coordinators, and study investigators were blinded to treatment assignment throughout the blinded portion of the study".
Blinding of participants and personnel (performance bias)	Low risk	Author states that "Patients, study coordinators, and study investigators were blinded to treatment assignment throughout the blinded portion of the study".
Blinding of outcome assessment (detection bias)	Low risk	Author states that "Patients, study coordinators, and study investigators were blinded to treatment assignment throughout the blinded portion of the study".
Incomplete outcome data (attrition bias)	Unclear risk	Very high attrition in the placebo responder group compared to the adalimumab responders groups. In placebo, 32/170 responders had completed the 56 week while 77/172 and 82/157 were completion for the adalimumab group eow and weekly, respectively. Whilst adverse events was mentioned as the most common, full data for discontunuations is not given.
Selective reporting (reporting bias)	Low risk	The one outcome in the trial registration (clinical remission) has been reported
Other bias	Unclear risk	Baseline characteristics not reported for the three groups - placebo, ADA 40mg eow and ADA 40mg weekly. Authors were contacted for clarification.

Colombel 2023 - LIBERTY CD

Bias	Authors' judgement	Support for judgement
Random sequence generation (selection bias)	Unclear risk	No details (abstract only publication)
Allocation concealment (selection bias)	Unclear risk	No details (abstract only publication)
Blinding of participants and personnel (performance bias)	Low risk	Placebo-controlled trials
Blinding of outcome assessment (detection bias)	Unclear risk	No details (abstract only publication)
Incomplete outcome data (attrition bias)	Unclear risk	35 patients did not complete the CT-P13 protocol vs 25 for the placebo. No further details.

Selective reporting (reporting bias)	Low risk	Registered prospectively (NCT03945019). Outcomes reported are appropriate and match the trial registration.
Other bias	Unclear risk	Sex imbalance at randomisation

Feagan 2000

Bias	Authors' judgement	Support for judgement
Random sequence generation (selection bias)	Low risk	Computer generated randomization code
Allocation concealment (selection bias)	Low risk	Allocation concealment was adequate. Medication was administered in coded identical pre-filled vials which were administered serially to participants
Blinding of participants and personnel (performance bias)	Low risk	Active and placebo medications were identical in appearance and were prepared in prefilled vials Clinical data were independently reviewed by two investigators who were unaware of the patients' treatment assignments
Blinding of outcome assessment (detection bias)	Low risk	Active and placebo medications were identical in appearance and were prepared in prefilled vials Clinical data were independently reviewed by two investigators who were unaware of the patients' treatment assignments
Incomplete outcome data (attrition bias)	Low risk	There was only one drop-out. A patient withdrew from the methotrexate group due to an adverse event (nausea)
Selective reporting (reporting bias)	Low risk	The published report includes all expected outcomes
Other bias	Low risk	The study appears to be free of other sources of bias

Feagan 2014

Bias	Authors' judgement	Support for judgement
Random sequence generation (selection bias)	Low risk	Randomly assigned by computer
Allocation concealment (selection bias)	Low risk	Centralized randomization
Blinding of participants and personnel (performance bias)	Low risk	Double-blind with identically appearing placebo
Blinding of outcome assessment (detection bias)	Low risk	Double-blind with identically appearing placebo

Incomplete outcome data (attrition bias)	Low risk	Six patients withdrew from the study for reasons not related to treatment failure;two patients were assigned to methotrexate (both due to adverse events) and four patients were assigned to placebo (two withdrew consent, one due to adverse event, one lost to follow-up)
Selective reporting (reporting bias)	Low risk	All outcomes were reported
Other bias	Low risk	No other issues

Feagan 2015c (IM-UNITI)

**Bias**	**Authors' judgement**	**Support for judgement**
Random sequence generation (selection bias)	Low risk	Randomisation was described as 1:1:1 and performed "centrally with the use of permuted blocks".
Allocation concealment (selection bias)	Low risk	Randomisation was described as 1:1:1 and performed "centrally with the use of permuted blocks".
Blinding of participants and personnel (performance bias)	Low risk	Double-blind placebo controlled trial<br>From the protocol: "To maintain the study blind, the study agent container will have a multilingual<br>label containing the study name, medication number, and reference number, but the label will not identify the study agent in the container. A tear-off label is designed to be separated from the study agent container and attached to the subject's source documents. The medication number will be entered in the CRF when the drug is dispensed. The study agents will be identical in appearance and packaging"
Blinding of outcome assessment (detection bias)	Unclear risk	Not mentioned. Authors were contacted for clarification.
Incomplete outcome data (attrition bias)	Low risk	Authors mention attrition was low but the study flow is unclear and not presented in the supplementary figures mentioned in the text
Selective reporting (reporting bias)	Low risk	Reported outcomes match those described in the trial registration.
Other bias	Low risk	Authors reported balanced baseline characteristics in each group. No other sources of bias.

Ferrante 2022 - FORTIFY

**Bias**	**Authors' judgement**	**Support for judgement**

Random sequence generation (selection bias)	Low risk	Randomised and assigned via interactive response technology.<br>Patients with a low SES-CD at the induction study baseline, patients who received 24 weeks of induction dosing, and patients from a non-compliant site (ie, without investigator oversight) were also randomly assigned and were included in the safety population, but not in the efficacy population.
Allocation concealment (selection bias)	Low risk	To maintain blinding, risankizumab and placebo kits were identical in appearance. Study<br>investigators enrolled articipants. Interactive response technology determined assignment of<br>participants to a treatment group.
Blinding of participants and personnel (performance bias)	Low risk	Kits identical in appearance. Patients, investigators, and study personnel involved in the trial conduct or analyses were masked to treatment assignments until study completion.
Blinding of outcome assessment (detection bias)	Low risk	Kits identical in appearance. Patients, investigators, and study personnel involved in the trial conduct or analyses were masked to treatment assignments until study completion.
Incomplete outcome data (attrition bias)	Unclear risk	From the randomised patients, 22 assigned to risa 180mg did not receive the intervention, 38 in the 360mg group and 20 in the placebo group. 144, 124 and 144 completed the trial and the reasons for leaving the study are equally distributed.
Selective reporting (reporting bias)	Unclear risk	The study has been prospectively registered, however the primary outcomes are not entirely the same between paper and trial registration. The outcomes reported are appropriate.
Other bias	Low risk	No baseline imbalances. No other sources of bias.

Hanauer 2002 (ACCENT I) - responders (maintenance)

Bias	Authors' judgement	Support for judgement
Random sequence generation (selection bias)	Low risk	Authors mention random assignment via adaptive randomisation voice response system
Allocation concealment (selection bias)	Low risk	"Allocation of patients to a treatment group was done with an adaptive stratified design" and<br>"allocate patients centrally to treatment based on the current balance of treatment groups within<br>each stratum".
Blinding of participants and personnel (performance bias)	Low risk	"Neither the patients nor study investigators were aware of the treatment assignment" and<br>"pharmacist prepared the infusion (infliximab [Remicade] or an identically appearing placebo".

Blinding of outcome assessment (detection bias)	Low risk	"Neither the patients nor study investigators were aware of the treatment assignment" and "pharmacist prepared the infusion (infliximab [Remicade] or an identically appearing placebo".
Incomplete outcome data (attrition bias)	Low risk	Safety data is presented for the entire cohort throughout the end of the study. No concers that attrition might have affected the results.
Selective reporting (reporting bias)	Unclear risk	Outcomes reported as per the methods section. The trial registration provides little information so we can't be sure if the pre-specified plan was followed.
Other bias	Unclear risk	Baseline characteristics were not provided per treatment group for the week-2 resonders.

Jorgensen 2017 (NORSWITCH)

**Bias**	**Authors' judgement**	**Support for judgement**
Random sequence generation (selection bias)	Low risk	This was computer-generated randomised as stated by the authors.
Allocation concealment (selection bias)	Low risk	Author stated "computer-generated randomised allocation sequence was imported into the electronic case report form (eCRF) system (Viedoc; version 3.20) and made available

		exclusively to the study nurse authorised by the local principal investigator to prepare infusions". The author has confirmed the nurse was unrelated to the study.
Blinding of participants and personnel (performance bias)	Low risk	Author stated "site person authorised for infusion preparation logged into the eCRF system to reveal the allocation, prepared allocated treatment in identical infusion bags, and applied labels with patient number and dose" and specified that the "following personnel were not masked to treatment allocation: the statistician preparing the randomised allocation sequence; the data manager importing the allocation sequence into the eCRF system and providing access to the allocation sequence; and site personnel authorised to prepare study treatment". Author further stated "All individuals providing patient care were masked to treatment allocation, including investigators, nurses giving infusions, and personnel assessing outcomes. Monitors and patients were also masked to treatment allocation".
Blinding of outcome assessment (detection bias)	Low risk	Author stated "All individuals providing patient care were masked to treatment allocation, including investigators, nurses giving infusions, and personnel assessing outcomes. Monitors and patients were also masked to treatment allocation"
Incomplete outcome data (attrition bias)	Low risk	Attrition in both groups balanced and all numbers were accounted for with clear reasons provided.
Selective reporting (reporting bias)	Low risk	The author reported according to their methd section on Harvey Bradshaw Index (HBI) for Crohn's Disease
Other bias	Low risk	Baseline characteristics for each group reported and balanced. No other apparent sources of bias.

Lemann 2005

Bias	Authors' judgement	Support for judgement
Random sequence generation (selection bias)	Unclear risk	Not adequately described
Allocation concealment (selection bias)	Low risk	Randomization was performed centrally by using permutation tables of size 2 or 4 after stratification by centre according to the number of patients anticipated to be enrolled at each centre

Blinding of participants and personnel (performance bias)	Low risk	Double-blinded, matched placebo for dose regime, appearance and taste to<br>AZA (25 mg tablets). PI blinding maintained at each site by a co-investigator, who did not have<br>physical contact with the patients, reviewing biochemical data and reporting back to PI on a case form. Any dosing changes were completed on a pre-specified schedule based on hematologic results
Blinding of outcome assessment (detection bias)	Low risk	Double-blinded, matched placebo for dose regime, appearance and taste to<br>AZA (25 mg tablets). PI blinding maintained at each site by a co-investigator, who did not have<br>physical contact with the patients, reviewing biochemical data and reporting back to PI on a case form. Any dosing changes were completed on a pre-specified schedule based on hematologic results
Incomplete outcome data (attrition bias)	Low risk	6/83 patients withdrew from the study with reasons given (2 from azathioprine<br>group, 3 from placebo group)<br>Data was analyzed by intention-to-treat
Selective reporting (reporting bias)	Low risk	Relaspe rates and adverse events were reported
Other bias	Low risk	The study appears to be free of other sources of bias

Loftus 2023 (U-ENDURE)

Bias	Authors' judgement	Support for judgement
Random sequence generation (selection bias)	Low risk	"Patients were randomly assigned through interactive response technology"
Allocation concealment (selection bias)	Low risk	"Patients were randomly assigned through interactive response technology"
Blinding of participants and personnel (performance bias)	Low risk	Double-blind placebo controlled study
Blinding of outcome assessment (detection bias)	Unclear risk	No information apart from endoscopy. "Endoscopic scores were centrally read by qualified gastroenterologists that were blinded to the site, subject number, timepoint, date of the endoscopy, and study treatment."
Incomplete outcome data (attrition bias)	Low risk	Outcomes reported appropriately and as per the trial registration.
Selective reporting (reporting bias)	Low risk	Outcomes reported appropriately and as per the trial registration.
Other bias	Low risk	Baseline characteristics similar between groups.

Louis 2023 - SPARE

Bias	Authors' judgement	Support for judgement
Random sequence generation (selection bias)	Low risk	"The patient number and group of each stratum were assigned by a central online randomisation website (Cleanweb). An independent statistician from the Biostatistics Unit of Saint-Louis Hospital (Paris, France) provided computergenerated assignment blocked randomisation lists with the use of a block size of six, unknown to the investigators."
Allocation concealment (selection bias)	Low risk	"Information about allocation was given on the eCRF and confirmed by email."
Blinding of participants and personnel (performance bias)	High risk	"Participants, those assessing outcomes, and those analysing the data were not masked to group assignment."
Blinding of outcome assessment (detection bias)	High risk	"Participants, those assessing outcomes, and those analysing the data were not masked to group assignment."
Incomplete outcome data (attrition bias)	Low risk	Equal attrition between groups, the reasons reported and equal per groups
Selective reporting (reporting bias)	Low risk	The outcome results have been presented per the trial registration
Other bias	Low risk	No concerns.

Mantzaris 2009

Bias	Authors' judgement	Support for judgement
Random sequence generation (selection bias)	Low risk	Computer-based random number generator
Allocation concealment (selection bias)	Unclear risk	Not adequately described
Blinding of participants and personnel (performance bias)	High risk	Blinding was limited to the endoscopist and pathologist Patients were aware of the intervention they were receiving, but were unaware of the second study arm
Blinding of outcome assessment (detection bias)	High risk	Blinding was limited to the endoscopist and pathologist Patients were aware of the intervention they were receiving, but were unaware of the second study arm
Incomplete outcome data (attrition bias)	Low risk	Missing data was accounted for with explanation 8 patients (6 due to relapse and 2 from adverse events) from the AZA arm and 14 patients from the budesonide arm (all due to relapse) failed. Analysis was performed on an intention-to-treat basis

Selective reporting (reporting bias)	Low risk	Primary outcome and some secondary outcomes described in the methods section were reported as results
Other bias	Low risk	The study appears to be free of other sources of bias

O'Donoghue 1978

Bias	Authors' judgement	Support for judgement
Random sequence generation (selection bias)	Unclear risk	Method of randomization not described in the published study
Allocation concealment (selection bias)	Unclear risk	Not described in the published study
Blinding of participants and personnel (performance bias)	Unclear risk	Not described in the published study
Blinding of outcome assessment (detection bias)	Unclear risk	Not described in the published study
Incomplete outcome data (attrition bias)	Low risk	10/51 patients withdrew because of relapse which was classified as a end point and a reason for withdrawal 5/51 patients withdrew for other reasons (3 taking azathioprine and 2 taking<br>placebo)<br>It was not clear how these patients were classified with regards to reason for<br>withdrawal
Selective reporting (reporting bias)	Low risk	Primary outcome described in the methods section was reported
Other bias	Low risk	The study appears to be free of other sources of bias

Panes 2013

Bias	Authors' judgement	Support for judgement
Random sequence generation (selection bias)	Low risk	Randomization was performed centrally with the use of an adaptive randomization procedure stratified according to age (< 40 years) and use of systemic corticosteroids at the time of inclusion (yes or no)
Allocation concealment (selection bias)	Low risk	Centralized randomization
Blinding of participants and personnel (performance bias)	Low risk	Double-blind<br>Patients, study site personnel, and study investigators were unaware of the treatment assignment

		The local investigator was not blinded;performed dose adjustment if bone marrow suppression was observed in the blood tests
Blinding of outcome assessment (detection bias)	Low risk	Double-blind<br>Patients, study site personnel, and study investigators were unaware of the treatment assignment<br>The local investigator was not blinded;performed dose adjustment if bone marrow suppression was observed in the blood tests
Incomplete outcome data (attrition bias)	Low risk	Any missing patient data is described and justified throughout the screening and study period<br>Description of analysis as intention-to-treat, with missing data accounted for
Selective reporting (reporting bias)	Low risk	Primary and secondary endpoints described in the methods section were reported in the results section<br>Post hoc analyses evaluating requirement of corticosteroids in patients under 40 years and the presence or perianal disease as predictors of disabling disease
Other bias	Low risk	The study appears to be free of other sources of bias

Panes 2017 Maintenance

**Bias**	**Authors' judgement**	**Support for judgement**
Random sequence generation (selection bias)	Low risk	Assignment of subject identification number and study drug were managed by a tele-randomisation system, by which the subject was enrolled online or via a telephone call.<br>In the maintenance study, patients were stratified and randomised according to their treatment assignments in the induction study, and their clinical remission status at week 8 of the induction study.
Allocation concealment (selection bias)	Low risk	Assignment of subject identification number and study drug were managed by a tele-randomisation system, by which the subject was enrolled online or via a telephone call.<br>Treatment randomisation information remained confidential and was not released to the investigator or study staff until the conclusion of the studies.
Blinding of participants and personnel (performance bias)	Low risk	Study treatment was blinded to patients, investigators and the sponsor.
Blinding of outcome assessment (detection bias)	Low risk	Study treatment was blinded to patients, investigators and the sponsor.
Incomplete outcome data (attrition bias)	Low risk	Balanced and explained attrition between groups.
Selective reporting (reporting bias)	Low risk	All outcomes reported per the trial registration (NCT01393899).

Other bias	Low risk	Baseline characteristics balanced. No other concerns.

Roder 2021

Bias	Authors' judgement	Support for judgement
Random sequence generation (selection bias)	Low risk	"computer-controlled randomization"
Allocation concealment (selection bias)	Unclear risk	No information
Blinding of participants and personnel (performance bias)	Low risk	Double-blind study
Blinding of outcome assessment (detection bias)	Unclear risk	No information
Incomplete outcome data (attrition bias)	Low risk	Reasons for discontinuation reported (5 in the originator and 11 in the biosimilar group, due to adverse events and lack of efficacy).
Selective reporting (reporting bias)	Unclear risk	No trial registration found
Other bias	Unclear risk	The baseline characteristics for the CD patients per group are unclear

Rosenberg 1975

Bias	Authors' judgement	Support for judgement
Random sequence generation (selection bias)	Unclear risk	Not described
Allocation concealment (selection bias)	Low risk	Patients were allocated randomly to each group by the pharmacist such that of every 10 patients entering the study half were in each group
Blinding of participants and personnel (performance bias)	Low risk	Double blinded, with an identically-matched placebo Physicians were blinded to the drug administration; two physicians working independently of one another assumed responsibility for the total care of each patient: one physician managed the clinical course, recorded data, and adjusted the dose of prednisone, while the other monitored blood counts for evidence of toxicity
Blinding of outcome assessment (detection bias)	Low risk	Double blinded, with an identically-matched placebo Physicians were blinded to the drug administration; two physicians working independently of one another assumed responsibility for the total care of each patient: one physician managed the clinical course, recorded data, and adjusted the dose of prednisone, while the other monitored blood counts for evidence of toxicity

Incomplete outcome data (attrition bias)	Low risk	One patient failed to complete the study
Selective reporting (reporting bias)	Low risk	Primary outcome described in the methods section was reported
Other bias	Low risk	The study appears to be free of other sources of bias

Rutgeerts 1999

**Bias**	**Authors' judgement**	**Support for judgement**
Random sequence generation (selection bias)	Low risk	"Randomly assigned in a 1:1 ratio to retreatment with 10 mg/kg infliximab or placebo at week 12 by an independent organization".
Allocation concealment (selection bias)	Unclear risk	Not mentioned.
Blinding of participants and personnel (performance bias)	Low risk	"Investigators, all other study personnel, and patients were kept blinded at the time patients were determined to be eligible for the retreatment extension".
Blinding of outcome assessment (detection bias)	Low risk	"Investigators, all other study personnel, and patients were kept blinded at the time patients were determined to be eligible for the retreatment extension".
Incomplete outcome data (attrition bias)	Low risk	Reasons for attrition were explained. There was a small imbalance between groups but did not affect outcomes
Selective reporting (reporting bias)	Unclear risk	No prespecified plan or trial registration
Other bias	Low risk	Baseline characteristics were reported for each treatment group and balanced. No other apparent source of bias.

Rutgeerts 2012 (EXTEND)

**Bias**	**Authors' judgement**	**Support for judgement**
Random sequence generation (selection bias)	Unclear risk	Not mentioned
Allocation concealment (selection bias)	Unclear risk	Not mentioned
Blinding of participants and personnel (performance bias)	Low risk	Described as double-blinded
Blinding of outcome assessment (detection bias)	Low risk	Author states "ileocolonoscopy procedures were recorded and subsequently evaluated by an independent, blinded central reviewer".

Incomplete outcome data (attrition bias)	Low risk	Attrition was accounted for and balanced in both groups, with adequate reasons provided.
Selective reporting (reporting bias)	Low risk	Authors reported relevant outcomes as planned per the trial registration
Other bias	Low risk	Baseline characteristics reported and balanced between both groups. No other apparent sources of bias.

Sandborn 2005a (ENACT 2)

Bias	Authors' judgement	Support for judgement
Random sequence generation (selection bias)	Low risk	"Both trials (ENACT-1 AND -2) were centrally randomised".
Allocation concealment (selection bias)	Low risk	"Centrally allocated"
Blinding of participants and personnel (performance bias)	Low risk	Patients and investigators were unaware of treatment assignments, stated as double blinded.
Blinding of outcome assessment (detection bias)	Low risk	Patients and investigators were unaware of treatment assignments, stated as double blinded.
Incomplete outcome data (attrition bias)	Unclear risk	Atrrition is balanced in the groups but no reasons for attrition have been given.
Selective reporting (reporting bias)	Unclear risk	No protocol and registrtion has no outcomes. However, clinically relevant outcomes have been reported.
Other bias	Low risk	No other sources apparent. No baseline characteristic imbalance.

Sandborn 2007b (CLASSIC II)

Bias	Authors' judgement	Support for judgement
Random sequence generation (selection bias)	Unclear risk	No randomisation method explained.
Allocation concealment (selection bias)	Low risk	Authors state assignment "was done centrally"
Blinding of participants and personnel (performance bias)	Low risk	Authors state "the patients, study coordinators, and study investigators were all blinded to treatment assignments"
Blinding of outcome assessment (detection bias)	Low risk	Although authors state "the patients, study coordinators, and study investigators were all blinded to treatment assignments",

Incomplete outcome data (attrition bias)	Low risk	Attrition was balanced and accounted for in all three groups with adequate reasons provided.
Selective reporting (reporting bias)	Low risk	Outcomes have been reported per the trial registration.
Other bias	Low risk	Baseline characteristics reported and balanced in all three groups. No other sources of apparent bias.

Sandborn 2012 (CERTIFI) - Maintenance

**Bias**	**Authors' judgement**	**Support for judgement**
Random sequence generation (selection bias)	Low risk	Author states "Adaptive randomization, performed centrally".
Allocation concealment (selection bias)	Low risk	Author states "Adaptive randomization, performed centrally".
Blinding of participants and personnel (performance bias)	Low risk	Sponsor response after our enquire: To protect the integrity of the maintenance phase of the study,treatment assignment

		blinding was to be maintained for investigative sites, site monitors, and subjects participating in the study until the Week 36 analyses were completed. Three database locks were planned for the study: the first when all subjects in the induction phase either completed the Week 6 visit or terminated study participation before Week 6; the second when all subjects in the maintenance phase either completed the Week 22 visit or terminated study participation before Week 22; and the third when all subjects in the maintenance phase either completed the Week 36 visit or terminated study participation before Week 36. The sponsor was to remain blinded to treatment assignment until the first database lock. At the first database lock, data were to be unblinded for subjects in the induction phase of the study;data for the maintenance phase of the study were to remain blinded. At the second database lock, data from the maintenance phase of the study were to be unblinded for analysis. Identification of sponsor personnel who would have access to the unblinded subject-level data was to be documented before unblinding at the Week 6 and Week 22 database locks. Data that might potentially unblind the treatment assignment (ie, study agent serum concentrations, antibodies to study agent, treatment allocation, and study agent preparation/accountability data) was to be handled with special care so that, before unblinding, such data would be available only to data management staff for purposes of data cleaning, bioanalysis staff for serum concentration assays, and, if applicable, QA representatives for the purposes of conducting independent drug audits.
Blinding of outcome assessment (detection bias)	Low risk	Sponsor response after our enquire: To protect the integrity of the maintenance phase of the study,treatment assignment blinding was to be maintained for investigative sites, site monitors, and subjects participating in the study until the Week 36 analyses were completed.Three database locks were planned for the study: the first when all subjects in the induction phase either completed the Week 6 visit or terminated study participation before Week 6; the second when all subjects in the maintenance phase either completed the Week 22 visit or terminated study participation before Week 22; and the third when all subjects in the maintenance phase either completed the Week 36 visit or terminated study participation before Week 36. The sponsor was to remain blinded to treatment assignment until the first database lock. At the first database lock, data were to be unblinded for subjects in the induction phase of the study; data for the maintenance phase of the study were to remain blinded. At the second database lock, data from the maintenance phase of the study were to be unblinded for analysis.

		Identification of sponsor personnel who would have access to the unblinded subject-level data was to be documented before unblinding at the Week 6 and Week 22 database locks.<br>Data that might potentially unblind the treatment assignment (ie, study agent serum concentrations, antibodies to study agent, treatment allocation, and study agent preparation/accountability data) was to be handled with special care so that, before unblinding, such data would be available only to data management staff for purposes of data cleaning, bioanalysis staff for serum concentration assays, and, if applicable, QA representatives for the purposes of conducting independent drug audits.
Incomplete outcome data (attrition bias)	Low risk	According to the flowchart of Supplementary Figure 2 (S2), attrition was balanced in all groups with adequate reasons provided for loss in numbers shown in Tables S3A and S3B.
Selective reporting (reporting bias)	Low risk	Authors reported outcomes according to trial registration - clinical response and clinical remission (CDAI scores) at relevant intervals.
Other bias	Low risk	Baseline characteristics reported and balanced for patients in all groups. No other apparent sources of bias.

Sandborn 2013 (GEMINI II) - Maintenance

**Bias**	**Authors' judgement**	**Support for judgement**
Random sequence generation (selection bias)	Low risk	Author states "Randomization was computer-generated and was performed at a central location."
Allocation concealment (selection bias)	Low risk	Authors state that randomisation "was performed at a central location".
Blinding of participants and personnel (performance bias)	Low risk	Double-blind study
Blinding of outcome assessment (detection bias)	Low risk	Author states "sponsor [Millenium Pharmaceuticals] collected and analyzed the data".
Incomplete outcome data (attrition bias)	Low risk	According to the flowchart of Supplementary Figure 1 (S1), attrition was balanced in all groups with adequate reasons provided for loss in numbers.
Selective reporting (reporting bias)	Low risk	Relevant outcomes reported per trial registration
Other bias	Low risk	Baseline characteristics reported and balanced for patients in all groups. No other apparent sources of bias.

Sandborn 2023 - BERGAMOT Maintenance

Bias	Authors' judgement	Support for judgement
Random sequence generation (selection bias)	Low risk	"An independent, interactive voice web-based response system provided by Parexel Newton, MA) was used to generate the randomisation list and randomly assign patients to a treatment group"
Allocation concealment (selection bias)	Low risk	"Blinded kit identification numbers were used to dispense study treatment"
Blinding of participants and personnel (performance bias)	Low risk	Matched placebo. No further detail
Blinding of outcome assessment (detection bias)	Unclear risk	No detail
Incomplete outcome data (attrition bias)	Low risk	40% of randomised patients left the placebo group due to lack of efficacy compared to 30% of the etrolizumab group. However, as all other attrition reasons were balanced we did not think that 10% difference could have sufficiently influenced outcome results
Selective reporting (reporting bias)	Low risk	Prospectively registered at NCT02394028. The outcomes reported are appropriate and match the trial registration.
Other bias	Low risk	No concerns.

Sands 2004 (ACCENT II) Maintenance

Bias	Authors' judgement	Support for judgement
Random sequence generation (selection bias)	Low risk	A computer-generated adaptive randomization scheme was used, which included the study site, the number of draining fistulas at baseline (one vs. more than one), and the presence or absence of active bowel disease at baseline (active bowel disease was considered to be present if the Crohn’s Disease Activity Index was at least 150) as stratification factors.
Allocation concealment (selection bias)	Low risk	A pharmacist prepared each infusion of infliximab or an identical appearing placebo. Neither the patients nor the study investigators were aware of the treatment assignment. Crossovers were masked so that patients and physicians remained unaware of the treatment assignment.
Blinding of participants and personnel (performance bias)	Low risk	A pharmacist prepared each infusion of infliximab or an identical appearing placebo. Neither the patients nor the study investigators were aware of the treatment assignment. Crossovers were masked so that patients and physicians remained unaware of the treatment assignment.

Blinding of outcome assessment (detection bias)	Unclear risk	It is not reported whether the clinicians conducting the assessments and examinations at study visits were blinded to treatment assignments
Incomplete outcome data (attrition bias)	Low risk	Low and balanced attrition with no influence on outcomes
Selective reporting (reporting bias)	High risk	The reported outcomes have shifted from those published in the trial registration (NCT00207766)
Other bias	Low risk	Baseline characteristics are balanced across the study arms. No other concerns

Schreiber 2007 (PRECISE 2)

Bias	Authors' judgement	Support for judgement
Random sequence generation (selection bias)	Low risk	Author states "randomization code...was generated by an independent contractor"
Allocation concealment (selection bias)	Low risk	Authors state "Randomization was centralized by means of an interactive voice-recognition system".
Blinding of participants and personnel (performance bias)	Low risk	Author states "Patients and investigators were un-aware of the group assignment"
Blinding of outcome assessment (detection bias)	Low risk	Author states "Patients and investigators were un-aware of the group assignment"
Incomplete outcome data (attrition bias)	High risk	Considerable more participants withdrew from the placebo group compared to the certolizumab<br>group, the imbalance being cauased mainly due to "lack of improvement or worsening of disease". It's likely this has affected our outcome results.
Selective reporting (reporting bias)	Low risk	Outcomes reported per the trial registration
Other bias	Low risk	Baseline characteristics reported for and balanced in both groups. No other apparent sources of bias.

Summers 1979 (Part 2)

Bias	Authors' judgement	Support for judgement
Random sequence generation (selection bias)	Unclear risk	Not described
Allocation concealment (selection bias)	Low risk	Centralized randomization
Blinding of participants and personnel (performance bias)	Low risk	Double-blinded, identically matched "all prepared in uncoated tablets of identical<br>external and internal appearance"

Blinding of outcome assessment (detection bias)	Low risk	Double-blinded, identically matched "all prepared in uncoated tablets of identical<br>external and internal appearance"
Incomplete outcome data (attrition bias)	Unclear risk	Attrition appears to be low (possible secondary to the fact that reasons for exiting the study were included in the "Outcome Ranking Scheme")<br>A subset of patients were randomized and then excluded after study completion (justification was wrong diagnosis or inappropriate entry)
Selective reporting (reporting bias)	Low risk	All outcomes described for each part and phase were reported
Other bias	Low risk	The study appears to be free of other sources of bias

Van Assche 2012 (SWITCH)

Bias	Authors' judgement	Support for judgement
Random sequence generation (selection bias)	Low risk	Authors state "Random allocation was based on a centrally stored randomly generated list that was not accessible to the investigators"
Allocation concealment (selection bias)	Low risk	Authors state "Random allocation was based on a centrally stored randomly generated list that was not accessible to the investigators"
Blinding of participants and personnel (performance bias)	High risk	Open-label study
Blinding of outcome assessment (detection bias)	High risk	Open-label study
Incomplete outcome data (attrition bias)	Unclear risk	Authors reported higher attrition with adalimumab group (10/36) versus infliximab (1/37). Respective reasons are provided. There is a chance this might have affected outcomes.
Selective reporting (reporting bias)	Unclear risk	Trial registered NCT01338740. The authors reported the proportion of patients with specific CDAI score changes, however the reporting of remission rates is unclear.
Other bias	Low risk	Baseline characteristics were reported for and balanced in both groups. No other apparent sources of bias.

Vermeire 2017 (FITZROY -maintenance)

Bias	Authors' judgement	Support for judgement
Random sequence generation (selection bias)	Low risk	A prespecified randomisation scheme prepared by an independent statistician was used to randomly allocate patients to treatment groups.

Allocation concealment (selection bias)	Low risk	For each patient at each visit, the clinic contacted the interactive web-based response system to obtain a treatment number corresponding to the appropriate study drug.
Blinding of participants and personnel (performance bias)	Low risk	Patients, investigators, study coordinators, the sponsor, and the entire study team were masked to treatment assignment. Filgotinib and placebo were presented as orally administered brown fi lm-coated tablets that were identical in appearance and contained the same excipients
Blinding of outcome assessment (detection bias)	Low risk	For the efficacy reads, assessment was done by two independent central readers in a masked fashion. In the case of discrepant results, a third, independent read by an adjudicator was done and was used as the final determination of efficacy read.
Incomplete outcome data (attrition bias)	Low risk	Authors provided us with the data and it's balanced and the reasons explained
Selective reporting (reporting bias)	Unclear risk	The trial registation (NCT02048618) does not include the outcomes for the maintenance part of the trial. Relapse data are not clearly presented for the re-randomised group.
Other bias	Low risk	Authors provided us with the maintenance baseline data which are balanced and we have no other concerns

Vermeire 2021 (VISIBLE 2)

**Bias**	**Authors' judgement**	**Support for judgement**
Random sequence generation (selection bias)	Low risk	The author confirmed that "The randomization schedule was generated randomly by a vendor who provided us with the interactive response technology for randomization"
Allocation concealment (selection bias)	Low risk	The author confirmed that "The personnel from the vendor who generated and had access to the live randomization schedule were not involved in the study conduct or data analysis. "
Blinding of participants and personnel (performance bias)	Low risk	This was a "double-blind, placebo-controlled" study
Blinding of outcome assessment (detection bias)	Unclear risk	Not mentioned.
Incomplete outcome data (attrition bias)	Low risk	Attrition was accounted for and balanced in both groups with adequate reasons provided for loss in numbers.
Selective reporting (reporting bias)	Low risk	According to trial registration and method section, authors reported the necessary endpoints -proportion of patients with clinical remission and response.
Other bias	Low risk	Baseline characteristics were reported for and balanced in all groups. No other apparent sources of bias.

Vermeire 2021 (DIVERSITY - maintenance)

Bias	Authors' judgement	Support for judgement
Random sequence generation (selection bias)	Low risk	Randomisation was done by the investigator through an interactive web response system
Allocation concealment (selection bias)	Unclear risk	No mention
Blinding of participants and personnel (performance bias)	Low risk	Everyone directly involved in the study conduct (including investigators, study personnel, and patients) was fully blinded to treatment allocation until the last patient completed the follow-up visit 30 days after completing 58 weeks of treatment. The appearance, packaging, and handling of active treatment (filgotinib 200 mg and filgotinib 100 mg) and placebo were identical to maintain blinding.
Blinding of outcome assessment (detection bias)	Low risk	All outcome assessors were blinded. Endoscopic central reading. Adverse events and clinical laboratory results were coded using the Medical Dictionary for Regulatory Activities version 25.0, and their severity was graded using the modified Common Terminology Criteria for Adverse Events version 4.03. An external, multidisciplinary data monitoring committee performed interim reviews of the safety data throughout the trial. All potential major adverse cardiovascular events (MACEs) and venous thromboembolic (VTE) events were reviewed and adjudicated periodically, and gastrointestinal perforation events were reviewed and adjudicated post hoc, in a blinded manner by an independent expert committee.
Incomplete outcome data (attrition bias)	High risk	High attrition rates for all groups (57.1% for placebo, 56.1% for filgotinib 100mg, 45.7% for filgotinib 200mg)
Selective reporting (reporting bias)	Low risk	Outcomes appropriately reported per trial registration. NCT02914561
Other bias	Unclear risk	There are group differences in the number of biologics received before the trial

Volkers2017 (SIMILAR)

Bias	Authors' judgement	Support for judgement
Random sequence generation (selection bias)	Unclear risk	Authors do not describe how they achieved randomisation.

Allocation concealment (selection bias)	Unclear risk	Authors do not describe how allocation was concealed.
Blinding of participants and personnel (performance bias)	Low risk	Described as a double-blind trial
Blinding of outcome assessment (detection bias)	Unclear risk	Not described.
Incomplete outcome data (attrition bias)	Unclear risk	Authors provide the overall numbers of patients withdrawing in the abstract but do not specify how many had CD and from the limited information provided, it is not possible to work out how many patients from each study arm withdrew<br>Authors only give information on primary outcome in the abstract but do not specify if it applies to CD or UC patients
Selective reporting (reporting bias)	Unclear risk	Preliminary results and not all outcome results clearly reported.<br>Trial registration NCT02452151.
Other bias	Unclear risk	No baseline information.

Watanabe 2012 - Maintenance

Bias	Authors' judgement	Support for judgement
Random sequence generation (selection bias)	Unclear risk	Not described
Allocation concealment (selection bias)	Unclear risk	Not described
Blinding of participants and personnel (performance bias)	Low risk	Double-blinded study
Blinding of outcome assessment (detection bias)	Unclear risk	Not described
Incomplete outcome data (attrition bias)	Low risk	Attrition was accounted for and balanced in both groups with adequate reasons provided for loss in numbers.
Selective reporting (reporting bias)	Low risk	According to trial registration and method section authors reported the relevant endpoints - CDAI scores at specific timepoints.
Other bias	Low risk	Baseline characteristics were reported for and balanced in all groups. No other apparent sources of bias.

Watanabe 2020 - Maintenance

Bias	Authors' judgement	Support for judgement

Random sequence generation (selection bias)	Low risk	Author states "Randomization schedules were generated by personnel designated by the sponsor"
Allocation concealment (selection bias)	Low risk	Author states "allocations were not disclosed until opening of the study drug allocation table,<br>except to unblinded pharmacists at each site".
Blinding of participants and personnel (performance bias)	Low risk	Double-blinded study
Blinding of outcome assessment (detection bias)	Unclear risk	Author states "allocations were not disclosed until opening of the study drug allocation table,<br>except to unblinded pharmacists at each site"
Incomplete outcome data (attrition bias)	Low risk	Attrition was accounted for and balanced in all groups with adequate reasons provided for loss in numbers.
Selective reporting (reporting bias)	Low risk	According to trial registration and method section authors reported the relevant endpoints -<br>proportion of patients with specific CDAI-100 response and clinical remission.
Other bias	Low risk	Baseline characteristics were reported for and balanced in all groups. No other apparent sources of bias.

Willoughby 1971

Bias	Authors' judgement	Support for judgement
Random sequence generation (selection bias)	Unclear risk	Randomized according to a scheme, actual method of scheme not discussed
Allocation concealment (selection bias)	Unclear risk	Allocation performed by pharmacist, such that half received AZA and half placebo<br>Method not described
Blinding of participants and personnel (performance bias)	Low risk	Double-blinded<br>"Only the pharmacist dispensing the tablets knew whether azathioprine or placebo had been selected for a given case"
Blinding of outcome assessment (detection bias)	Low risk	Double-blinded<br>"Only the pharmacist dispensing the tablets knew whether azathioprine or placebo had been selected for a given case"
Incomplete outcome data (attrition bias)	Unclear risk	Patients that withdrew from the study prematurely were not discussed
Selective reporting (reporting bias)	Low risk	All outcomes described in the methods section were reported in the results
Other bias	Low risk	The study appears to be free of other sources of bias

Young 2024 - iBaSS

Bias	Authors' judgement	Support for judgement

Random sequence generation (selection bias)	Low risk	"The randomisation sequence was generated using ALEA randomisation software (FormsVision BV, Abcoude, The Netherlands)"
Allocation concealment (selection bias)	Low risk	"UHS pharmacy department managed treatment sequence allocation to maintain the blinding of the study team. Pharmacy staff and participants were asked not to disclose theallocated adalimumab product to the clinical trial physicians during the trial."
Blinding of participants and personnel (performance bias)	High risk	Single blind study
Blinding of outcome assessment (detection bias)	High risk	Single blind study
Incomplete outcome data (attrition bias)	Low risk	Slightly more people discontinued the biosimilar in phase 1.
Selective reporting (reporting bias)	Unclear risk	Crossover study without differentiation between phase 1 and 2 results. Registered as EudraCT 2018-004967-30
Other bias	Unclear risk	Baseline characteristics are reported per protocol and there appear difference in disease behaviour and previous bowel resection between groups.
